# Efficient prediction of human protein-protein interactions at a global scale

**DOI:** 10.1186/s12859-014-0383-1

**Published:** 2014-12-10

**Authors:** Andrew Schoenrock, Bahram Samanfar, Sylvain Pitre, Mohsen Hooshyar, Ke Jin, Charles A Phillips, Hui Wang, Sadhna Phanse, Katayoun Omidi, Yuan Gui, Md Alamgir, Alex Wong, Fredrik Barrenäs, Mohan Babu, Mikael Benson, Michael A Langston, James R Green, Frank Dehne, Ashkan Golshani

**Affiliations:** School of Computer Science, Carleton University, Ottawa, Canada; Department of Biology, Carleton University, Ottawa, Canada; Banting and Best Department of Medical Research, University of Toronto, Toronto, Ontario Canada; Department of Electrical Engineering and Computer Science, University of Tennessee, Knoxville, Tennessee USA; Department of Pediatrics, Gothenburg University, Gothenburg, Sweden; The Centre for Individualized Medication, Linköping University, Linköping, Sweden; Department of Biochemistry, Research and Innovation Centre, University of Regina, Regina, Saskatchewan Canada; Department of Systems and Computer Engineering, Carleton University, Ottawa, Canada

**Keywords:** Protein-protein interactions, Computational prediction, Human proteome, Massively parallel computing, Personalized medicine, Interactome, Network analysis

## Abstract

**Background:**

Our knowledge of global protein-protein interaction (PPI) networks in complex organisms such as humans is hindered by technical limitations of current methods.

**Results:**

On the basis of short co-occurring polypeptide regions, we developed a tool called MP-PIPE capable of predicting a global human PPI network within 3 months. With a recall of 23% at a precision of 82.1%, we predicted 172,132 putative PPIs. We demonstrate the usefulness of these predictions through a range of experiments.

**Conclusions:**

The speed and accuracy associated with MP-PIPE can make this a potential tool to study individual human PPI networks (from genomic sequences alone) for personalized medicine.

**Electronic supplementary material:**

The online version of this article (doi:10.1186/s12859-014-0383-1) contains supplementary material, which is available to authorized users.

## Background

Protein-protein interactions (PPIs) are essential molecular interactions that define the biology of a cell, its development and responses to various stimuli. Physical interactions between proteins can form the basis for protein functions, communications, and regulation and controls within a cell. Such interactions can result in the formation of protein complexes that perform specific tasks. Similarly, internal and external signals are often realized and communicated through the formation of stable or transient PPIs. Due to their central importance to the integrity of communication networks within a cell, PPIs are thought to involve important targets for drug discovery [1] and are linked to a number of cellular conditions and diseases [2].

Our current knowledge of global PPI networks in different organisms is hindered by the constraints and limitations of existing experimental techniques amenable to high throughput PPI studies, such as yeast-two-hybrid (Y2H) and affinity purification combined with mass spectrometry (APMS). While both of these techniques have been successfully applied to global PPI detection in the yeast, *Saccharomyces cerevisiae* [3-6], they suffer from significant shortcomings highlighted by the lack of overlap observed between the PPI data in different reports. The two benchmark large-scale yeast APMS investigations have less than 25% overlap and this overlap is even less for the two classic Y2H projects [7]. Only 24 PPIs are shared between all four studies, further highlighting the gap in our understanding of global PPI networks. Although recent technical improvements are expected to increase the confidence of the detected PPIs and hence fill some of the current gap of knowledge, increasing the coverage and quality of PPI networks remains an important challenge [3,7-10].

Computational tools offer time and cost effective alternatives to traditional wet-lab PPI detection tools. They may also be used as “filters” to increase confidence in data derived from wet-lab experiments [7,11]. Like other techniques, most computational tools also suffer from notable deficiencies. For example, most computational methods rely heavily on previously reported data. Assuming that there are inherent discrepancies in the training data, the accuracies of such tools to detect new interactions are often questionable. Moreover, novel interaction domains or motifs are likely to be missed by methods that rely heavily on the structures or other high-level features of protein pairs known to interact. Another major shortcoming of computational tools is that they are often too computationally intensive, making them impossible to use for proteome-wide analysis. To date, no comprehensive all-against-all analysis of the entire human PPI network has been possible.

A small number of large-scale computational PPI prediction methods have recently been published (e.g. [12-14]). Although these methods have provided important contributions to the field, they are not applicable to the entire human proteome due to computational complexity, availability of input protein features, or unacceptably high false positive rates. For example, a recent study by Elefsinioti *et al.* examined five million protein pairs and predicted 94,009 “high confidence” interactions [13]. Given a conservative estimate of 22,000 human proteins, leading to 242 million possible pairs, Elefsinioti *et al.* have examined only 2% of the potential interactome while others have examined just over 7% [12] and 12.4% [14] of the total interactome. Presumably these methods were limited to examining only small subsets of protein pairs due to computational complexity (i.e. runtime) or the availability of input protein features. For example, the method of Elefsinioti *et al.* [13] requires 18 complex features for each protein relating to annotated function, sequence-derived attributes, and network structure. Likewise, the method of Zhang et al. [14] requires structural information for both proteins in the putative interaction and is therefore only applicable to 13,000 human proteins (even with homology-based models). When considering protein pairs rather than individual proteins, approximately 50% sequence coverage results in an examination of at most 25% of the possible PPIs. In fact, Zhang et al. report that they were able to develop models for 36 million interactions, representing 12.4% of the 242 million possible interactions. Even if these methods could be applied to all human protein pairs, typical false positive rates will render existing methods unusable on larger data sets. For example, considering that the method of Elefsinioti *et al.* [13] predicts 94,009 “high confidence” interactions among only 1.6% of protein pairs, then we can reasonably expect nearly 6 million “high confidence” predicted interactions if their method were to be applied to the entire human proteome. This is an order of magnitude higher than the largest current estimate of the true size of the human interactome [13], leaving the experimenter to weed through a multitude of false positive predictions to find the few true interactions. Likewise, using a previously published computational method [15], Zhang et al. recently reported [14] a false positive rate implying 41.2% precision, and their recall over an independent test set of 24,000 newly reported PPIs is less than 7%. Consequently, there is a need for the development of efficient tools that are readily amenable to proteome-scale PPI prediction. This is especially important as the field of personalized medicine will benefit tremendously from a fast and accurate method that can predict the global PPI maps of different individuals from their genomic sequences alone.

A subset of cellular PPIs is mediated by defined short, linear polypeptide sequences [16-18]. Leveraging this fact, a number of computational tools have been developed to detect PPIs solely on the basis of primary sequence [11,19,20]. Such approaches do not rely on known structures or other protein features that are not easily deduced from primary protein sequences, and are thus, in principle, able to interrogate portions of the proteome that are inaccessible to other methods. Some of their predictions have been confirmed by tandem affinity purification [19], *in vitro* binding assays [21], and *in vivo* functional analysis [22]. An added benefit of sequence-based PPI prediction is that short polypeptide sequences in one organism can be used to predict PPIs in another [23]. We note that, while the wide applicability of sequence-based PPI prediction methods is clearly a strength, in not using structural predictions, such techniques may be unable to account for structural features such as binding site accessibility or widespread contacts between non-contiguous residues.

We have developed a computational tool termed the Protein Interaction Prediction Engine (PIPE) that uses co-occurrence of short polypeptide regions to detect novel PPIs in *S. cerevisiae* [19]. Although PIPE was able to analyze potential PPIs within certain proteomes, applying this tool to more complex proteomes remained infeasible due to computational complexity. Analyzing the ~242 million protein pairs in the human proteome was estimated to require approximately 6.3 million CPU-hours of computation. In order to study the human PPI network, we developed a new Massively Parallel (MP) version of PIPE, which we call MP-PIPE. MP-PIPE overcomes some of the limitations of existing methods through computational acceleration of the algorithm (speed) and improved precision. We present a comprehensive all-against-all (pair-wise) analysis of the human proteome and study its biological properties. We then demonstrate the accuracy and utility of the MP-PIPE inferred interactome using a range of functional assays.

## Results and discussion

### MP-PIPE performance and scalability enables computational scan of entire human proteome

**Figure 1:**
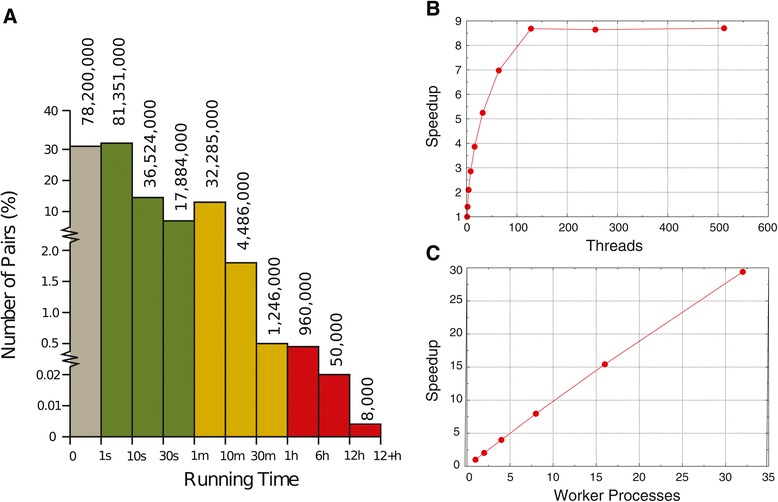
**MP-PIPE benchmark performance. A)** Distribution of running times for human protein-protein interaction prediction. Numbers above bars indicate approximate number of protein pairs with a running time within the given range. **B)** Performance for different numbers of threads per worker on the large cluster. Average running times for 5,000 random protein pairs (small number of threads) or 50,000 random protein pairs (large number of threads), using one worker process. **C)** Performance for different numbers of workers on the large cluster. Average running times for 500,000 random protein pairs, using 512 threads per worker.

One of the main issues when predicting human protein interactions on a large scale, which does not occur for simpler organisms such as *S. cervisiae* or *C. elegans*, is the complexity of the human proteome. More precisely, when predicting human protein interactions using previous methods [22], the compute time to process a single human protein pair can vary between several seconds and more than 12 hours. This effect has so far only been observed for the human protein interactions. Our previous method [22] was ranked highly in terms of prediction accuracy in an independent comparison study [24]. However, it would be unable to process all human protein pairs in our lifetime (approximately 6.3 million CPU-hours of computation). Therefore, we developed an algorithm called MP-PIPE, capable of performing global PPI analysis of the human proteome. Although the task of performing a proteome-wide, all-to-all prediction within the human proteome is still extremely computationally expensive for MP-PIPE, it still remained a feasible task and was completed within three months though massive parallelization. From a Computer Science perspective, the main challenge is the massive load imbalance of the parallelization. As shown in Figure 1A, for the vast majority of protein pairs, protein interaction prediction can be performed in seconds. However, for some protein pairs, the process takes minutes or hours, more than 12 hours in 8,000 extreme cases. Solving this load imbalance in an efficient manner is the main computational contribution of MP-PIPE.

In the following, we discuss the runtime performance of MP-PIPE on different hardware architectures which eventually enabled us to perform global PPI analysis of a human cell within three months. More precisely, we tested our MP-PIPE solution on three different compute clusters. These clusters included a six node cluster with 24 total compute cores (small cluster), a 32 node cluster with 128 total compute cores (medium cluster), and a 50 node cluster with 6,400 total hardware supported threads (large cluster). The performance of MP-PIPE was initially tested on a single large cluster node with varying numbers of threads, and then in a second test we increased the number of nodes (see details in Methods section). The test data set consisted of 50,000 random protein pairs. However, this data set proved to be too large to compute using a small number of threads, so a subset containing 5,000 random pairs was used to examine the runtime performance of the code with 1–16 threads and then the full 50,000 pair data set was used for tests with 16 or more threads. For those test cases that were performed over the smaller 5,000 pair subset, runtimes were extrapolated to estimate the runtime over the full 50,000 pair dataset. The results are shown in Figure 1. The speedup curve shown in Figure 1B shows a dramatic performance improvement using up to 128 threads and then a slight improvement from there up to 512 threads. We found that using more than 512 threads creates memory problems. For the second test, we increased the number of large cluster nodes used, where each node ran one MP-PIPE worker with 512 worker threads. The results are shown in Figure 1C. The performance of MP-PIPE scales almost linearly as the number of compute nodes increases. This scalability property of MP-PIPE enabled us to perform global PPI analysis of the human proteome within three months.

### Verification of MP-PIPE against experimental data

As with other PPI prediction methods, MP-PIPE relies on previously reported interaction data to make its predictions. The quality of the predictions made is inherently determined on the quality of this input data. To determine the prediction accuracy of MP-PIPE for human PPIs we conducted a leave-one-out (LOO test) test of MP-PIPE using the 41,678 experimentally verified, high-confidence human PPIs taken from BioGrid and 100,000 randomly chosen negative protein pairs (assumed to not interact). Choosing negative data in this way avoids sources of bias introduced by other methods (e.g. choosing pairs of proteins that do not appear in any BioGrid records may bias the negative set towards membrane proteins not readily amenable to experimental verification techniques [25,26]). The LOO tests were conducted as follows: MP-PIPE was run 41,678 times, one for each experimentally verified interacting protein pair (A, B). For each test run (A, B), we removed the known interaction (A, B) from the database. In this manner, we create a state where MP-PIPE is not aware of the experimentally verified PPI (A, B), as if that interaction had not been measured yet. We then asked MP-PIPE to predict whether or not proteins A and B interact. The same was then done for the negative set of randomly selected protein pairs that were expected to not interact. Once finished, the 141,678 total MP-PIPE predictions made were sorted by their PIPE score. Examining this sorted list allows us to set our decision threshold operating point. Given any “accept threshold” (see Methods for details on these thresholds), we can then see how many false positives and negatives were produced during our 141,678 test runs. Given the expected ratio of 100 non-interacting protein pairs for each interacting protein pair, typically a threshold that achieves an extremely high specificity (99.95%) is chosen in order to minimize out false positive rate. At the chosen operating point, MP-PIPE produced 9,586 true positives (TP), 99,950 true negatives (TN), 50 false positives (FP) and 32,092 false negatives (FN) from the 141,678 total test predictions made. These results are summarized in the confusion matrix in Table 1.

**Table 1:** **Confusion matrix for the leave-one-out cross-validation tests used to determine the prediction accuracy of MP-PIPE**

	**Known interacting pairs**	**Assumed non-interacting pairs**	**Total**
**Predicted to Interact**	9,586 (TP)	50 (FP)	9,636
**Predicted not to Interact**	32,092 (FN)	99,950 (TN)	132,042
**Total**	41,678	100,000	141,678

Since the ratio of known interacting pairs to assumed non-interacting pairs in our test set (i.e. 41,678:100,000) is not representative of the true ratio expected among all protein pairs within the *H. sapiens* proteome, the results in the above confusion matrix require adjustment. This adjustment to account for the true prevalence of PPIs among all protein pairs leads to a more conservative and realistic estimate of predictive performance of MP-PIPE. We have used a ratio of 100 non-interacting protein pairs per interacting pair. We feel this is a more realistic estimate given the expected sparsity of the actual interaction network and the range of estimates reported in previous studies [24,27]. The ratio-adjusted confusion matrix adjusted for this ratio is shown in Table 2.

**Table 2:** **The ratio-adjusted confusion matrix the leave-one-out cross-validation tests used to determine the prediction accuracy of MP-PIPE**

	**Known interacting pairs**	**Assumed non-interacting pairs**	**Total**
**Predicted to Interact**	9,586 (TP)	2084 (FP)	11,670
**Predicted not to Interact**	32,092 (FN)	4,165,716 (TN)	4,197,808
**Total**	41,678	4,167,800	4,209,478

A wide variety of performance metrics are commonly used to assess PPI prediction methods. These are summarized and computed in Table 3 below.

**Table 3:** **Statistical performance metrics for MP-PIPE based on true negatives (TN), true positives (TP), false negatives (FN), and false positives (FP) seen in the leave-one-out cross-validation tests, corrected to use a positive: negative ratio of 1:100**

**Statistical measure**	**Definition**	**Value**
Specificity (True Negative Rate)	$\frac{TN}{FP+TN}$	0.9995
Sensitivity/Recall (True Positive Rate)	$\frac{TP}{TP+FN}$	0.2300
Precision	$\frac{TP}{TP+FP}$	0.8214
Accuracy	$\frac{TP+TN}{TP+FP+FN+TN}$	0.9919
F1 Score	$\frac{2TP}{2TP+FP+FN}$	0.3594
Matthews correlation coefficient	$\frac{TPxTN- FPxFN}{\sqrt{\left(TP+FP\right)\left(TP+FN\right)\left(TN+FP\right)\left(TN+FN\right)}}$	0.4322

This leave-one-out test was repeated with all homologs removed from our human dataset as in [24]. This reduced our protein sequence set from 22,513 to 14,867 and our experimentally verified human PPI set from 41,678 to 19,588 pairs. Removing homologs at the 40% identity level effectively removes all protein isoforms from our LOO performance assessment. This leads to a conservative estimate of performance as not removing the homologs could potentially inflate the reported statistical performance figures [23,24]. As can be seen in the figure in Additional file 1 (pink line), the recall of our method is slightly reduced when homologous proteins are removed from our dataset, however if we adjust our decision threshold to maintain a recall of 23%, we still achieve a precision of 69.1%.

### All-against-all (pair-wise) scan of the human proteome

After three months of 24/7 computation on the 50 fully dedicated nodes of the large cluster (plus additional computation on the medium cluster), MP-PIPE completed the scan of the human proteome. With the chosen operating point as described in the previous section, MP-PIPE predicted 172,132 protein interactions. Of these high confidence predictions, 132,710 protein interactions have never been reported previously. Given that 41,678 human protein interactions are known (previously reported) and were included in the MP-PIPE database and would therefore be predicted to interact, MP-PIPE has potentially more than quadrupled our knowledge of the human interaction network. At the chosen operating point, MP-PIPE data covers more than one fifth of the estimated human PPI landscape. In comparison, Elefsinioti *et al.* [13] have examined 2% of the interactome while others have examined just over 7% [12] and 12.4% [14] of the total interactome. The list of the reported interactions is found in the table in Additional file 2. The list is ordered according to PIPE score, where higher values represent higher confidence levels for an interaction. Distribution of run time for different human protein pairs is illustrated in Figure 1A. The length of the query proteins does not appear to correlate with runtime (data not shown). The analysis performed on MP-PIPE’s predicted 172,132 interactions throughout the rest of this study will cover both the known 41,678 and novel 132,710 interactions, unless stated otherwise.

**Figure 2:**
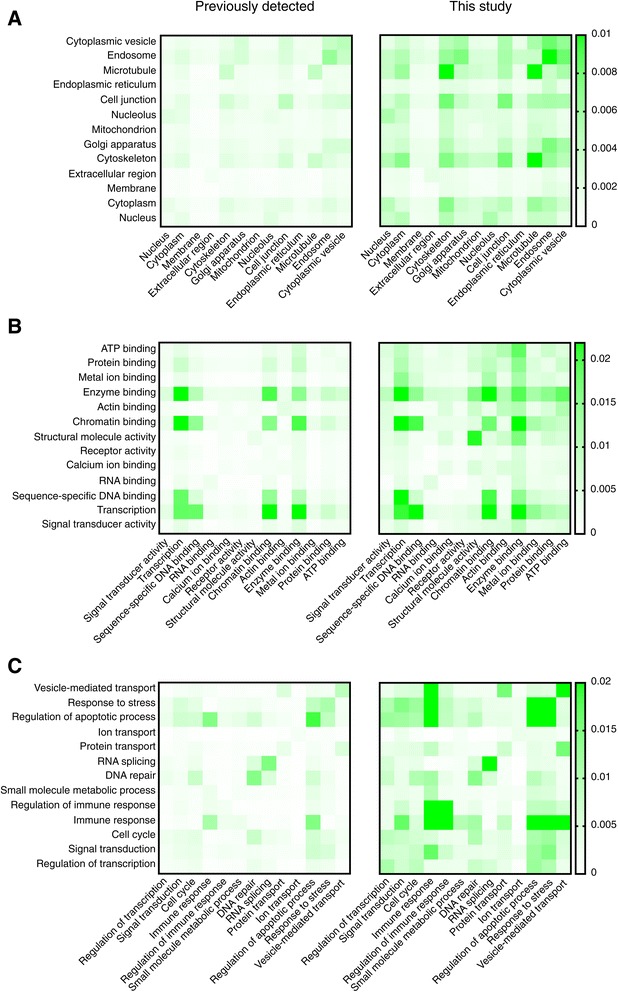
**Distribution of the interacting protein pairs on the basis of subcellular localization (A), molecular function (B) and cellular process (C) for both previously detected interactions and interactions unique to this study normalized by the number of possible pairs with both GO terms.** The overall co-occurrence (association) for pairs that were previously not reported is similar to that of previously reported interactions. The observed enrichment for certain categories within predicted interactions may represent new association or cross-communication. For example, in panel B, a co-occurrence (association) for “metal ion binding” and “transcription” is observed for predictions that were not previously reported. Similarly, in panel C, “immune response” and “signal transduction” have a more profound association among the predicted interacting pairs in this study.

Besides leave one out cross-validation, another standard method for evaluating PPIs is to check whether the proteins pairs predicted to interact are co-located within the same cellular component, have the same molecular function, are involved in the same biological process or have a common third party interacting partner. The results of this analysis are shown in Table 4 and Figure 2. The overall profiles for the predicted interacting pairs that have not been detected before, on the basis of cellular localization, process and function resembles that of previously reported pairs (Figure 2). Certain differences however are noticeable. For example, a new association for “metal ion binding” and “transcription” is observed for the predicted interactions and not for those that have been previously reported (Figure 2B). Similarly, there is a strong association between “immune response” and “signal transduction” for the predicted interactions (Figure 2C). As indicated in Table 4, the percentage of predicted interacting protein pairs that have similar function, occur in the same cellular component and participate in the same cellular process is 20.6%, which is consistent with the percentage for previously reported protein pairs (35.0%). In contrast, only 0.8% of randomly selected protein pairs share these three traits. It is important to note that the PIPE algorithm has no previous knowledge of the protein location, molecular function of proteins, or the biological processes in which they are involved. Such an association for protein pairs predicted by MP-PIPE further highlights the ability of this method to predict interactions that can be supported by independent parameters. Such contextual information can also be used to assign an independent degree of confidence for PPI predictions. For example, higher confidence might be assumed for a protein pair where both proteins occur in the same location and share the same GO term for a cellular process. This information is presented in the table in Additional file 2 and can be used to form a priority list of interactions for further biological analysis.

**Table 4:** **Percentages of** ***Homo sapiens*** **pairs in which both partners share the same GO SLIM annotation as well as third party interactions**

	**Derived from GO annotation**				**Third party interaction**
	**Cellular component (CC)**	**Molecular function (MF)**	**Biological process (BP)**	**CC & MF & BP**	
(a) Random *H. sapiens* pairs	19.7%	8.2%	2.8%	0.8%	0.4%
(b) Previously reported *H. sapiens* interactions	77.2%	64.4%	46.6%	35.0%	59.2%
(c) Predicted *H. sapiens* interactions identified in this study	64.1%^*^	43.9%^*^	30.8%^*^	20.6%^*^	23.9%^*^

^*^P-value <1e-16.

**Figure 3:**
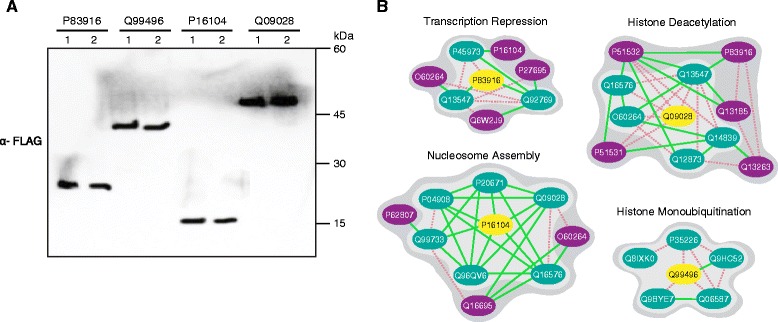
**Affinity purification experiments using P83916 (CBX1), Q99496 (RNF2), P16104 (H2AFX), and Q09028 (RBBP4) as baits. (A)** Immunoblot confirming the expression of the indicated FLAG-tagged chromatin proteins using antibody against the 3X FLAG epitope. Two independent FLAG tag constructs of each chromatin related protein was constructed for affinity purifications to eliminate background contaminants and to uncover highly reproducible interactions. Molecular masses (kDa) of marker proteins by SDS-PAGE are indicated. **(B)** Representative functional clusters identified from affinity purification data (light gray) and expanded by MP-PIPE predictions (dark gray). Tagged-baits are shown by yellow nodes (ellipses). Blue nodes represent co-purifying proteins identified through affinity purification. Purple nodes represent proteins added to the functional clusters through MP-PIPE predictions. Red dashed edges (lines) represent previously reported binary interactions identified in literature experimental data and green solid edges represent novel (not previously reported) MP-PIPE binary interaction predictions.

In addition to the above cross-validation, we independently evaluated the competence of our predictions by evaluating experimental data gathered using the Lentivirus-delivered, Gateway-compatible affinity Tagging System coupled with Mass Spectrometry (LGTS-MS) approach that has been recently developed for the identification of PPIs in mammalian cell lines [28]. The LGTS system uses a versatile affinity (VA)-tag constructed in-frame with a Gateway cassette consisting of 3x Flag, 6x His, and 2x Streptactin (Strep) epitopes, with Flag and His separated by dual TEV protease cleavage sites for efficient affinity purification [28]. Using this approach, we stably expressed four (CBX1 (P83916), RNF2 (Q99496), H2AFX (P16104), and RBBP4 (Q09028 )) C-terminal affinity tagged chromatin-related proteins in human embryonic kidney (HEK) 293 cells that play an important role in the epigenetic control of chromatin structure and gene expression, transcriptional repression, nucleosome remodeling, and chromatin assembly [29-33] (Figure 3A). These tagged proteins were affinity-purified in one step on anti-FLAG resins and the interacting proteins were identified by tandem mass spectrometry.

As expected, we recovered both the bait and several well-known interacting protein partners, such as the interaction between the tagged histone binding protein, RBBP4 (Q09028) and subunits of the core histone deacetylase complex (HDAC1 (Q13547) and RBBP7 (Q16576)), confirming the overall efficacy of the protein purification procedure employed in the identification of co-purifying interacting proteins (Figure 3B; Additional file 3). Consistent with the biological expectation, these co-purifying proteins were enriched for more chromatin related functions such as chromatin organization, binding and assembly; nucleosome assembly; histone ubiquitination; and transcriptional regulation. Examples of functional clusters identified through the LGTS-MS based method are shown in Figure 3B. To investigate MP-PIPE’s ability to explain the observed LGTS-MS data, we computed the precision and recall of MP-PIPE predicted interactions. This is summarized in Table 5 where reachable proteins are defined as those proteins that interact directly or through one or two intermediary proteins. This accounts for the fact that a bait and prey observed to co-purify in a LGTS-MS experiment may, in fact, interact indirectly through one or more intermediary proteins. For the four baits, previously known PPI interactions (high confidence literature data) can only explain on average 10.89% (recall) of the co-purifying proteins (prey). Using MP-PIPE predictions increases our recall by ~3-fold (29.31%) while maintaining comparable precisions.

**Table 5:** **Overlap of co-purifying proteins identified through LGTS-MS with previously reported (known) interactions and MP-PIPE predictions**

		**Reachable proteins**		**Prey reached**		**Recall** ^**1**^		**Precision** ^**2**^	
**Bait**	**# of prey**	**Known**	**MP-PIPE**	**Known**	**MP-PIPE**	**Known**	**MP-PIPE**	**Known**	**MP-PIPE**
Q09028	301	112	201	56	99	18.60%	32.89%	50.00%	49.25%
P83916	474	91	244	59	178	12.45%	37.55%	64.84%	72.95%
P16104	209	39	207	11	82	5.26%	39.23%	28.21%	39.61%
Q99496	292	16	24	13	15	4.45%	5.14%	81.25%	62.50%
**Total**	**1276**	**258**	**676**	**139**	**374**	**10.89%**	**29.31%**	**53.88%**	**55.33%**

^1^Recall calculated as reached/# of prey.

^2^Precision calculated as reached/reachable.

**Figure 4:**
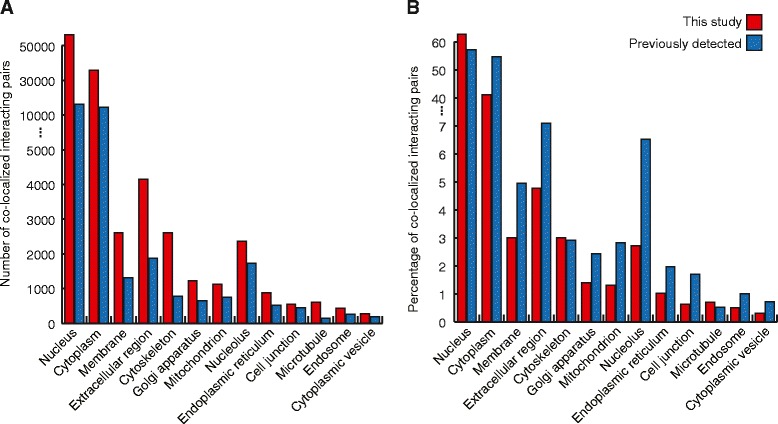
**Number (A) and percentage (B) of co-localized interacting pairs by GO components in previously reported data compared with those reported in this study only.** Note that since proteins can have multiple tags, interacting pairs can be co-localized in several components and can be counted more than once.

Our predicted interactions appear to have a wide coverage of the human proteome. For instance, of the 22,513 human potential open reading frames included in this study, 11,194 were found in our prediction list (i.e., form at least one interaction) for a coverage of approximately 50%. Since a total of 172,132 interactions were predicted, on average there appears to be approximately 15 interactions for each protein found in the prediction list. As illustrated in Figure 4, approximately 32% of the predicted interactions occur in the nucleus, followed by 21% in the cytoplasm. In fact, the distribution of the identified interactions is very consistent with those of the previously reported (known) ones. This distribution is also in accordance with the previously reported PPI distribution in *S. cerevisiae* [6]. Of interest are membrane proteins, which although not readily amenable to experimental assays, received good coverage using MP-PIPE. The full range of biological processes and molecular functions is also well covered (Additional file 4). On the basis of expression level (obtained from ArrayExpress EMBL-EBI), 66.26% and 68.99% of highly and low expressed proteins, respectively, also appear in the list of PPIs. We also examined the interactions for fibroblast growth factors (FGFs) and cyclin-dependent kinases (CDKs) with fibroblast growth factor receptors (FGFRs) and regulatory inhibitors and activators of cyclin-dependent kinases, respectively. These represent examples of proteins that share high similarity in primary sequence and molecular function, yet have differences in substrate specificity and regulatory factors. Shown in the table in Additional file 5 there are clear differences in interacting partners for different members of FGF and CDK proteins. Altogether these observations suggest that our prediction method appears to be inclusive and specific, and is amenable to diverse set of proteins presenting a good coverage of the proteome.

### Network-wide analysis of hubs and betweenness centrality

In a PPI network, the degree of interaction for a target protein is believed to be a good indicator for the biological importance of that protein within the system [34,35]. Removal of the highly connected proteins or “hubs” appears to have a more profound effect on the integrity of the network by reducing the size of the largest connected module, than removal of random proteins [36]. We studied the top 10, 25 and 50 hubs with the highest number of interactions within our predicted PPI network, and observed very high enrichment for proteins that affect transcription and gene expression (Table 6). Transcription factors mediate differential genetic programming and hence are of central importance in developmental biology [37], responses to stimuli [38], disease progression [39], etc. Betweenness centrality is another topological feature of a network and evaluates the number of shortest paths that pass through a given node [40]. Therefore, high betweenness centrality for a protein represents the relative number of shortest paths that are associated with that protein. Consequently, proteins with high betweenness centrality are thought to play a central role in the cross-talk and communication between interconnected modules of a network by forming “traffic bottlenecks” for communication [41]. We evaluated the top 10, 25 and 50 proteins with highest betweenness centrality values within our predicted network. Consistent with the expected role of these proteins in signaling, we observed (Table 3) that they were highly enriched for proteins involved in intracellular communication (kinase activity and signaling), or for which communication is of central importance (regulation of cell death).

**Table 6:** **Enrichment of biological process for proteins with highest Hub Degree (Hubs) or Betweeness Centrality (B.C.) measurements (Top 10, Top 25, and Top 50)**

	**Biological process**	**Top 10**		**Top 25**		**Top 50**	
		**# prot**	**P-value**	**# prot**	**P-value**	**# prot**	**P-value**
**Hubs**	**Transcription regulation**	7	2.26E-06	16	3.15E-10	28	1.93E-14
	**Regulation of gene expression**	7	4.88E-06	16	1.69E-09	29	2.82E-14
**B.C.**	**Protein kinase activity**	2	5.99E-02	6	1.01E-07	20	8.61E-18
	**Regulation of cell death**	3	3.96E-05	11	7.60E-09	19	1.82E-12
	**Signaling**	4	5.49E-05	13	5.90E-09	33	7.45E-12

**Figure 5:**
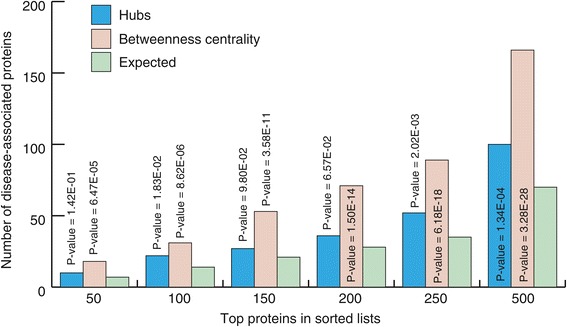
**Comparing the number of disease-associated proteins with high degree (hubs) and high betweenness centrality.** Proteins with highest betweenness centrality appear to be more enriched for disease proteins than hubs.

Centrality measurements are often used to predict the possible involvement of a protein in disease etiology and progression. Some studies suggest that hubs are likely enriched for disease proteins, whereas others incline towards betweenness centrality as a better indicator [42-46]. We therefore examined a possible relationship between the top 500 proteins with the highest degrees of centrality (hub and betweenness centrality) and their reported involvement in disease progression. As illustrated in Figure 5, both hub and betweenness centralities appear to be good indicators for disease proteins. However, betweenness centrality appeared to have a better correlation than hubs for disease proteins. A ranked list of top 500 proteins according to their centrality measures is reported in Additional file 6 (hubs) and the table in Additional file 7 (betweenness centrality). We note that the relationship between connectivity (with hubs having a higher connectivity) and disease is dependent on a variety of factors, particularly gene essentiality [45], which we have not investigated here in depth.

### The usefulness of the predicted interactions in biological investigations

Our computationally predicted interactome represents a comprehensive all-to-all interaction network in humans. This network generates a wide range of testable hypotheses concerning biological processes, and informs our understanding of the overall architecture of cellular function. Here, we demonstrate the usefulness of this new predicted interactome through prediction of gene functions, experimental verifications and analysis of putative protein complexes.

### Using the predicted human protein interaction network to assign breast cancer proteins

**Figure 6:**
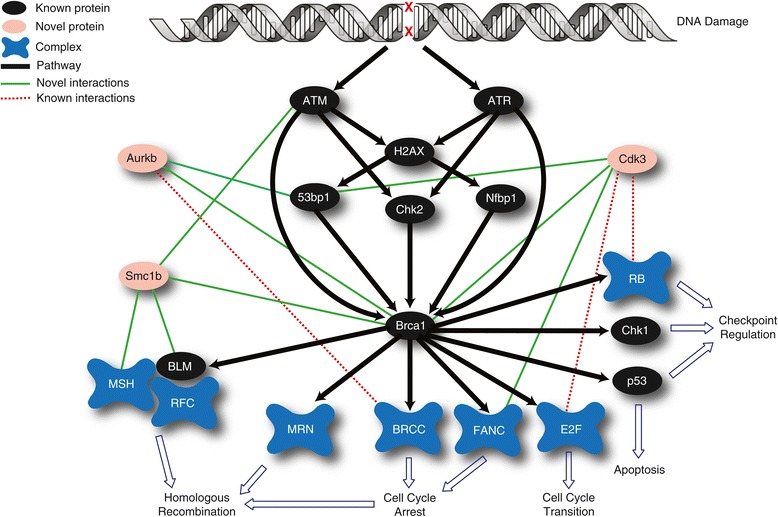
**Schematic diagram of breast cancer pathway.** BRCA1 plays a central role in breast cancer by connecting DNA damage and chromatin remodeling to downstream processes such as cell cycle progression and DNA repair. Black lines (edges) represent biochemical pathways, red and green edges are novel and known PPIs, respectively. Ovals and clouds represent proteins and protein complexes, respectively. Red ovals represent novel proteins that are associated with breast cancer pathway on the basis of the predicted interactions they form.

Breast cancer is the most commonly diagnosed form of cancer among women [47]. BRCA1 and to some extent BRCA2 are the two key genes associated with breast cancer progression. Breast cancer susceptibility has been related to a mutation of BRCA1 [48]. Carriers of BRCA1 (and some BRCA2) mutations have a 50-80% increased risk of developing breast cancer [49]. It is estimated that 10% of western women fall in this category [47]. While the tumor suppression property of BRCA1 is well investigated, the molecular mechanism of its activity in tumor prevention is not fully understood [50]. Figure 6 illustrates a brief overview of the breast cancer pathway where BRCA1 plays a central role. As illustrated, (see Figure 6) BRCA1 is directly associated with several cellular processes including chromatin remodeling, DNA damage checkpoint activation, DNA damage sensing, and DNA double stranded break (DSBs) repair. BRCA1 plays an essential role in delaying cell cycle progression by its DNA damage checkpoint activity. ATM phosphorylation of p53, a tumor suppressor protein, is mediated by BRCA1 and, in the presence of DNA damage, delays or arrests G1/S transition [51]. BRCA1 is important during S phase and G2/M checkpoint activation through its regulation of kinase activity of Chk1 [52]. Upon DNA damage, H2AX is phosphorylated by ATM and ATR and recruits MDC and RNF8 to the site of the damage. Subsequently, BRCA1 is translocated to the site of damage by ubiquitination of H2A through RNF8 and Ubc13 [47] and interacts with the Mre11/Rad50/Nbs (MRN) complex that is involved in double stranded DNA break repair [53]. Examining the proteins involved in the breast cancer pathway for PPIs, MP-PIPE predicted over 3,000 interactions, 424 of which (161 and 263 known and novel interactions, respectively) directly involve BRCA1 (P38398). Studying these interactions can expand our current understanding of the breast cancer pathway. A number of interesting factors were found to form novel interactions with multiple proteins associated with the breast cancer pathway, including CDK3, AURKB, and SMC1b (see Figure 6).

CDK3 is a cyclin-dependent kinase that functions in cell cycle progression and mitosis, and plays an essential role in G1/S transition through its activation of the E2F transcription factor family and G0/G1 transition by Rb phosphorylation [54]. E2F and Rb play a regulatory role in the transcription of BRCA1 [55], connecting CDK3 to BRCA1. In further agreement with the observed interactions, CDK3 has high expression levels in cancer cells and participates in cell proliferation and transformation by enhancement of ATF1 activity, a gene that physically interacts with BRCA1 ([56,57]). Furthermore, both BRCA1 and CDK3 are involved in cell cycle transition, further supporting a potential role for CDK3 in breast cancer.

AURKB has several functions during mitosis, including spindle assembly, chromosome segregation, and cytokinesis [58]. AURKB has high sequence similarity with AURKA, another protein of the Aurora kinase family, however they are reported to differ functionally from each other during mitosis [59]. It is shown that BRCA1 may be phosphorylated by AURKA, resulting in impaired function of BRCA1 in G2/M transition [60]. AURKB has a single reported interaction within breast cancer pathway through BRCC complex [61]. The interactions identified here add credibility to the involvement of this protein in breast cancer pathway.

SMC1B is a meiosis-specific protein involved in chromosome segregation during anaphase, synapsis, and recombination [62]. SMC1B is also part of the cohesin complex, which includes SMC1, SMC3, RAD21 and several other proteins [63]. The cohesin complex plays a role in several cellular processes such as DNA repair, gene expression regulation and chromosome segregation ([63,64]). Recent studies showed that several subunits of the cohesin complex are also important in DNA damage response [64]. In addition, SMC1b has been linked to neck and head cancer [65], further supporting a role for this protein in cancer.

We also examined the PPI network for mutations associated with resistance to breast cancer therapeutics doxorubicin and Trastuzumab. Individual mutant proteins were analyzed against the human proteome (one-against-all) for their PPIs at a recall of 23% at a precision of 82.1%. In this way, 5 personalized human PPI networks were predicted, each differing by a mutation in one gene only. The 5 PPI profiles were compared to that of their corresponding control networks. The list of these mutants is found in the table in Additional file 8. Four mutations, P04637a, P04637b, P04637c and P04637d, in p53 (P04637) protein have been linked to resistance to the chemotherapeutic breast cancer drug, doxorubicin [66]. The PPI profile for P04637b and P04637d was identical to that of the wild type. However, the other two mutants showed some differences. For example, P04626a and P04626c lost their interactions with the nuclear transcription factor Y, NFYC (Q13952) and the ubiquitin conjugating enzyme E2 L3 (P68036) involved in nuclear hormone receptors transcriptional activity, among others. Similarly, a truncated form of HER2 (P04626) is responsible for resistance against HER2-targeted breast cancer therapeutics such as Trastuzumab [67]. We observed that truncated-HER2 lost several PPIs including an interaction with a G-protein signaling RGS8 (P57771) which functions as an inhibitor of signal transduction, and an interaction with an early growth response protein ERG1 (P18146) involved in cell differentiation. Of interest, the truncated HER2 formed a new interaction with the tumor suppressor p53 protein. A possible explanation for this novel interaction for the truncated HER2 could be that segments of the deleted region might have physically hindered the availability of the region responsible for an interaction with p53 in the wild type form.

### Identification of novel molecular markers for seasonal allergic rhinitis

Glucocorticoids (GCs) have a key role in the treatment of patients with seasonal allergic rhinitis (SAR) and other allergic disorders [68]. Because of this and difficulties in evaluating treatment response based on clinical signs and symptoms, there is a need for protein markers to monitor that response. The identification of such markers is complicated by the involvement of a large number of inflammatory proteins in SAR [69]. We hypothesized that novel biomarkers could be identified among proteins predicted to interact with proteins belonging to known inflammatory pathways in SAR including the acute phase response pathway, complement signaling pathway and glucocorticoids receptor pathway [70,71].

Proteins from the acute phase response pathway, complement signaling pathway and glucocorticoids receptor pathway were extracted from the Ingenuity pathway analysis (IPA) software. Interactors of these proteins were selected from our predicted human PPI network. We included secreted, membrane and cytoplasmic proteins, but excluded nuclear proteins. We prioritized candidate biomarkers based on their number of known and predicted interactions with proteins known to be involved in SAR-associated responses. Next, we focused on proteins with a high number of predicted interactions.

From the literature we extracted 191 proteins that belong to the acute phase response pathway, complement pathway, and glucocorticoids receptor pathway (Additional file 9). These proteins formed the known set of SAR-associated proteins (SARp). From our predicted human PPIs, the proteins that interacted with SARp were determined. A total of 3334 proteins were found to interact with one or more SARp. We prioritized five new proteins with a high number of total and predicted interactions to SARp as candidate biomarkers, namely PRB1, PRB2, SFN, LYN and Akt2. Using ELISA, we analyzed these candidates in nasal fluid from 40 patients with SAR before and after GC treatment. This study represented protein expression analysis for 400 samples (5 proteins, before and after GC treatment, in 40 patients).

**Figure 7:**
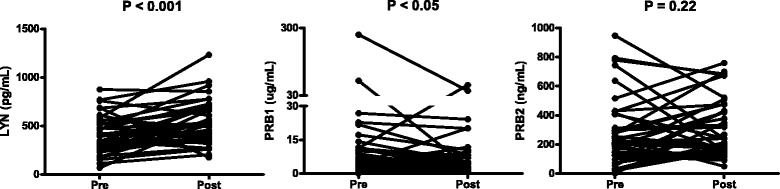
**Analysis of candidate proteins with ELISA.** Proteins were analyzed in nasal fluid from 40 patients with SAR before and after GC treatment. Pre, patients before GC treatment; Post, patients after GC treatment. LYN and PRB1 were differentially expressed before and after GC treatment with P-values of <0.001 and <0.05, respectively.

It was observed that after GC treatment LYN concentration increased from 396.1 ± 30.5 pg/mL to 537.7 ± 35.5 pg/mL (P-value <0.001). PRB1 decreased from 16.3 ± 7.0 ug/mL to 8.5 ± 2.1 ug/mL (P-value <0.05) (Figure 7). PRB2 was not differentially expressed before and after treatment, and SFN and Akt2 were not detectable in most samples. Differential protein expression for LYN and PRB1 provides a good evidence for the possibility of using these proteins as novel molecular markers for SAR. Altogether, the data presented here illustrates the suitability of the predicted PPIs for identifying potential new molecular markers for human conditions.

### The proteome-wide PPI network can identify translation genes

**Figure 8:**
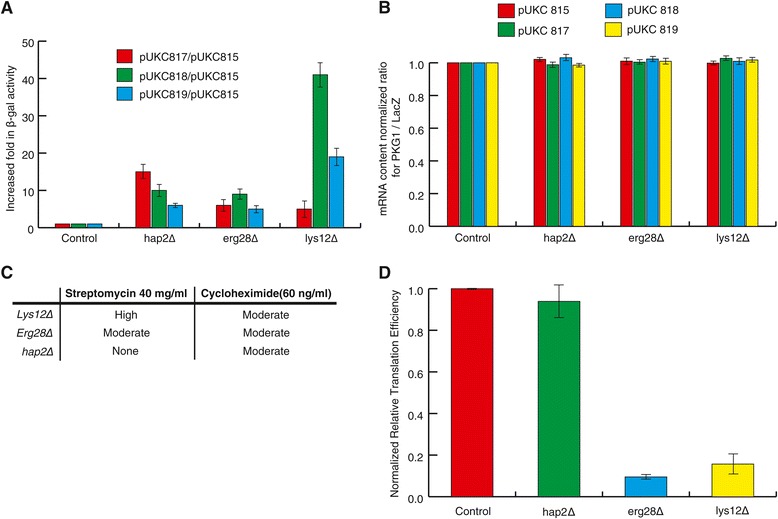
**Novel involvement of HAP2, ERG28 and LYS12 in translation. A)** The relative *β-galactosidase* activity is determined by normalizing the activity of the mutant strains carrying different stop-codon read through cassettes to the control construct (pUKC815 with no premature stop codon) in the wild type strain. **B)** The relative mRNA level is determined by normalizing the mRNA content of the mutant strains carrying different premature stop-codon expression cassettes to those in the wild type. **C)** Increased sensitivity of *hap2Δ, erg28Δ and lys12Δ* to different translation inhibitory drugs. Sensitivity of the wild type strain was used as a point of reference. Sensitivity was quantified as low, moderate and high with respect to that for the wild type strain. *hap2Δ, lys12Δ,* and *erg28Δ* show increased sensitivity to one or both streptomycin and/or cycloheximide. **D)** Effect of gene deletions on translation efficiency. Relative translation efficiency was measured using p416 plasmid containing Gal-inducible promoter in LacZ expression cassette normalized to mRNA content. Values are related to translation efficiency of the control strain set at 1.0.

The process of protein synthesis or translation is the process by which the genetic message embedded in mRNAs is sequentially read and converted into polypeptide sequences. Due to its absolute requirement for the survival of a cell, this process has remained highly conserved through the course of evolution. Our predicted interactome included novel interactions for five human proteins Q96DG6 (CMBL), Q08AM6 (VAC14), P23511 (NFYA), Q9UKR5 (ERG28) and P48735 (IDH2) with proteins known to play roles in the process of translation. To study the involvement of these five proteins in translation, we subjected their corresponding yeast homologs (AIM2, VAC14, HAP2, ERG28 and LYS12, respectively) to experimental analysis. First, we examined the effect of their deletion on stop-codon read-through using three different expression plasmids, pUKC817, pUKC818 and pUKC819 that carry premature stop codons UAA, UGA and UAG, respectively, within a *β-galactosidase* reporter gene. As evident from an increase in relative *β-galactosidase* activity shown in Figure 8A, the deletion of HAP2, ERG28 and LYS12 significantly altered the ability of ribosomes to detect all three stop codons. To confirm that the observed elevation of *β-galactosidase* was at the translation level, mRNA content of *β-galactosidase* was measured. No difference between relative content of *β-galactosidase* mRNAs was observed in deletion and control strains (Figure 8B).

We then further investigated the involvement of HAP2, ERG28 and LYS12 in translation by subjecting their deletion mutants to drugs that affect translation. *hap2∆, erg28∆* and *lys12∆* showed altered levels of sensitivity to streptomycin and cycloheximide (Figure 8C). Next, translation efficiency (rate) was measured using an inducible LacZ gene cassette on a p416 plasmid [72]. Deletion mutants for ERG28 and LYS12 had a drastic reduction in the rate of induced LacZ synthesis further linking ERG28 and LYS12 to translation (Figure 8D). Interestingly ERG28 is a well-characterized protein involved in ergosterol biosynthetic pathway, the relation of which to translation is not readily expected. However, in agreement with a link to translation, ERG28 was previously shown to physically interact with a polysome associated mRNA binding protein SLF1 [73], and a putative RNA helicase SPB4 that sediments with 66S pre-ribosomes [74]. Further, ERG28 is localized to ER membranes and a general link between sterol biosynthesis and translation has previously been proposed [75].

### Identification of protein complexes within the human interaction network

Protein complexes can be defined as a group of proteins that interact with each other to form a functional unit. Paracliques [76-78] can be computationally identified as a sub group of proteins within the interaction network with high degree of interconnectivity and may define putative complexes. Given the size of the human PPI network, prediction of paracliques requires advanced computational approaches to complete a thorough analysis within a reasonable timeframe. We have applied a novel graph theoretic approach to automatically identify paracliques within the network (see Methods for details). Our analysis led to a number of interesting predictions. For each paraclique, a statistical analysis of gene ontology (GO) term enrichment was performed. The table in Additional file 10 lists the top GO term for each paraclique along with a P-value for the observed enrichment. For example, Paraclique 1359 is a complex of six proteins with 13 interactions (Additional file 11: Figure A). O00151 (PDLIM1) is a cytoskeletal protein that acts as an adapter to bridge other proteins (like kinases) to the cytoskeleton. P20929 (NEB) is a muscle protein involved in maintaining the structural integrity of sarcomeres and membranes associated with the myofibrils (F-actin stabilization). The rest of the members (P08670 (VIM), P14136 (GFAP), P17661 (DES) and P41219 (PRPH)) are intermediated filament proteins. On the basis of GO enrichment (P-value 6.5E-07), one may conclude that the activity of this complex is associated with cytoskeleton and structural integrity of the cell.

Paraclique 1409 is a complex of six proteins with 14 interactions (Additional file 11: Figure B). Q02246 (CNTN2) is involved in cell adhesion and the remaining proteins (O94779 (CNTN5), Q02246 (CNTN2), Q12860 (CNTN1), Q8IWV2 (CNTN4), Q9P232 (CNTN3), and Q9UQ52 (CNTN6)) are involved in cell surface interaction during nervous system development. On the basis of GO enrichment, we can assign this complex to cell adhesion (P-value 2.2E-10).

Paraclique 2164 is a complex of five proteins with 10 interactions (Additional file 11: Figure C). Three of its members (P32298 (GRK4), P34947 (GRK5) and P43250 (GRK6)) are G protein-coupled receptor kinase and the remaining two (Q9NP86 (CABP5) and Q9NZU8 (CABP1)) are calcium-binding proteins. Considering the fact that biological interaction between G-protein coupled receptor and calcium-binding proteins has been widely reported and seems essential in signaling pathways, one may conclude that this complex plays a role in G-protein coupled signaling pathway, a claim which is supported by enriched Gene Ontology term (P-value 3.75E-08).

### Limitations and future work

While MP-PIPE represents a significant step forward towards computing a complete human interactome, there remain a number of limitations which lead us to future work. In order to operate at a reasonable precision rate, we have tuned our decision thresholds to be extremely conservative, resulting in a limited sensitivity of 23%. Future work will examine ways to continue to increase sensitivity/recall without sacrificing our false positive rate. Where MP-PIPE has advantage over structure-based methods is in coverage: MP-PIPE requires only sequence as input and is therefore applicable to all protein pairs. However, in future work we will examine ways to capitalize on the rich information encoded in protein structure when such inputs are available. At present, this represents only a small fraction of protein pairs, however, this proportion is expected to grow with ongoing large-scale protein structure determination initiatives. As with all computational methods, another potential limitation in prediction accuracy is the quality of input data used to train MP-PIPE. As more experimental data of higher quality becomes available, we expect MP-PIPE to also become more accurate. Lastly, we are continuing to apply parallelization and algorithmic optimizations to MP-PIPE to further reduce runtimes for whole-proteome scans. This will be critical if we are to investigate large numbers of organisms for comparative studies, or if we wish to compute personalized interactomes, accounting for the multitude of genetic variations that make each person’s interactome unique.

## Conclusions

In this study, we present a comprehensive pair wise analysis and prediction of the entire human PPI network using the principles of short co-occurring polypeptide regions as mediators of PPIs. Through this massive computational analysis, we predict approximately 170,000 PPIs, of which 140,000 have not been reported previously. The distribution of the novel PPIs on the basis of sub-cellular localization, molecular function and biological process are very similar to those of previously reported interactions, highlighting the reliability of our predictions. Moreover, we demonstrate that MP-PIPE predictions can effectively explain experimentally observed LGTS-MS interaction data (recall 29.31%, precision 55.33%). Our predictions are useful for understanding cellular biology as a whole, with approximately 8,000 protein complexes in our inferred interaction network. Furthermore, specific processes can be successfully interrogated using our new predictions: on the basis of inferred interactions we predict and experimentally confirm novel functions for proteins involved in translation, and identify new molecular markers for seasonal allergic rhinitis. Our analysis highlights the usefulness of the predicted PPIs for functional analysis of the human proteome. The speed associated with this approach sets the path for investigating the PPI map for individual humans in a timely fashion. Personal (specific to an individual) PPI maps may improve our knowledge of network and personalized medicine.

## Methods

### Sequential PIPE algorithm

For a given organism (e.g. *S. cerevisiae*, *C. elegans*, or human), the PIPE algorithm relies on a database of known and experimentally verified protein interactions. For example, for the 22,513 human potential open reading frames included in the current study, only 41,678 high confidence interactions are known (out of 253,406,328 possible protein pairs). Since experimental verification can have large numbers of false positives (up to 40%, see e.g. [19]), the PIPE database is carefully constructed to avoid false data and stores only protein interactions that have been independently verified by multiple experiments. The database represents an interaction graph *G* where every protein corresponds to a vertex in *G* and every interaction between two proteins *X* and *Y* is represented as an edge between *X* and *Y* in *G*. The remainder of this section outlines how, for a given pair (*A, B*) of query proteins, our PIPE method predicts whether or not *A* and *B* interact.

In the first step of the PIPE algorithm, protein *A* is split up into overlapping fragments of size *w*. This can be thought of using a sliding window of size *w* across protein *A*. For each fragment *a*_*i*_ of *A*, where 0 < = *i < =* |*A*| *- w +1*, we search for fragments "similar" to *a*_*i*_ in every protein in graph *G*. A sliding window of size *w* is again used on each protein in *G*, and each of the resulting protein fragments is compared to *a*_*i*_. For each protein that contains a fragment similar to *a*_*i*_, all of that protein's neighbors in *G* are added to a list *R*. To determine whether two protein fragments are similar, a score is generated with the use of the PAM120 substitution matrix. If the similarity score is above a tuneable threshold then the fragments are said to be similar or to “match” (see pseudocode below). In the next step of the PIPE algorithm, protein *B* is split into overlapping fragments *b*_*j*_ of size *w* (0 < = *j < =* |*B*| *- w +1*) and these fragment are compared to all (size *w*) fragments of all proteins in the list *R* produced in the previous step. We then create a result matrix *H* of size *n***x***m*, where *n = |A|* and *m = |B|* and initialize it to contain zeroes. For a given fragment *a*_*i*_ of *A*, every time a protein fragment *b*_*j*_ of *B* is similar to a fragment of a protein *Y* in *R*, the cell value at position (*i, j*) in the result matrix is incremented. The result matrix indicates how many times a pair (*a*_*i*_, *b*_*j*_) of fragments co-occurs in protein pairs that are known to interact. It is based on this matrix that the query proteins are predicted to interact or not. The following explains the basics of the algorithm in pseudocode:

A modified median filter, which simply sets a cell’s value to 1 if most of the neighbouring cells are greater than zero and zero otherwise, is applied and the two query proteins were predicted to interact if the average cell value was above a set threshold. By varying this threshold, a range of precision-recall values may be obtained (see Additional file 1). Note that throughout this paper, for our analysis a prevalence of 1 PPI per 100 protein pairs is consistently assumed for our results, as well as for comparison to other results as was done in [24]. Recall measures the proportion of true interactions that will be detected. Precision measures the proportion of predicted interactions that correspond to true interactions. For our leave-one-out cross-validation experiments (as described in the ‘Verification of MP-PIPE Against Experimental Data’ section), our 41,678 high confidence positive PPIs are taken from BioGrid [25]. Random protein pairs not previously reported to interact were used for our negative interaction data. This is considered to be a conservative approach when assessing prediction accuracy [26].

### MP-PIPE overview

The MP-PIPE (massively parallel PIPE) system is a massively parallel, high throughput protein-protein interaction prediction engine and is the first system that is capable of scanning the entire protein interaction network of complex organisms such as human. In order to achieve that goal, large numbers of concurrent PIPE instances need to be executed on a large-scale parallel compute cluster. This created two major challenges.

The first problem was the lack of scalability that made it difficult for large numbers of PIPE instances to effectively take advantage of all available computational resources without massive load imbalances. This load-balancing problem was not as significant in simpler organisms, such as *S. cerevisiae* and *C. elegans*, but lead to a large amount of unused resources when making predictions on more complex organisms such as human. Interestingly, the number of human proteins and protein pairs is not exceptional and simpler organisms such a *C. elegans* actually have more proteins and protein pairs than human. However, the human protein interaction network has more known interactions and a more complex structure. In particular, the calculation/prediction of these interactions is considerably more time consuming. Previous PIPE experiments for *S. cerevisiae* [19,22] and experiments for *C. elegans* reported in [23] showed that PIPE can process each individual protein pair within seconds. However, for human proteins, the picture changes dramatically. The running time for one individual protein pair can fluctuate between less than a second and more than 12 hours. Human proteins have a much more complex structure that appears to lead, in some cases, to a very large number of fragment similarities found by PIPE. When trying to run earlier versions of PIPE on human protein pairs, individual PIPE instances would simply be given static lists of protein pairs to make predictions on. Due to the wide variance of processing time for human protein pairs, some PIPE instances would finish very quickly while, by the end, there may be a single PIPE instance working for hours on a single protein pair while all of the other instances are idle. The imbalance when processing human protein pairs was so great that it resulted in more wasted resources than utilized resources when processing batches of protein pairs. To process a global scan of all human protein pairs, this issue had to be overcome.

The second major issue facing concurrent PIPE instances on a processor is inefficient usage of memory. Typically the number of PIPE processes running on a single machine is set to the number of compute cores on that machine. For example, on a quad-core machine there would typically be four PIPE processes running to utilize the chip fully. If different PIPE processes were left to work completely independently of each other, each process would have to load its own copy of the interaction graph along with all the other PIPE data. For less complex organisms this was not a major issue since the amount of data loaded was relatively small but the complexity of the human proteome translates into significantly more data needed by PIPE. The memory needs for a single PIPE instance for the human proteome increased to such a degree that running as many PIPE instances as compute cores can easily lead to program crashes due to a lack of memory. This would imply that processor cores would be left unused due to memory limitations. To process a global scan of all human protein pairs, this issue had to be overcome.

The basic structure of MP-PIPE is a two-level master/slave and all-slaves model. A single MP-PIPE scheduler process is in charge of managing the main list of protein pairs to be processed as well as reporting the results. The MP-PIPE scheduler distributes work to several MP-PIPE worker processes in packets. Each packet contains a relatively small number of protein pairs. Each MP-PIPE worker executes the PIPE algorithm on protein pairs received from the MP-PIPE scheduler. By giving each worker only a relatively small amount of work at a time we ensure that if a worker does get stuck with abnormally time consuming protein pairs, the other workers will continue to work on their packets and, when they finish, they will request more work from the scheduler process and continue to work. This aspect of the MP-PIPE’s architecture deals with the load imbalance problem by ensuring that all PIPE processes are working as long as there is still work to be done. It should be noted however that if the packet size is too small then the amount of communication between the scheduler and worker processes will negatively impact the running time of the system. It is therefore important to balance the packet size between being too small (too much communication overhead) and too large (too much work imbalance).

To improve the memory efficiency, the second level of MP-PIPES’s architecture uses an “all-slaves” model. Each PIPE worker process consists of a number of parallel threads, called worker threads, among which it distributes the protein pairs to be processed. The worker threads of an MP-PIPE worker are to be executed on a shared memory multi-core processor. The PIPE interaction graph and other necessary PIPE data require considerable amounts of memory. For MP-PIPE, the data stored at an MP-PIPE worker process was re-designed to become a parallel data structure on which all worker threads for that worker can operate concurrently. Much care was taken to implement this as memory efficient as possible so that a single shared copy fits into the main memory of a processor node executing an MP-PIPE worker. This allowed more threads to run simultaneously on a given processor node by reducing the overall memory usage and solved the memory issues discussed. The scheduler/worker part of MP-PIPE was implemented using MPI (Message Passing Interface) and the worker threads within each MP-PIPE worker were implemented in OpenMP (http://openmp.org/).

### Complete scan of the human proteome

MP-PIPE evaluated all possible protein pairs in the human proteome. Most of this work was performed on the large *Victoria Falls* cluster, using the medium cluster to offload some of the harder pairs (i.e. those pairs that took longer than 12 hours). The following represents the human proteome at the time that the MP-PIPE scan was performed.

- Total number of human proteins: 22,513
- Total number of protein pairs to examine: 253,406,328
- Total number of known protein interactions: 41,678
- Total number of proteins with at least one known interacting partner: 9,459
- Total number of proteins with no known interacting partners: 13,054
- Largest number of known interactions partners for a single protein: 265
- Average number of known interactions per protein: 3.70
- Average number of known interactions per protein with at least one interaction: 8.81

The 22,513 human proteins used in this study are made up of the union of Uniprot “confirmed” proteins and proteins involved in physical interactions reported in BioGRID, with no isoforms removed. The 41,678 previously reported interactions were obtained from BioGRID, which is an amalgamation of several experimental studies. The human proteome has almost seven times more known interactions than the *C. elegans* proteome, and the average human protein has more than double the known interactions than a *C. elegans* protein. Coupled with the fact that the human proteins are, on average, longer than the *C. elegans* proteins, this significantly increases the complexity of scanning the entire human proteome. Furthermore, as outlined above, the running time for one individual protein pair can fluctuate between less than a second and more than 12 hours. This creates an additional load-balancing problem as discussed above. In fact, some individual protein pairs required six days of computation.

On the Victoria Falls cluster, 50 nodes were used, each with their own MP-PIPE worker process running 256 threads. This implies 12,800 parallel computational threads running on 6,400 hardware-supported threads. The number of threads per node was scaled down from 512 threads due to the fact that each individual thread needed significantly more memory than in our tests. The Victoria Falls cluster was used to process the vast majority of protein pairs. The results presented in this paper were obtained through three months of exclusive 24/7 use of the large Victoria Falls cluster. If one of its worker threads got stuck with a protein pair that was running more than 12 hours, that protein pair was off-loaded to the medium cluster since its individual cores are more powerful than a single Victoria Falls thread.

### Hubs and betweenness centrality

Protein interactions can be represented as an interaction network, where the proteins are interactors (nodes) and connections (interactions) are shown as edges. The number of edges incident to one node is called the degree of that node. Hubs are high degree nodes that interact with many other proteins (nodes) through various pathways [79]. To find the hubs in the human predicted interaction graph, the proteins were sorted by their degree in the network. Betweenness centrality is an important global property of networks. The betweenness centrality of a node *v* is the ratio of the number of shortest paths between a pair of nodes *a* and *b* on which *v* lies and the total number of shortest paths between *a* and *b*, summed over all possible pairs of nodes. These measures were used to identify proteins of interest. The betweenness centrality of a protein *v* is defined as $(v) = {\displaystyle {\sum}_{a\ne v\ne b}\frac{\sigma_{ab}(v)}{\sigma_{ab}}}$, where *σ*_*ab*_(*v*) = # shortest paths from *a* to *b* through *v*, *σ*_*ab*_ = # shortest paths between *a* and *b*.

### Versatile affinity-tagging, purification, and protein identification

The full length, sequence verified, non-mutated, Gateway-compatible cDNA entry clones for the human chromatin-related proteins (CBX1, RNF2, H2AFX, and RBBP4) were obtained from Harvard PlasmidID. The HEK293 and HEK293T cells were cultured in Dulbecco’s modified Eagle’s medium with 10% fetal bovine serum and antibiotics essentially as previously described [28,80]. The sequence**-**verified clones were cloned into the lentiviral expression vector essentially as previously described [28,80]. The lentivirus-encoded tagged ORFs were transduced into HEK293 cells, and the stably expressed cells were subsequently selected with puromycin at a concentration of 2 μg/ml for a minimum of 48 hours and expanded, essentially as described previously [28,80]. The expression of the tag in stable cells was subsequently confirmed by Western blotting using anti-FLAG antibody against the 3X FLAG epitope. Affinity purification and sample processing for protein identification by mass spectrometry was performed essentially as previously described [28,80]. The high**-**confidence matches of the resulting MS/MS spectra was mapped to the reference human protein sequences using the SEQUEST database search engine with match quality evaluated using the STATQUEST algorithm [81]. The identified co-purifying proteins were filtered out at a confidence threshold at 90% with two or more peptides. Since each tagged samples were independently affinity purified two times to rule out the non-specific binding proteins, we averaged the peptide counts over the replicates. Moreover, the co-purifying proteins that were identified at 90% cut-off with one single peptide and the most common background contaminants or proteins that bound to the unrelated VA-tagged GFP samples in our replicate purifications were filtered out to eliminate the noise from the dataset.

The comparison of LGTS-MS results with the MP-PIPE predicted and previously reported (known) interactions was done by calculating the precision and recall on the basis of LGTS-MS results representing real interactions. To do this we filtered the MP-PIPE predictions and known interactions to contain detectable proteins (the set of all proteins seen in any of the LGTS-MS experiments performed here). Reachable proteins were defined as those proteins that interact directly with the bait or indirectly through one or two intermediaries within the sets of filtered interactions.

### Yeast growth condition

Standard rich (YPD) and synthetic complete (SC) media were used as a growth media [72]. To investigate translation inhibitory drugs (antibiotics) on growth rate of yeast deletion mutants, streptomycin (40 mg/ml) was added to SC media and cycloheximide (60 ng/ml) was added to YPD media [72].

### Drug sensitivity test

Yeast cells were grown in liquid YPD or SC media to mid-log phase (48 hours) and diluted to a concentration of 10^−2^ to 10^−5^ cells/20 μl. From each dilution, 20 μl was spotted onto solid media containing translation inhibitory drugs. Media with no antibiotics was used as a control. All yeast cells were incubated at 30°C for 1–2 days [82].

### Protein expression assay

Translation fidelity was measured using plasmids pUKC817, pUKC818 and pUKC819, which carry premature stop codons UAA, UGA and UAG in a *β -galactosidase* expression cassette [72,83]. Translation efficiency was assessed using plasmid p416 (containing Gal-inducable LacZ expression cassette) as described by [72,84]. *β-galactosidase* assay was performed using ONPG, O-nitrophenil-α-D-galactopyranoside, as descibed by [84-86].

### qRT-PCR

Total RNA was extracted using RNeasy Mini Kit (QIAGEN) according to manufacturer's instruction. To synthesize cDNA, 33 μl of RNA in combination with 3 μl poly T primer (random hexamer) was incubated for five min at 70°C, and then cooled on ice for five min. 6 μl RT-buffer, 15 μl dNTPs, and 3 μl RNaseI were added to the mixture and incubated for five min at 37°C. 3 μl RT enzyme was added to the mixture and incubated for an additional 1 hour at 42°C followed by aa 10 min incubation at 70°C. qPCR amplification and detection was performed on a Rotor Gene 3000 from Corbett Research. A final mixture of 2.4 μl H_2_O, 2.4 μl 10X PCR buffer, 1,25 μl dNTPs(4 mM), 1.25 μl MgCl_2_, 2 μl SYBR Green (1/4000), 7.6 μl primer mix (1 mM) and 3 μl Taq was used in addition to 5 μl template (cDNA). Thermocycler conditions were set to the following: 50°C for two min, 95°C for 10 min, 40 cycles of 95°C for 30s-60°C for 30s-72°C for 30s and a final 72°C for ten min [87,88]. The primers used in the qRT-PCR were designed based on the sequences for LacZ (F: TTGAAAATGGTCTGCT GCTG, R: TATTGGCTTCATCCACCACA) and PGK-1 (F: CAGACCATTCTTGGCCATT, R: CGAAGATGGAGTCACCGATT). PGK-1 (phosphoglycerate kinase) was selected as the positive control due to being one of the most highly expressed genes in yeast, producing up to 5% of total mRNA content [89].

### Prediction and analysis of candidate biomarkers for seasonal allergic rhinitis

40 patients with SAR were included in the study. SAR and symptom scores were defined as previously described [70,90]. Their median (range) age was 28 (18–58) and 19 were women. The mean ± SEM symptom score of the 40 patients after treatment decreased from 15.7 ± 1.0 to 4.3 ± 0.6 (*P* < 0.0000001). The study was approved by the Ethics Committee of the Medical Faculty of the University of Gothenburg. Written informed consent and questionnaire data sheets were obtained from all patients.

Proteins from the acute phase response pathway, complement signaling pathway, and glucocorticoids receptor pathway were extracted from the Ingenuity pathway analysis (IPA) software. Interactors to these proteins were predicted using PIPE. We included secreted, membrane and cytoplasmic proteins, but excluded nuclear proteins. We prioritized candidate biomarkers based on their number of known and predicted interactions. Next, we focused on proteins with a high number of predicted interactions.

Proteins were examined by ELISA in nasal fluids from 40 patients with SAR before and after GC treatment. V-akt murine thymoma viral oncogene homolog 2 (Akt2) was with an ELISA kit from R&D Systems Inc. (Minneapolis, MN, USA). Proline-rich protein BstNI subfamily 1 (PRB1), proline-rich protein BstNI subfamily 2 (PRB2), v-yes-1 Yamaguchi sarcoma viral related oncogene homolog (LYN) and stratifin (SFN) were analyzed with ELISA kits from Uscnlife Life Sciences and Technology (Wuhan, China). All experiments were performed according to the manufacturer’s protocol. The Wilcoxon matched pairs signed ranks test was performed to compare two paired groups. A P-value less than 0.05 was considered significant.

### Graph algorithms for assigning protein complexes

To decompose the predicted protein pairs into putative complexes, we applied a novel algorithm that combines pre-existing graph-theoretic tools with hierarchical clustering concepts. The algorithm has three independent stages: the initialization stage, which consists of generating an initial set of clusters, the merge stage, which determines which two clusters to merge next, if any, and the glom stage, which evaluates vertices for inclusion into a cluster. The initialization stage is run once, after which the merge and glom stages run alternately until either the desired number of clusters is reached or until neither stage results in a change to any cluster.

Since initialization is an independent step, any initial clustering may be used. It is not required that the initial clustering be overlapping, although stages two and three may grow the clusters so that the end result is overlapping. We chose to use the set of all maximal cliques as the initial clustering. A clique is a set of vertices with all edges present; a clique is maximal if no vertex can be added to it to form a larger clique. The set of maximal cliques forms a natural overlapping clustering of a graph with the most rigid requirements, namely that all edges be present within each cluster. Real-world graphs often have many small and medium sized maximal cliques, and the protein prediction graph is no exception. These clusters are then allowed to merge and grow in stages two and three, gradually relaxing the stringency until the desired number of clusters is reached. To enumerate all maximal cliques, we used the well-known algorithm of Bron and Kerbosch described in [91] with bitwise improvements from [92].

In the merge stage, the overlap of all clusters is evaluated and the two clusters with the highest overlap proportion are merged. If no two clusters overlap by a proportion greater than a parameter *m*, then no clusters are merged.

In the glom stage, every vertex not already belonging to a particular cluster is considered for inclusion into a cluster in similar fashion to the paraclique algorithm described in [76]. Those vertices with connectivity proportion greater than *g*, the glom factor, are added to the cluster. The first time through the glom stage, every cluster is considered. Subsequent glom stages only consider the cluster newly created by the merge stage, as all other clusters having already been considered.

In practice, calculating all pairwise overlaps to find the highest degree of overlap can make the merge stage computationally prohibitive. A small change, however, yields a good approximation version that can be run until the number of clusters is reduced to the point where the exact version can take over. Rather than merging the clusters with the highest overlap, the approximation version merges the first two clusters encountered with overlap at least *a*, the approximation parameter. For the protein prediction graph, which was initialized with more than 100,000 maximal cliques, we ran the approximation version until the number of clusters reached 20,000, at which point we switched to the exact version. Ultimately, a list of 8,739 paracliques were identified and characterized through a statistical analysis of the GO annotations of each member protein.

## Additional files

Additional file 1:
**Precision-Recall curve for H. sapiens PPIs.** The curves presents the Recall (True Positive Rate) against Precision including homologs (dashed blue line) and with homologs removed (dotted pink line). Additional file 2:
**List of** ***H. sapiens*** **interactions predicted in this study.** Each prediction is accompanied by a score, if the interaction was previously reported (known) or novel, if both proteins are in the same component, have the same function or involved in the same process (GO ontology) as well as if they share a third party interaction. Additional file 3:
**List of co-purifying proteins that interact with the tagged chromatin-related bait proteins.**
Additional file 4:
**Number interacting pairs where both proteins have the same function (A) or are involved in the same cellular process (B) for previously reported data compared with those reported in this study only.**
Additional file 5:
**List of interactions for fibroblast growth factors (FGFs) and cyclin-dependent kinases (CDKs) and FGF regulators and CDK inhibitor/activators.** *are interactors that have not been previously reported. Additional file 6:
**List of hub proteins in** ***H. sapiens*** **ranked by the number of neighbors for each protein.**
Additional file 7:
**List of betweenness centrality proteins in** ***H. sapiens*** **ranked by highest betweenness centrality measure.**
Additional file 8:
**List of protein mutations associated with resistance to breast cancer therapeutics doxorubicin and Trastuzumab.**
Additional file 9:
**List of proteins from the acute phase response pathway, complement pathway and glucocorticoids receptor pathway.**
Additional file 10:
**List of paracliques identified in this study including the member proteins for the paraclique, interactions in the paraclique as well as GO tags for molecular function and biological process shared by the members of the paraclique with P-value 1E-06.**
Additional file 11:
**Schematic representation of the interactions for three putative complexes, paracliques, 1359 (A) 1409 (B) and 2164 (C).** A high degree of interconnectivity is observed for these complexes. Red and green edges represent previously reported (known), and novel interactions, respectively.
