# Supplementary figures and images for: Efficient prediction of human protein-protein interactions at a global scale
Source: BMC Bioinformatics. 2014 Dec 10;15(1):383. doi: 10.1186/s12859-014-0383-1 (PMC4272565; doi:10.1186/s12859-014-0383-1)

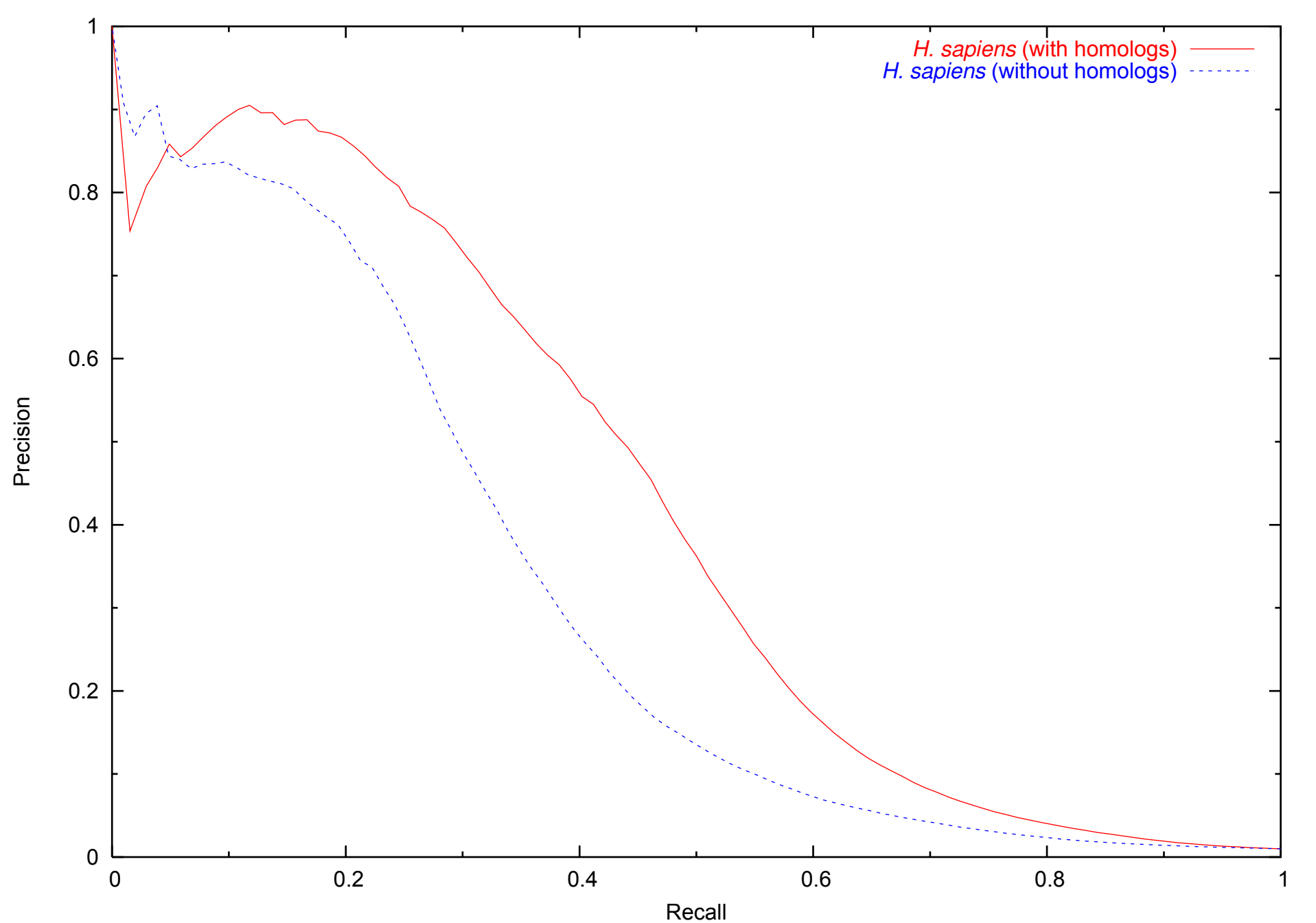

Supplement: Additional file 1: — Precision-Recall curve for H. sapiens PPIs. The curves presents the Recall (True Positive Rate) against Precision including homologs (dashed blue line) and with homologs removed (dotted pink line). [file 12859_2014_383_MOESM1_ESM.pdf]

**A**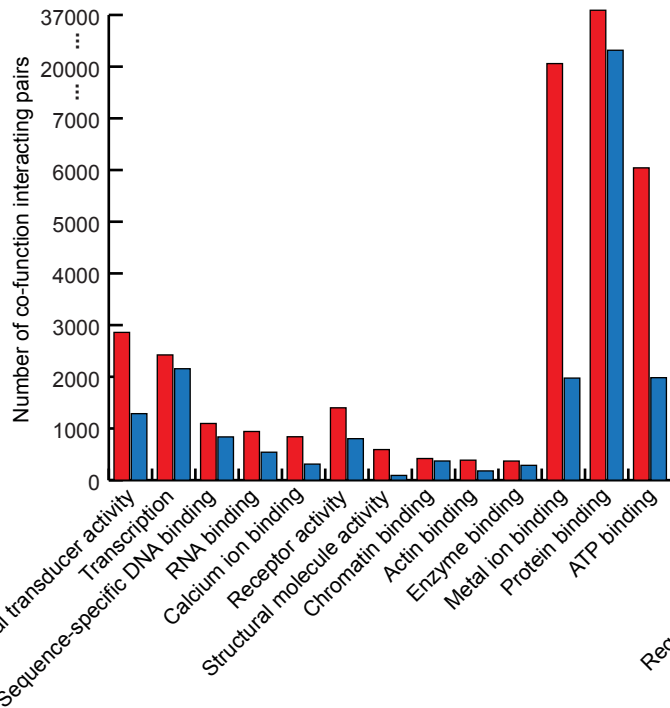**B**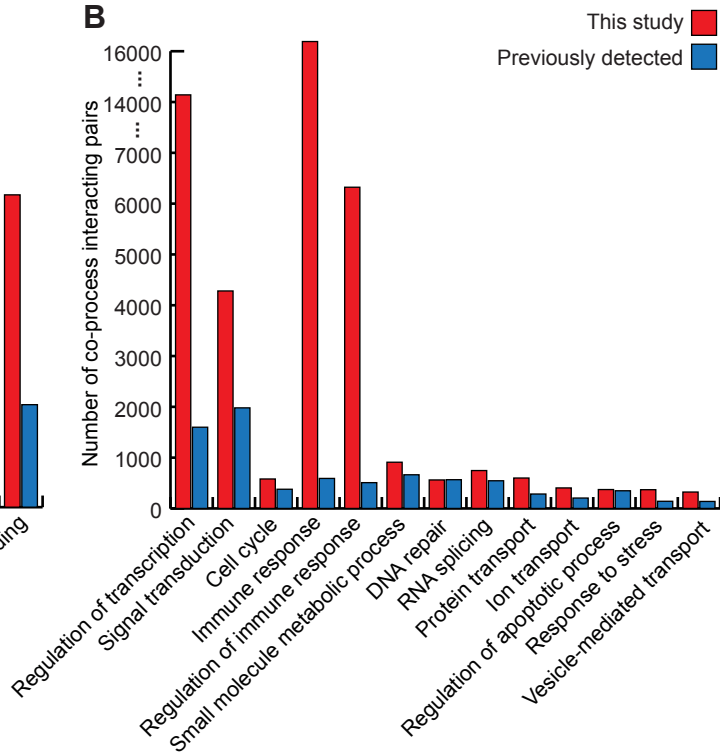

Supplement: Additional file 4: — Number interacting pairs where both proteins have the same function (A) or are involved in the same cellular process (B) for previously reported data compared with those reported in this study only. [file 12859_2014_383_MOESM4_ESM.pdf]

..... Previously detected  
— This study

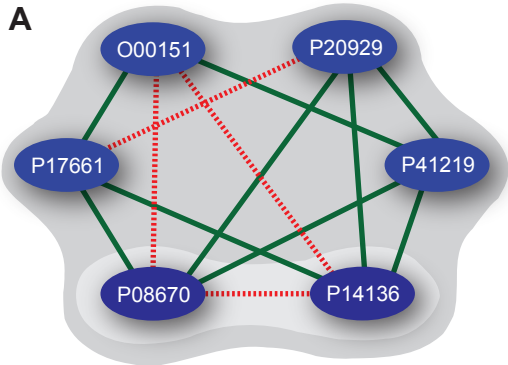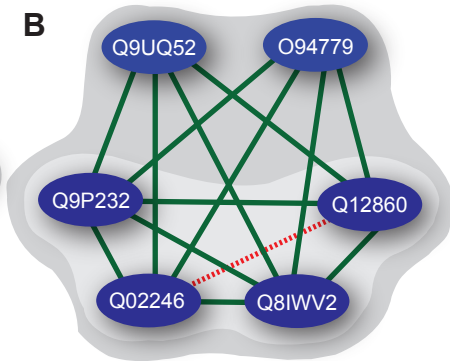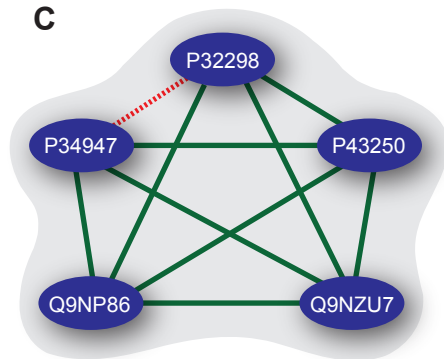

Supplement: Additional file 11: — Schematic representation of the interactions for three putative complexes, paracliques, 1359 (A) 1409 (B) and 2164 (C). A high degree of interconnectivity is observed for these complexes. Red and green edges represent previously reported (known), and novel interactions, respectively. [file 12859_2014_383_MOESM11_ESM.pdf]
