# Supplementary material for: Efficient prediction of human protein-protein interactions at a global scale
Source: BMC Bioinformatics. 2014 Dec 10;15(1):383. doi: 10.1186/s12859-014-0383-1 (PMC4272565; doi:10.1186/s12859-014-0383-1)
Supplement: Additional file 2: — List of H. sapiens interactions predicted in this study. Each prediction is accompanied by a score, if the interaction was previously reported (known) or novel, if both proteins are in the same component, have the same function or involved in the same process (GO ontology) as well as if they share a third party interaction. [file 12859_2014_383_MOESM2_ESM.pdf]

Protein A	Protein B	Score	Novel / Known	Same component	Same function	Same process	Third Party Interaction
Q5VST9	Q8WZ42	0.99999999	Known	YES	YES	YES	YES
P20929	Q8WZ42	0.99999998	Known	YES	YES	NO	YES
P21333	Q8WZ42	0.99999996	Known	YES	YES	NO	NO
P35579	Q8WZ42	0.99999994	Known	YES	YES	NO	YES
P16157	Q8WZ42	0.99999994	Known	YES	NO	NO	YES
P52179	Q8WZ42	0.99999993	Known	NO	YES	NO	YES
P54296	Q8WZ42	0.99999992	Known	NO	YES	NO	YES
Q86TC9	Q8WZ42	0.99999991	Known	YES	NO	NO	YES
Q14896	Q8WZ42	0.99999991	Known	NO	YES	NO	YES
Q00872	Q8WZ42	0.9999999	Known	NO	YES	NO	NO
Q14596	Q8WZ42	0.99999988	Known	NO	NO	NO	NO
Q01484	Q5VST9	0.99999987	Known	YES	YES	NO	NO
P35609	Q8WZ42	0.99999987	Known	YES	YES	NO	YES
P12814	Q8WZ42	0.99999987	Known	YES	YES	NO	NO
P20807	Q8WZ42	0.99999985	Known	YES	YES	NO	NO
P21817	Q92736	0.99999984	Known	YES	YES	YES	YES
Q15413	Q92736	0.99999983	Known	YES	YES	YES	NO
P08519	P98164	0.99999981	Known	NO	NO	YES	YES
P04114	P98164	0.99999981	Known	YES	NO	NO	NO
O60494	P98164	0.99999976	Known	YES	YES	YES	YES
O94833	Q9NYQ7	0.99999976	Known	NO	YES	NO	NO
Q5VST9	Q9Y623	0.99999974	Novel	YES	YES	NO	NO
Q5VST9	Q9UKX2	0.99999974	Known	YES	YES	NO	NO
P16157	Q5VST9	0.99999973	Known	YES	NO	NO	YES
Q14643	Q92736	0.9999997	Known	YES	YES	YES	YES
P25391	P98160	0.9999997	Known	YES	NO	NO	YES
Q13439	Q96PK2	0.99999969	Known	YES	NO	NO	NO
P01266	P98164	0.99999969	Known	NO	NO	NO	NO
O95613	Q99996	0.99999969	Known	YES	NO	NO	YES
P78527	Q13315	0.99999968	Known	YES	YES	YES	YES
P35555	P98160	0.99999968	Known	YES	YES	NO	YES
P11532	P46939	0.99999968	Known	YES	YES	YES	YES
Q8WZ42	Q9BYX7	0.99999967	Novel	YES	YES	NO	NO
Q96L91	Q9Y4A5	0.99999967	Known	YES	NO	YES	YES
P62736	Q8WZ42	0.99999967	Novel	YES	YES	NO	NO
P68032	Q8WZ42	0.99999967	Novel	YES	YES	YES	NO
P63267	Q8WZ42	0.99999967	Novel	YES	YES	NO	NO
P63261	Q8WZ42	0.99999967	Novel	YES	YES	YES	NO
Q562R1	Q8WZ42	0.99999967	Novel	YES	YES	NO	NO

P60709	Q8WZ42	0.99999967	Novel	YES	YES	NO	NO
P68133	Q8WZ42	0.99999967	Known	YES	YES	YES	NO
Q8WZ42	Q9GZV1	0.99999966	Known	NO	YES	NO	YES
Q09472	Q15751	0.99999966	Known	YES	NO	NO	NO
P98164	Q9Y490	0.99999966	Known	NO	YES	NO	NO
Q8WZ42	Q969Q1	0.99999965	Known	YES	NO	NO	NO
Q96PK2	Q9Y4D7	0.99999965	Known	NO	NO	NO	NO
Q13813	Q15149	0.99999965	Known	YES	YES	NO	YES
Q13023	Q92736	0.99999965	Known	YES	NO	NO	YES
Q12955	Q9H254	0.99999964	Known	YES	YES	NO	NO
P02549	Q15149	0.99999964	Known	YES	NO	NO	NO
Q13936	Q92736	0.99999963	Known	YES	YES	YES	YES
P98161	Q9Y490	0.99999963	Known	NO	YES	NO	YES
P02751	P08519	0.99999963	Known	YES	NO	NO	NO
O15230	Q02388	0.99999963	Known	YES	NO	NO	NO
P00451	Q07954	0.99999962	Known	YES	YES	NO	YES
Q15327	Q8WZ42	0.99999961	Known	YES	NO	NO	YES
P51587	Q13315	0.99999961	Known	YES	NO	YES	YES
P02751	P98161	0.99999961	Known	NO	NO	NO	YES
P02751	P98160	0.99999961	Known	YES	NO	YES	YES
Q96PK2	Q9P2S2	0.9999996	Known	NO	YES	NO	NO
Q14686	Q8NEZ4	0.9999996	Known	YES	YES	NO	YES
Q86SG2	Q8WZ42	0.99999959	Known	YES	NO	NO	YES
Q03164	Q92793	0.99999958	Known	YES	YES	YES	YES
P13611	P35555	0.99999958	Known	YES	YES	NO	YES
P21817	Q13698	0.99999957	Known	YES	YES	YES	NO
O94833	P16144	0.99999957	Known	NO	YES	YES	YES
Q96T58	Q9Y618	0.99999956	Known	YES	YES	NO	YES
Q01082	Q01484	0.99999956	Known	YES	NO	NO	NO
P98164	Q9NY46	0.99999956	Known	YES	NO	NO	NO
Q07954	Q9NY46	0.99999955	Known	NO	NO	NO	NO
Q14192	Q8WZ42	0.99999955	Known	YES	YES	NO	NO
P51587	Q13535	0.99999955	Known	YES	YES	YES	YES
P25391	Q02388	0.99999955	Known	YES	NO	NO	NO
P21333	P51587	0.99999955	Known	YES	NO	NO	YES
O75376	Q96T58	0.99999955	Known	NO	YES	YES	YES
Q96SN8	Q9P2P6	0.99999954	Known	NO	NO	NO	NO
Q96PK2	Q9ULB1	0.99999954	Known	NO	YES	NO	NO
Q12955	Q99250	0.99999954	Known	NO	NO	NO	NO
P20929	Q86TC9	0.99999954	Known	YES	YES	NO	YES
O75445	Q01955	0.99999953	Known	YES	NO	YES	NO

P49454	Q02224	0.99999953	Known	YES	NO	YES	NO
P11137	Q15149	0.99999953	Known	YES	NO	NO	YES
Q9H254	Q9NYQ7	0.99999952	Known	NO	YES	NO	NO
P78527	Q14686	0.99999952	Known	YES	YES	YES	YES
P35555	P35556	0.99999952	Known	YES	YES	NO	YES
P16144	Q15149	0.99999952	Known	YES	NO	NO	YES
O43157	Q03164	0.99999952	Known	NO	NO	NO	NO
Q93074	Q99698	0.99999951	Known	NO	NO	NO	NO
P51610	Q03164	0.9999995	Known	YES	YES	YES	YES
P42858	Q9BYW2	0.9999995	Known	YES	YES	NO	NO
Q13315	Q13535	0.9999995	Known	YES	YES	YES	YES
O75962	P21333	0.9999995	Known	YES	NO	NO	YES
O75376	Q6ZRS2	0.99999949	Known	NO	NO	YES	NO
O15020	Q9NYQ7	0.99999949	Known	NO	NO	NO	NO
Q96PK2	Q9UHC6	0.99999948	Known	NO	NO	NO	NO
Q96PK2	Q9C0A0	0.99999948	Known	NO	NO	NO	NO
Q92823	Q96PK2	0.99999947	Known	NO	YES	NO	NO
P38398	P78527	0.99999947	Known	YES	NO	YES	YES
P42858	Q92793	0.99999947	Known	YES	NO	NO	YES
O94833	Q9UMD9	0.99999947	Known	NO	NO	NO	YES
O75376	P42858	0.99999947	Known	NO	YES	NO	YES
O15146	Q8NF91	0.99999946	Known	NO	NO	NO	NO
Q6VMQ6	Q96PK2	0.99999946	Known	YES	YES	NO	NO
P01024	Q07954	0.99999946	Known	NO	NO	NO	YES
Q8NF91	Q9NRI5	0.99999945	Known	YES	NO	NO	NO
Q14573	Q14643	0.99999945	Known	YES	YES	YES	YES
Q13402	Q9H251	0.99999945	Known	NO	NO	YES	YES
P12270	P42858	0.99999945	Known	YES	YES	YES	NO
Q5THJ4	Q8WXG6	0.99999944	Known	NO	NO	NO	NO
P38398	Q9Y4A5	0.99999944	Known	YES	NO	NO	YES
O95359	Q92793	0.99999944	Known	YES	NO	NO	YES
O94833	Q96RT1	0.99999944	Known	YES	YES	YES	YES
P25054	Q12923	0.99999943	Known	YES	NO	NO	YES
O95359	Q09472	0.99999943	Known	YES	NO	NO	YES
Q12955	Q13009	0.99999942	Known	NO	YES	NO	NO
Q15149	Q9UMD9	0.99999942	Known	NO	NO	NO	YES
P02751	Q02388	0.99999942	Known	YES	NO	YES	YES
P98164	Q96QZ7	0.99999941	Known	NO	NO	NO	YES
P46821	P78559	0.99999941	Known	YES	YES	NO	YES
O75369	P21333	0.99999941	Known	YES	NO	NO	YES
O95613	Q15154	0.9999994	Known	YES	NO	YES	NO

Q9UPA5	Q9UPW8	0.99999939	Known	YES	YES	NO	YES
Q86UR5	Q9UPA5	0.99999939	Known	YES	YES	NO	YES
P00451	P04275	0.99999939	Known	YES	NO	NO	YES
P01023	Q07954	0.99999939	Known	NO	NO	NO	YES
O94833	Q14203	0.99999938	Known	YES	YES	NO	NO
Q14669	Q9NYQ7	0.99999938	Known	NO	NO	NO	NO
Q96PK2	Q9C0B7	0.99999937	Known	NO	NO	NO	NO
P98164	Q5JY77	0.99999937	Known	NO	NO	NO	NO
Q8WZ42	Q9NVP2	0.99999936	Known	YES	NO	NO	NO
Q13315	Q14676	0.99999936	Known	YES	NO	YES	YES
P38398	P51587	0.99999936	Known	YES	NO	YES	YES
P02458	P98161	0.99999936	Known	NO	NO	NO	YES
O14827	P58107	0.99999935	Known	YES	YES	NO	NO
P42858	Q12873	0.99999935	Known	YES	YES	NO	YES
P02452	P98161	0.99999935	Known	NO	NO	NO	YES
P25054	P56715	0.99999934	Known	NO	NO	NO	NO
P46531	Q09472	0.99999934	Known	YES	YES	YES	YES
O75376	Q9Y618	0.99999934	Known	YES	YES	YES	YES
P07384	Q8NF91	0.99999934	Known	YES	NO	NO	YES
O43707	P20929	0.99999933	Known	YES	NO	NO	NO
A2VEC9	P54259	0.99999933	Known	NO	NO	NO	NO
Q12888	Q13315	0.99999933	Known	YES	NO	YES	YES
P98160	Q14112	0.99999933	Known	YES	NO	YES	YES
P00533	P58107	0.99999933	Known	YES	NO	NO	YES
O15230	P11047	0.99999932	Known	YES	NO	YES	YES
Q09472	Q92793	0.99999931	Known	YES	YES	YES	YES
P78527	Q14191	0.99999931	Known	YES	YES	YES	YES
P46531	Q9UM47	0.99999931	Known	YES	YES	YES	YES
O15020	P02549	0.9999993	Known	YES	YES	YES	YES
P15924	Q92817	0.9999993	Known	YES	YES	YES	YES
O75962	P10586	0.9999993	Known	NO	NO	YES	NO
P38398	Q13315	0.99999929	Known	YES	NO	YES	YES
P02545	Q8NF91	0.99999929	Known	YES	NO	NO	YES
P00533	Q15149	0.99999928	Known	YES	NO	NO	YES
P98160	Q6VMQ6	0.99999927	Known	NO	YES	NO	NO
O95477	Q92793	0.99999926	Known	NO	NO	NO	YES
P54259	P98164	0.99999926	Known	NO	NO	NO	YES
Q03164	Q99683	0.99999925	Known	NO	YES	YES	NO
P98161	Q6VMQ6	0.99999925	Known	NO	YES	NO	NO
Q02817	Q9Y4G2	0.99999925	Known	NO	NO	NO	NO
P58107	Q15714	0.99999925	Known	YES	YES	NO	YES

P02511	Q8WZ42	0.99999925	Known	YES	NO	NO	NO
P14543	P98160	0.99999925	Known	YES	YES	YES	YES
P07996	P98164	0.99999925	Known	NO	YES	NO	NO
Q13813	Q9Y566	0.99999924	Known	YES	NO	NO	YES
Q6UYC3	Q8NF91	0.99999923	Novel	NO	NO	NO	NO
P23229	Q15149	0.99999923	Known	YES	NO	NO	YES
P02751	P24821	0.99999923	Known	YES	NO	YES	YES
P07996	Q07954	0.99999923	Known	YES	YES	NO	YES
P11277	Q13813	0.99999923	Known	YES	YES	YES	YES
P54259	P98160	0.99999922	Known	NO	NO	NO	YES
O15273	Q8WZ42	0.99999921	Known	YES	YES	YES	YES
P98095	P98160	0.99999921	Known	YES	NO	NO	YES
P17301	P98160	0.99999921	Known	NO	NO	YES	YES
P02549	P11277	0.99999921	Known	YES	YES	YES	YES
Q99698	Q9HC77	0.9999992	Known	YES	NO	NO	YES
Q01484	Q92823	0.9999992	Known	YES	YES	NO	NO
Q13535	Q14839	0.9999992	Known	YES	YES	NO	YES
Q14676	Q92793	0.9999992	Known	YES	NO	NO	YES
P01024	P25391	0.9999992	Known	YES	YES	NO	NO
Q14686	Q92793	0.99999919	Known	YES	NO	NO	YES
Q09472	Q14676	0.99999919	Known	YES	YES	YES	YES
P51587	Q9BXW9	0.99999919	Known	YES	YES	YES	YES
P16157	Q14573	0.99999919	Known	YES	NO	NO	NO
Q96K83	Q99698	0.99999918	Known	NO	NO	NO	NO
Q12873	Q5UIP0	0.99999918	Known	YES	NO	NO	YES
Q09472	Q14686	0.99999918	Known	YES	NO	YES	YES
P11532	Q9ULU8	0.99999918	Known	YES	YES	NO	NO
O15083	Q9Y6V0	0.99999917	Known	YES	NO	NO	NO
P38398	Q13535	0.99999917	Known	YES	NO	YES	YES
P32004	Q01484	0.99999917	Known	YES	YES	NO	NO
P02462	Q02388	0.99999917	Known	YES	NO	NO	YES
O95477	Q9Y566	0.99999917	Known	YES	NO	NO	NO
Q96RY7	Q9NYQ7	0.99999916	Known	NO	NO	NO	NO
P18206	P98161	0.99999916	Known	NO	NO	NO	YES
O60674	P98161	0.99999916	Known	YES	NO	NO	YES
Q8IWT3	Q9ULL4	0.99999915	Known	NO	NO	NO	NO
Q71F56	Q9UHV7	0.99999915	Known	YES	YES	YES	YES
Q6P0Q8	Q92616	0.99999915	Known	YES	YES	NO	NO

… (continued)
