# Supplementary material for: Efficient prediction of human protein-protein interactions at a global scale
Source: BMC Bioinformatics. 2014 Dec 10;15(1):383. doi: 10.1186/s12859-014-0383-1 (PMC4272565; doi:10.1186/s12859-014-0383-1)
Supplement: Additional file 3: — List of co-purifying proteins that interact with the tagged chromatin-related bait proteins. [file 12859_2014_383_MOESM3_ESM.pdf]

Gene	Swissprot Name	UniprotID	DESCRIPTION	CBX1		RNF2		RBBP4		H2AFX	
				Number of peptides	Probability	Number of peptides	Probability	Number of peptides	Probability	Number of peptides	Probability
AAGAB	AAGAB_HUMAN	Q6PD74	Alpha- and gamma-adaptin-binding protein p34	2	99.58	0	0	0	0	0	0
AAMDC	AAMDC_HUMAN	Q9H7C9	Mth938 domain-containing protein	16	99.58	8	99.58	12	99.58	16	99.58
AAMP	AAMP_HUMAN	Q13685	Angio-associated migratory cell protein	7	99.58	0	0	2	99.58	0	0
AARD	AARD_HUMAN	Q4LEZ3	Alanine and arginine-rich domain-containing protein	0	0	0	0	0	0	2	99.58
ABCE1	ABCE1_HUMAN	P61221	ATP-binding cassette sub-family E member 1	2	99.58	0	0	0	0	0	0
ACLY	ACLY_HUMAN	P53396	ATP-citrate synthase	3	99.58	2	99.58	8	99.58	0	0
ACOT7	BACH_HUMAN	O00154	Cytosolic acyl coenzyme A thioester hydrolase	0	0	0	0	2	86.74	0	0
ACTG1	ACTG_HUMAN	P63261	Actin, cytoplasmic 2;Actin, cytoplasmic 2, N-terminally proces	4	99.58	5	99.58	9	99.58	5	99.58
ACTL6A	ACL6A_HUMAN	O96019	Actin-like protein 6A	2	99.58	0	0	0	0	0	0
ACTR1A	ACTZ_HUMAN	P61163	Alpha-centractin	8	99.58	4	99.58	4	99.58	2	99.58
ACTR1B	ACTY_HUMAN	P42025	Beta-centractin	3	99.58	3	99.58	2	99.58	2	99.58
ADRM1	ADRM1_HUMAN	Q16186	Proteasomal ubiquitin receptor ADRM1	0	0	0	0	0	0	2	99.58
ADSL	PUR8_HUMAN	P30566	Adenylosuccinate lyase	2	99.58	2	99.58	2	99.58	0	0
AIP	AIP_HUMAN	O00170	Aurora kinase A-interacting protein	7	99.58	0	0	0	0	2	99.58
AKR1A1	AK1A1_HUMAN	P14550	Alcohol dehydrogenase [NADP(+)]	2	99.58	2	99.58	2	99.58	0	0
AKR1B1	ALDR_HUMAN	P15121	Aldose reductase	2	99.58	2	99.58	4	99.58	0	0
ALB	ALBU_HUMAN	P02768	Fas-binding factor 1	3	99.58	2	99.58	2	99.58	0	0
ALDH7A1	AL7A1_HUMAN	P49419	Alpha-aminoadipic semialdehyde dehydrogenase	7	99.58	0	0	2	99.58	0	0
AMOT	AMOT_HUMAN	Q4VCS5	Angiomotin	16	99.58	3	99.58	9	99.58	12	99.58
AMOTL1	AMOL1_HUMAN	Q8IY63	Angiomotin-like protein 1	0	0	0	0	0	0	2	99.58
ANKFY1	ANFY1_HUMAN	Q9P2R3	Ankyrin repeat and FYVE domain-containing protein 1	3	99.58	0	0	0	0	4	99.58
ANP32A	AN32A_HUMAN	P39687	Acidic leucine-rich nuclear phosphoprotein 32 family member	0	0	0	0	4	99.58	0	0
APEX1	APEX1_HUMAN	P27695	DNA-(apurinic or apyrimidinic site) lyase;DNA-(apurinic or apy	0	0	2	99.58	0	0	0	0
API5	API5_HUMAN	Q9BZZ5	Apoptosis inhibitor 5	24	99.58	15	99.58	8	99.58	15	99.58
APPL1	DP13A_HUMAN	Q9UKG1	DCC-interacting protein 13-alpha	4	99.58	0	0	0	0	0	0
APRT	APT_HUMAN	P07741	Adenine phosphoribosyltransferase	0	0	2	99.58	0	0	0	0
ARF1	ARF1_HUMAN	P84077	ADP-ribosylation factor 1	14	99.58	0	0	0	0	0	0
ARF4	ARF4_HUMAN	P18085	ADP-ribosylation factor 4	8	99.58	6	99.58	11	99.58	7	99.58
ARF5	ARF5_HUMAN	P84085	ADP-ribosylation factor 5	4	99.58	2	99.58	4	99.58	3	99.58
ARF6	ARF6_HUMAN	P62330	ADP-ribosylation factor 6	6	99.58	0	0	0	0	4	99.58
ARFGAP1	ARFG1_HUMAN	Q8N6T3	ADP-ribosylation factor GTPase-activating protein 3	0	0	0	0	2	99.58	0	0
ARFGAP3	ARFG3_HUMAN	Q9NP61	ADP-ribosylation factor GTPase-activating protein 3	0	0	2	99.58	0	0	3	99.58
ARHGAP1	RHG01_HUMAN	Q07960	Rho GTPase-activating protein 1	11	99.58	0	0	0	0	2	99.58
ARHGAP17	RHG17_HUMAN	Q68EM7	Rho GTPase-activating protein 17	3	99.58	2	99.58	0	0	2	99.58
ARHGAP27	RHG27_HUMAN	Q6ZUM4	Rho GTPase-activating protein 27	0	0	2	86.03	0	0	0	0
ARHGAP44	RHG44_HUMAN	Q17R89	Rho GTPase-activating protein 44	0	0	0	0	0	0	2	99.58
ARHGAP9	RHG09_HUMAN	Q9BRR9	Rho GTPase-activating protein 9	0	0	2	96.39	0	0	0	0
ARHGEF2	ARHG2_HUMAN	Q92974	Rho guanine nucleotide exchange factor 2	8	99.58	0	0	0	0	0	0
ARMC6	ARMC6_HUMAN	Q6NXE6	Armadillo repeat-containing protein 6	3	99.58	0	0	0	0	0	0
ARPC4	ARPC4_HUMAN	P59998	Actin-related protein 2/3 complex subunit 4	0	0	0	0	2	99.58	0	0
ASNA1	ASNA_HUMAN	O43681	ATPase ASNA1	2	99.58	0	0	0	0	0	0
ASNS	ASNS_HUMAN	P08243	Asparagine synthetase [glutamine-hydrolyzing]	4	99.58	3	99.58	4	99.58	2	99.58
ATF7IP2	MCAF2_HUMAN	Q5U623	Activating transcription factor 7-interacting protein 2	0	0	0	0	0	0	2	89.98
ATG3	ATG3_HUMAN	Q9NT62	Ubiquitin-like-conjugating enzyme ATG3	8	99.58	3	99.58	6	99.58	0	0
ATIC	PUR9_HUMAN	P31939	Bifunctional purine biosynthesis protein PURH;Phosphoribosy	0	0	0	0	3	99.58	0	0
ATP6V1H	VATH_HUMAN	Q9UI12	V-type proton ATPase subunit H	2	99.58	0	0	0	0	0	0
ATXN10	ATX10_HUMAN	Q9UBB4	Ataxin-10	26	99.58	6	99.58	12	99.58	5	99.58
BAG6	BAG6_HUMAN	P46379	Large proline-rich protein BAG6	10	99.58	0	0	3	99.58	0	0

BAX	BAX_HUMAN	Q07812	Apoptosis regulator BAX	6	99.58	0	0	0	0	3	99.58
BCCIP	BCCIP_HUMAN	Q9P287	BRCA2 and CDKN1A-interacting protein	4	99.58	0	0	0	0	0	0
BCOR	BCOR_HUMAN	Q6W2J9	BCL-6 corepressor	0	0	8	99.58	0	0	0	0
BID	BID_HUMAN	P55957	BH3-interacting domain death agonist;BH3-interacting domai	4	99.58	0	0	0	0	0	0
BLK	BLK_HUMAN	P51451	Tyrosine-protein kinase Blk	2	99.58	2	99.58	2	99.58	2	99.58
BLOC1S2	BL1S2_HUMAN	Q6QNY1	Biogenesis of lysosome-related organelles complex 1 subunit	2	99.58	5	99.58	4	99.58	4	99.58
BLOC1S5	BL1S5_HUMAN	Q8TDH9	Biogenesis of lysosome-related organelles complex 1 subunit	0	0	0	0	4	99.58	4	99.58
BMI1	BMI1_HUMAN	P35226	Polycomb complex protein BMI-1	0	0	6	99.58	0	0	0	0
BOLA2B	BOLA2_HUMAN	Q9H3K6	BolA-like protein 2	4	99.58	6	99.58	5	99.58	5	99.58
BRAT1	BRAT1_HUMAN	Q6PJG6	BRCA1-associated ATM activator 1	2	99.58	0	0	0	0	0	0
BSDC1	BSDC1_HUMAN	Q9NW68	BSD domain-containing protein 1	0	0	3	99.58	0	0	0	0
BUB3	BUB3_HUMAN	O43684	Mitotic checkpoint protein BUB3	0	0	2	95.73	10	99.58	0	0
BYSL	BYST_HUMAN	Q13895	Bystin	3	99.58	0	0	0	0	0	0
BZW2	BZW2_HUMAN	Q9Y6E2	Basic leucine zipper and W2 domain-containing protein 2	2	99.58	0	0	0	0	0	0
CA2	CAH2_HUMAN	P00918	Carbonic anhydrase 2	2	99.58	8	99.58	9	99.58	4	99.58
CACYBP	CYBP_HUMAN	Q9HB71	Calcyclin-binding protein	20	99.58	14	99.58	12	99.58	12	99.58
CAD	PYR1_HUMAN	P27708	CAD protein;Glutamine-dependent carbamoyl-phosphate syn	22	99.58	12	99.58	14	99.58	2	99.58
CALM3	CALM_HUMAN	P62158	Calmodulin	5	99.58	6	99.58	10	99.58	4	99.58
CALML5	CALL5_HUMAN	Q9NZT1	Calmodulin-like protein 5	2	99.58	2	99.58	3	99.58	2	99.58
CAND1	CAND1_HUMAN	Q86VP6	Cullin-associated NEDD8-dissociated protein 1	69	99.58	35	99.58	43	99.58	35	99.58
CAP1	CAP1_HUMAN	Q01518	TNF receptor-associated factor 3	0	0	4	99.58	0	0	0	0
CAP2	CAP2_HUMAN	P40123	Adenylyl cyclase-associated protein 2	0	0	2	99.58	0	0	0	0
CAPN2	CAN2_HUMAN	P17655	Calpain-2 catalytic subunit	0	0	4	99.58	0	0	0	0
CAPNS1	CPNS1_HUMAN	P04632	Calpain small subunit 1	2	99.58	0	0	0	0	0	0
CARM1	CARM1_HUMAN	Q86X55	Histone-arginine methyltransferase CARM1	2	99.58	2	99.58	0	0	0	0
CBR1	CBR1_HUMAN	P16152	Carbonyl reductase [NADPH] 1	0	0	0	0	2	99.58	0	0
CBS	CBS_HUMAN	P35520	Cystathionine beta-synthase	2	99.58	0	0	0	0	4	99.58
CBX3	CBX3_HUMAN	Q13185	Chromobox protein homolog 3	4	99.58	0	0	0	0	0	0
CBX4	CBX4_HUMAN	O00257	E3 SUMO-protein ligase CBX4	0	0	4	99.58	0	0	0	0
CBX5	CBX5_HUMAN	P45973	Chromobox protein homolog 5	5	99.58	0	0	0	0	0	0
CBX8	CBX8_HUMAN	Q9HC52	Chromobox protein homolog 8	0	0	2	99.58	0	0	0	0
CCAR1	CCAR1_HUMAN	Q8IX12	Cell division cycle and apoptosis regulator protein 1	0	0	0	0	0	0	2	99.58
CCDC101	SGF29_HUMAN	Q96ES7	SAGA-associated factor 29 homolog	2	99.58	0	0	0	0	0	0
CCDC22	CCD22_HUMAN	O60826	Coiled-coil domain-containing protein 22	2	99.58	0	0	0	0	0	0
CCNB1	CCNB1_HUMAN	P14635	G2/mitotic-specific cyclin-B1	10	99.58	0	0	4	99.58	0	0
CCNT1	CCNT1_HUMAN	O60563	Cyclin-T1	0	0	0	0	4	99.58	0	0
CCT6B	TCPW_HUMAN	Q92526	T-complex protein 1 subunit zeta-2	4	99.58	4	99.58	5	99.58	4	99.58
CD2BP2	CD2B2_HUMAN	O95400	CD2 antigen cytoplasmic tail-binding protein 2	2	99.58	2	99.58	2	99.58	0	0
CDC37	CDC37_HUMAN	Q16543	Hsp90 co-chaperone Cdc37	4	99.58	2	99.58	3	99.58	3	99.58
CDC42	CDC42_HUMAN	P60953	Cell division control protein 42 homolog	0	0	4	99.58	2	99.58	0	0
CDC5L	CDC5L_HUMAN	Q99459	Cell division cycle 5-like protein	0	0	0	0	2	99.58	0	0
CDK12	CDK12_HUMAN	Q9NYV4	Cyclin-dependent kinase 12	2	99.58	2	99.58	2	99.58	2	99.58
CDK2	CDK2_HUMAN	P24941	Cyclin-dependent kinase 2	6	99.58	2	99.58	0	0	0	0
CDK2AP1	CDKA1_HUMAN	O14519	Cyclin-dependent kinase 2-associated protein 1	2	99.58	0	0	0	0	2	99.58
CDK3	CDK3_HUMAN	Q00526	Cyclin-dependent kinase 3	2	99.58	2	99.58	0	0	0	0
CDK9	CDK9_HUMAN	P50750	Cyclin-dependent kinase 9	0	0	0	0	0	0	2	99.58
CDV3	CDV3_HUMAN	Q9UKY7	Protein CDV3 homolog	0	0	0	0	2	99.58	0	0
CETN2	CETN2_HUMAN	P41208	Centrin-2	3	99.58	0	0	0	0	0	0
CHD1L	CHD1L_HUMAN	Q86WJ1	Chromodomain-helicase-DNA-binding protein 1-like	3	99.58	0	0	0	0	0	0
CHD3	CHD3_HUMAN	Q12873	Chromodomain-helicase-DNA-binding protein 3	8	99.58	2	99.58	2	99.58	3	99.58
CHD4	CHD4_HUMAN	Q14839	Chromodomain-helicase-DNA-binding protein 4	21	99.58	0	0	4	99.58	5	99.58
CLASP1	CLAP1_HUMAN	Q7Z460	CLIP-associating protein 1	7	99.58	2	99.58	2	99.58	0	0

CLASP2	CLAP2_HUMAN	O75122	CLIP-associating protein 2	2	99.58	0	0	0	0	2	99.58
CLTA	CLCA_HUMAN	P09496	Clathrin light chain A	3	99.58	2	99.58	2	99.58	0	0
CNOT1	CNOT1_HUMAN	A5YKK6	CCR4-NOT transcription complex subunit 1	49	99.58	2	94.07	3	99.58	6	99.58
CNOT10	CNOTA_HUMAN	Q9H9A5	CCR4-NOT transcription complex subunit 10	5	99.58	0	0	0	0	0	0
COBRA1	NELFB_HUMAN	Q8WX92	Negative elongation factor B	8	99.58	0	0	0	0	0	0
COG2	COG2_HUMAN	Q14746	Conserved oligomeric Golgi complex subunit 2	2	99.58	0	0	0	0	0	0
COMMD9	COMD9_HUMAN	Q9P000	COMM domain-containing protein 9	2	99.58	0	0	0	0	0	0
COPA	COPA_HUMAN	P53621	Coatomer subunit alpha;Xenin;Proxenin	70	99.58	13	99.58	19	99.58	13	99.58
COPE	COPE_HUMAN	O14579	Coatomer subunit epsilon	11	99.58	4	99.58	4	99.58	2	99.58
COPG1	COPG1_HUMAN	Q9Y678	Coatomer subunit gamma-1	28	99.58	2	99.58	6	99.58	2	99.58
COPG2	COPG2_HUMAN	Q9UBF2	Coatomer subunit gamma-2	14	99.58	3	99.58	3	99.58	0	0
COPS3	CSN3_HUMAN	Q9UNS2	COP9 signalosome complex subunit 3	2	99.58	0	0	0	0	0	0
COPS6	CSN6_HUMAN	Q7L5N1	COP9 signalosome complex subunit 6	0	0	0	0	0	0	2	99.58
COPZ1	COPZ1_HUMAN	P61923	Coatomer subunit zeta-1	8	99.58	0	0	3	98.6	4	99.58
COX17	COX17_HUMAN	Q14061	Cytochrome c oxidase copper chaperone	0	0	0	0	2	99.58	0	0
CPNE1	CPNE1_HUMAN	Q99829	Copine-1	2	99.58	0	0	0	0	0	0
CPSF3	CPSF3_HUMAN	Q9UKF6	Cleavage and polyadenylation specificity factor subunit 3	2	99.58	0	0	0	0	0	0
CPSF4	CPSF4_HUMAN	O95639	Cleavage and polyadenylation specificity factor subunit 4	2	99.58	0	0	0	0	0	0
CRKL	CRKL_HUMAN	P46109	Crk-like protein	2	88.72	0	0	0	0	3	87.2
CSNK2A1	CSK21_HUMAN	P68400	Casein kinase II subunit alpha	2	99.58	27	99.58	2	99.58	0	0
CSNK2A2	CSK22_HUMAN	P19784	Casein kinase II subunit alpha'	0	0	13	99.58	0	0	0	0
CSNK2B	CSK2B_HUMAN	P67870	Casein kinase II subunit beta	0	0	18	99.58	0	0	2	99.58
CSTA	CYTA_HUMAN	P01040	Cystatin-A	6	99.58	2	99.58	7	99.58	0	0
CSTF1	CSTF1_HUMAN	Q05048	Cleavage stimulation factor subunit 1	2	99.58	4	99.58	0	0	0	0
CTBP1	CTBP1_HUMAN	Q13363	C-terminal-binding protein 1	9	99.58	4	99.58	5	99.58	3	99.58
CTNNBL1	CTBL1_HUMAN	Q8WYA6	Beta-catenin-like protein 1	3	99.58	3	99.12	0	0	3	93.53
CTPS1	PYRG1_HUMAN	P17812	CTP synthase 1	16	99.58	10	99.58	13	99.58	2	99.58
CUL1	CUL1_HUMAN	Q13616	Cullin-1	3	99.58	0	0	2	99.58	0	0
CUL3	CUL3_HUMAN	Q13618	Cullin-3	4	99.58	0	0	0	0	0	0
CUL4A	CUL4A_HUMAN	Q13619	Cullin-4A	9	99.58	2	99.58	3	99.58	0	0
CUL4B	CUL4B_HUMAN	Q13620	Cullin-4B	10	99.58	0	0	0	0	0	0
CYCS	CYC_HUMAN	P99999	Cytochrome c	0	0	3	99.58	3	99.58	0	0
DBI	ACBP_HUMAN	P07108	Acyl-CoA-binding protein	0	0	0	0	3	99.58	0	0
DCAF7	DCAF7_HUMAN	P61962	DDB1- and CUL4-associated factor 7	2	99.58	9	99.58	0	0	2	99.58
DCP2	DCP2_HUMAN	Q8IU60	m7GpppN-mRNA hydrolase	2	99.58	0	0	0	0	0	0
DCTN1	DCTN1_HUMAN	Q14203	Dynactin subunit 1	26	99.58	0	0	2	99.58	0	0
DCTN2	DCTN2_HUMAN	Q13561	Dynactin subunit 2	6	99.58	0	0	0	0	3	99.58
DCTN5	DCTN5_HUMAN	Q9BTE1	Dynactin subunit 5	0	0	0	0	2	99.58	0	0
DCXR	DCXR_HUMAN	Q7Z4W1	L-xylulose reductase	0	0	0	0	2	99.58	0	0
DDB1	DDB1_HUMAN	Q16531	DNA damage-binding protein 1	3	99.58	0	0	3	99.58	0	0
DDX1	DDX1_HUMAN	Q92499	ATP-dependent RNA helicase DDX1	0	0	0	0	2	99.58	0	0
DDX19A	DD19A_HUMAN	Q9NUU7	ATP-dependent RNA helicase DDX19A	23	99.58	12	99.58	15	99.58	5	99.58
DDX19B	DD19B_HUMAN	Q9UMR2	ATP-dependent RNA helicase DDX19B	2	99.58	0	0	0	0	0	0
DDX3Y	DDX3Y_HUMAN	O15523	ATP-dependent RNA helicase DDX3Y	2	99.58	0	0	0	0	0	0
DDX42	DDX42_HUMAN	Q86XP3	ATP-dependent RNA helicase DDX42	0	0	0	0	0	0	2	99.58
DIAPH1	DIAP1_HUMAN	O60610	Protein diaphanous homolog 1	10	99.58	0	0	0	0	0	0
DIAPH3	DIAP3_HUMAN	Q9NSV4	Protein diaphanous homolog 3	4	97.63	0	0	0	0	0	0
DIS3	RRP44_HUMAN	Q9Y2L1	Exosome complex exonuclease RRP44	17	99.58	6	99.58	5	99.58	6	99.58
DNAJB1	DNJB1_HUMAN	P25685	DnaJ homolog subfamily B member 1	0	0	0	0	2	99.58	0	0
DNM1	DYN1_HUMAN	Q05193	Dynamin-1	9	99.58	2	99.58	7	99.58	4	99.58
DNM1L	DNM1L_HUMAN	O00429	Dynamin-1-like protein	33	99.58	3	99.58	8	99.58	4	99.58
DNM2	DYN2_HUMAN	P50570	Dynamin-2	36	99.58	4	99.58	7	99.58	9	99.58

DNM3	DYN3_HUMAN	Q9UQ16	Dynamin-3	2	99.58	0	0	0	0	0	0
DRG1	DRG1_HUMAN	Q9Y295	Protein NDRG1	9	99.58	4	99.58	4	99.58	5	99.58
DRG2	DRG2_HUMAN	P55039	Developmentally-regulated GTP-binding protein 2	3	99.58	0	0	0	0	0	0
DSC1	DSC1_HUMAN	Q08554	Calcipressin-1	4	99.58	2	99.58	3	99.58	2	99.58
DTNBP1	DTBP1_HUMAN	Q96EV8	Dysbindin	0	0	3	99.58	0	0	0	0
DUT	DUT_HUMAN	P33316	Deoxyuridine 5'-triphosphate nucleotidohydrolase, mitochond	0	0	0	0	2	99.58	0	0
DYNC1I2	DC1I2_HUMAN	Q13409	Cytoplasmic dynein 1 intermediate chain 2	2	99.58	0	0	0	0	0	0
DYNC1LI1	DC1L1_HUMAN	Q9Y6G9	Cytoplasmic dynein 1 light intermediate chain 1	4	99.58	0	0	2	99.58	0	0
DYNLRB1	DLRB1_HUMAN	Q9NP97	Dynein light chain roadblock-type 1	6	99.58	4	99.58	3	99.58	4	99.58
ECHDC1	ECHD1_HUMAN	Q9NTX5	Ethylmalonyl-CoA decarboxylase	3	99.58	0	0	0	0	0	0
ECM29	ECM29_HUMAN	Q5VYK3	Proteasome-associated protein ECM29 homolog	4	99.58	0	0	0	0	0	0
EDF1	EDF1_HUMAN	O60869	Endothelial differentiation-related factor 1	0	0	2	99.58	4	99.58	0	0
EEF1E1	MCA3_HUMAN	O43324	Eukaryotic translation elongation factor 1 epsilon-1	2	99.58	2	99.58	4	99.58	2	99.58
EHD1	EHD1_HUMAN	Q9H4M9	EH domain-containing protein 1	6	99.58	0	0	0	0	0	0
EIF2AK2	E2AK2_HUMAN	P19525	Interferon-induced, double-stranded RNA-activated protein ki	3	99.58	0	0	0	0	0	0
EIF2B1	EI2BA_HUMAN	Q14232	Translation initiation factor eIF-2B subunit alpha	5	99.58	0	0	0	0	0	0
EIF3A	EIF3A_HUMAN	Q14152	Eukaryotic translation initiation factor 6	14	99.58	9	99.58	9	99.58	12	99.58
EIF3B	EIF3B_HUMAN	P55884	Eukaryotic translation initiation factor 3 subunit B	15	99.58	15	99.58	12	99.58	5	99.58
EIF3CL	EIF3C_HUMAN	Q99613	Eukaryotic translation initiation factor 3 subunit C	8	99.58	5	99.58	4	99.58	7	99.58
EIF3D	EIF3D_HUMAN	O15371	Eukaryotic translation initiation factor 3 subunit D	2	99.58	4	99.58	0	0	0	0
EIF3E	EIF3E_HUMAN	P60228	Eukaryotic translation initiation factor 3 subunit E	11	99.58	5	99.58	8	99.58	3	99.58
EIF3F	EIF3F_HUMAN	O00303	Eukaryotic translation initiation factor 3 subunit F	12	99.58	9	99.58	8	99.58	5	99.58
EIF3K	EIF3K_HUMAN	Q9UBQ5	Eukaryotic translation initiation factor 3 subunit K	3	99.58	0	0	2	99.58	2	99.58
EIF3L	EIF3L_HUMAN	Q9Y262	Eukaryotic translation initiation factor 3 subunit L	15	99.58	7	99.58	11	99.58	6	99.58
EIF3M	EIF3M_HUMAN	Q7L2H7	Eukaryotic translation initiation factor 3 subunit M	8	99.58	2	99.58	5	99.58	0	0
EIF4A2	IF4A2_HUMAN	Q14240	Eukaryotic initiation factor 4A-II	5	99.58	0	0	2	99.58	2	99.58
EIF4E1B	I4E1B_HUMAN	A6NMX2	Eukaryotic translation initiation factor 4E type 1B	2	99.58	0	0	0	0	0	0
EIF4G2	IF4G2_HUMAN	P78344	Eukaryotic translation initiation factor 4 gamma 2	2	99.58	0	0	0	0	0	0
EIF4H	IF4H_HUMAN	Q15056	Eukaryotic translation initiation factor 4H	0	0	3	99.58	0	0	0	0
EIF5	IF5_HUMAN	P55010	Eukaryotic translation initiation factor 5	0	0	2	99.58	0	0	0	0
EIF5B	IF2P_HUMAN	O60841	Eukaryotic translation initiation factor 5B	0	0	2	99.58	2	99.58	0	0
EMSY	EMSY_HUMAN	Q7Z589	Protein EMSY	1	99.24	0	0	0	0	0	0
ERP29	ERP29_HUMAN	P30040	Endoplasmic reticulum resident protein 29	0	0	2	99.58	0	0	0	0
ETF1	ERF1_HUMAN	P62495	Eukaryotic peptide chain release factor subunit 1	4	99.58	2	99.58	3	99.58	0	0
EXOC1	EXOC1_HUMAN	Q9NV70	Exocyst complex component 1	3	99.58	0	0	0	0	0	0
EXOC2	EXOC2_HUMAN	Q96KP1	Exocyst complex component 2	7	99.58	0	0	0	0	0	0
EXOC4	EXOC4_HUMAN	Q96A65	Exocyst complex component 4	8	99.58	0	0	0	0	0	0
EXOC6	EXOC6_HUMAN	Q8TAG9	Exocyst complex component 6	2	99.58	0	0	0	0	0	0
EXOC7	EXOC7_HUMAN	Q9UPT5	Exocyst complex component 7	2	99.58	0	0	0	0	0	0
EXOSC4	EXOS4_HUMAN	Q9NPD3	Exosome complex component RRP41	0	0	2	99.58	2	99.58	0	0
EZR	EZRI_HUMAN	P15311	Ezrin	0	0	5	99.58	8	99.58	0	0
FAF1	FAF1_HUMAN	Q9UNN5	FAS-associated factor 1	5	99.58	2	99.58	0	0	0	0
FAM114A2	F1142_HUMAN	Q9NRY5	Protein FAM114A2	6	99.58	7	99.58	0	0	0	0
FAM50A	FA50A_HUMAN	Q14320	Protein FAM50A	6	99.58	4	99.58	0	0	4	99.58
FAM50B	FA50B_HUMAN	Q9Y247	Protein FAM50B	0	0	0	0	0	0	2	99.58
FAM98A	FA98A_HUMAN	Q8NCA5	Protein FAM98A	0	0	0	0	4	99.58	0	0
FANCD2	FACD2_HUMAN	Q9BXW9	Fanconi anemia group D2 protein	5	99.58	0	0	0	0	0	0
FANCI	FANCI_HUMAN	Q9NVI1	Fanconi anemia group I protein	40	99.58	2	99.58	10	99.58	5	99.58
FARSB	SYFB_HUMAN	Q9NSD9	Phenylalanine--tRNA ligase beta subunit	2	99.58	0	0	0	0	0	0
FEN1	FEN1_HUMAN	P39748	Flap endonuclease 1	18	99.58	12	99.58	11	99.58	13	99.58
FERMT1	FERM1_HUMAN	Q9BQL6	Fermitin family homolog 1	2	99.58	0	0	0	0	0	0
FERMT2	FERM2_HUMAN	Q96AC1	Fermitin family homolog 2	13	99.58	2	99.58	2	99.58	0	0

FHL1	FHL1_HUMAN	Q13642	Four and a half LIM domains protein 1	2	99.58	2	99.58	3	99.58	3	99.58
FKBP15	FKB15_HUMAN	Q5T1M5	FK506-binding protein 15	2	99.58	0	0	0	0	0	0
FKBP1A	FKB1A_HUMAN	P62942	Peptidyl-prolyl cis-trans isomerase FKBP1A	2	99.58	3	99.58	5	99.58	4	99.58
FKBP3	FKBP3_HUMAN	Q00688	Peptidyl-prolyl cis-trans isomerase FKBP3	0	0	0	0	2	99.58	0	0
FN3KRP	KT3K_HUMAN	Q9HA64	Ketosamine-3-kinase	4	99.58	3	99.58	2	99.58	7	99.58
FSCN1	FSCN1_HUMAN	Q16658	Fascin	0	0	0	0	2	81.33	0	0
FUBP1	FUBP1_HUMAN	Q96AE4	Far upstream element-binding protein 1	0	0	3	99.58	5	99.58	0	0
GADD45GIP	G45IP_HUMAN	Q8TAE8	Growth arrest and DNA damage-inducible proteins-interacting	0	0	0	0	0	0	2	99.58
GAPDHS	G3PT_HUMAN	O14556	Glyceraldehyde-3-phosphate dehydrogenase, testis-specific	0	0	2	99.58	2	99.58	0	0
GAPVD1	GAPD1_HUMAN	Q14C86	GTPase-activating protein and VPS9 domain-containing protei	12	99.58	0	0	2	99.58	2	99.58
GARS	SYG_HUMAN	P41250	Glycine--tRNA ligase	0	0	0	0	2	99.58	0	0
GART	PUR2_HUMAN	P22102	Trifunctional purine biosynthetic protein adenosine-3;Phosph	0	0	3	99.58	0	0	2	99.58
GATAD2B	P66B_HUMAN	Q8WXI9	Transcriptional repressor p66-beta	0	0	0	0	1	99.58	0	0
GBF1	GBF1_HUMAN	Q92538	Golgi-specific brefeldin A-resistance guanine nucleotide excha	5	99.58	0	0	3	99.58	0	0
GDI1	GDIA_HUMAN	P31150	Rab GDP dissociation inhibitor alpha	0	0	0	0	2	99.58	0	0
GDI2	GDIB_HUMAN	P50395	Rab GDP dissociation inhibitor beta	0	0	3	99.58	3	99.58	0	0
GEMIN2	GEMI2_HUMAN	O14893	Gem-associated protein 2	0	0	3	99.58	0	0	4	99.58
GEMIN4	GEMI4_HUMAN	P57678	Gem-associated protein 4	11	99.58	7	99.58	2	99.58	13	99.58
GEMIN5	GEMI5_HUMAN	Q8TEQ6	Gem-associated protein 5	2	99.58	2	99.58	0	0	3	99.58
GEMIN6	GEMI6_HUMAN	Q8WXD5	Gem-associated protein 6	0	0	4	99.58	0	0	2	99.58
GEMIN7	GEMI7_HUMAN	Q9H840	Gem-associated protein 7	0	0	0	0	2	99.58	0	0
GEMIN8	GEMI8_HUMAN	Q9NWZ8	Gem-associated protein 8	2	99.58	2	99.58	0	0	2	99.36
GET4	GET4_HUMAN	Q7L5D6	Golgi to ER traffic protein 4 homolog	2	99.58	0	0	0	0	0	0
GGCT	GGCT_HUMAN	O75223	Gamma-glutamylcyclotransferase	0	0	0	0	2	99.58	0	0
GLMN	GLMN_HUMAN	Q92990	Glomulin	2	99.58	0	0	0	0	0	0
GLOD4	GLOD4_HUMAN	Q9HC38	Glyoxalase domain-containing protein 4	0	0	0	0	4	99.58	0	0
GLRX3	GLRX3_HUMAN	O76003	Glutaredoxin-3	0	0	0	0	4	99.58	0	0
GLUL	GLNA_HUMAN	P15104	Glutamine synthetase	2	99.58	2	99.58	2	99.58	0	0
GMPS	GUAA_HUMAN	P49915	GMP synthase [glutamine-hydrolyzing]	2	99.58	2	99.58	3	99.58	0	0
GPS1	CSN1_HUMAN	Q13098	COP9 signalosome complex subunit 1	2	99.58	0	0	2	99.58	0	0
GTF2E2	T2EB_HUMAN	P29084	Transcription initiation factor IIE subunit beta	0	0	3	99.58	0	0	0	0
GTF2I	GTF2I_HUMAN	P78347	General transcription factor II-I	20	99.58	3	99.58	5	99.58	7	99.58
GTPBP1	GTPB1_HUMAN	O00178	GTP-binding protein 1	11	99.58	0	0	0	0	2	99.58
HAGH	GLO2_HUMAN	Q16775	Hydroxyacylglutathione hydrolase, mitochondrial	0	0	2	99.58	0	0	4	99.58
HAUS3	HAUS3_HUMAN	Q68CZ6	HAUS augmin-like complex subunit 3	3	99.58	0	0	0	0	0	0
HAUS5	HAUS5_HUMAN	O94927	HAUS augmin-like complex subunit 5	2	99.58	0	0	0	0	0	0
HCFC1	HCFC1_HUMAN	P51610	Host cell factor 1;HCF N-terminal chain 1;HCF N-terminal chai	3	99.58	6	99.58	0	0	2	99.58
HDAC1	HDAC1_HUMAN	Q13547	Histone deacetylase 1	14	99.58	0	0	26	99.58	0	0
HDAC2	HDAC2_HUMAN	Q92769	Histone deacetylase 2	1	99.58	2	99.58	6	99.58	0	0
HDAC6	HDAC6_HUMAN	Q9UBN7	Histone deacetylase 6	2	99.58	0	0	0	0	0	0
HDGF	HDGF_HUMAN	P51858	Hepatoma-derived growth factor	0	0	5	99.58	5	99.58	0	0
HEATR1	HEAT1_HUMAN	Q9H583	HEAT repeat-containing protein 1	6	99.58	0	0	0	0	0	0
HEATR2	HEAT2_HUMAN	Q86Y56	HEAT repeat-containing protein 2	9	99.58	0	0	2	99.58	0	0
HEATR3	HEAT3_HUMAN	Q7Z4Q2	HEAT repeat-containing protein 3	3	99.58	0	0	0	0	2	99.58
HEATR6	HEAT6_HUMAN	Q6AI08	HEAT repeat-containing protein 6	3	99.58	0	0	0	0	0	0
HELLS	HELLS_HUMAN	Q9NRZ9	Lymphoid-specific helicase	9	99.58	0	0	0	0	0	0
HIP1R	HIP1R_HUMAN	O75146	Huntingtin-interacting protein 1-related protein	2	99.58	0	0	0	0	0	0
HIST1H1A	H11_HUMAN	Q02539	Histone H1.1	4	99.58	0	0	0	0	0	0
HIST1H1C	H12_HUMAN	P16403	Histone H1.2	14	99.58	0	0	0	0	0	0
HIST1H1E	H14_HUMAN	P10412	Histone H1.4	2	99.58	2	99.58	2	99.58	2	99.58
HIST1H2AA	H2A1A_HUMAN	Q96QV6	Histone H2A type 1-A	5	99.58	0	0	2	99.58	3	99.58
HIST1H2AD	H2A1D_HUMAN	P20671	Histone H2A type 1-D	14	99.58	0	0	1	99.58	1	99.58

HIST1H2AE	H2A1B_HUMAN	P04908	Histone H2A type 1-B/E	10	99.58	0	0	1	99.58	1	99.58
HIST1H2BA	H2B1A_HUMAN	Q96A08	Histone H2B type 1-A	8	99.58	0	0	0	0	0	0
HIST1H2BB	H2B1B_HUMAN	P33778	Histone H2B type 1-B	7	99.58	0	0	0	0	0	0
HIST1H2BI	H2B1C_HUMAN	P62807	Histone H2B type 1-C/E/F/G/I	6	99.58	0	0	0	0	0	0
HIST3H3	H31T_HUMAN	Q16695	Histone H3.1t	7	90.04	0	0	1	61.33	0	0
HIST4H4	H4_HUMAN	P62805	Histone H4	24	99.58	0	0	0	0	0	0
HN1	HN1_HUMAN	Q9UK76	Hematological and neurological expressed 1 protein	0	0	2	99.58	0	0	0	0
HN1L	HN1L_HUMAN	Q9H910	Hematological and neurological expressed 1-like protein	0	0	0	0	3	99.58	3	99.58
HSBP1	HSBP1_HUMAN	O75506	Heat shock factor-binding protein 1	0	0	2	99.58	0	0	0	0
HSP90AA5P	HS905_HUMAN	Q58FG0	Putative heat shock protein HSP 90-alpha A5	11	99.58	9	99.58	9	99.58	5	99.58
HSPA4	HSP74_HUMAN	P34932	Heat shock 70 kDa protein 4	5	99.58	8	99.58	9	99.58	4	99.58
HSPB1	HSPB1_HUMAN	P04792	Heat shock protein beta-1	5	99.58	4	99.58	3	99.58	3	99.58
HSPE1	CH10_HUMAN	P61604	10 kDa heat shock protein, mitochondrial	0	0	4	99.58	5	99.58	0	0
HSPH1	HS105_HUMAN	Q92598	Heat shock protein 105 kDa	7	99.58	4	99.58	8	99.58	0	0
HTT	HD_HUMAN	P42858	Sodium-dependent serotonin transporter	2	99.58	0	0	0	0	0	0
HUWE1	HUWE1_HUMAN	Q7Z6Z7	E3 ubiquitin-protein ligase HUWE1	14	99.58	2	99.58	0	0	0	0
IGF2BP3	IF2B3_HUMAN	O00425	Insulin-like growth factor 2 mRNA-binding protein 3	2	99.58	2	99.58	0	0	2	99.58
IK	RED_HUMAN	Q13123	Protein Red	0	0	6	99.58	0	0	0	0
IKBKAP	ELP1_HUMAN	O95163	Elongator complex protein 1	4	99.58	0	0	0	0	0	0
ILK	ILK_HUMAN	Q13418	Integrin-linked protein kinase	6	99.58	0	0	0	0	0	0
IPO11	IPO11_HUMAN	Q9UI26	Importin-11	2	99.58	0	0	0	0	0	0
IPO4	IPO4_HUMAN	Q8TEX9	Importin-4	17	99.58	0	0	3	95.74	0	0
IPO5	IPO5_HUMAN	O00410	Importin-5	62	99.58	17	99.58	34	99.58	19	99.58
IPO7	IPO7_HUMAN	O95373	Importin-7	8	99.58	4	99.58	0	0	0	0
IPO8	IPO8_HUMAN	O15397	Importin-8	6	99.58	4	99.58	7	99.58	3	99.58
IPO9	IPO9_HUMAN	Q96P70	Importin-9	29	99.58	10	99.58	10	99.58	49	99.58
IRAK1	IRAK1_HUMAN	P51617	Interleukin-1 receptor-associated kinase 1	2	99.58	0	0	0	0	0	0
IRS4	IRS4_HUMAN	O14654	Insulin receptor substrate 4	2	99.58	2	99.58	2	99.58	2	99.58
IST1	IST1_HUMAN	P53990	IST1 homolog	4	99.58	0	0	0	0	0	0
IVNS1ABP	NS1BP_HUMAN	Q9Y6Y0	Influenza virus NS1A-binding protein	7	99.58	6	99.58	4	99.58	0	0
KARS	SYK_HUMAN	Q15046	Lysine--tRNA ligase	0	0	2	99.58	2	99.58	0	0
KATNAL2	KATL2_HUMAN	Q8IYT4	Katanin p60 ATPase-containing subunit A-like 2	0	0	2	99.58	0	0	0	0
KCTD12	KCD12_HUMAN	Q96CX2	BTB/POZ domain-containing protein KCTD12	3	99.58	0	0	2	99.58	0	0
KDM2B	KDM2B_HUMAN	Q8NHM5	Lysine-specific demethylase 2B	0	0	2	99.58	0	0	0	0
KIAA0196	STRUM_HUMAN	Q12768	WASH complex subunit strumpellin	3	99.58	0	0	0	0	0	0
KIAA1033	WASH7_HUMAN	Q2M389	WASH complex subunit 7	4	99.58	0	0	0	0	0	0
KIAA1279	KBP_HUMAN	Q96EK5	KIF1-binding protein	2	99.58	0	0	0	0	0	0
KIAA1468	K1468_HUMAN	Q9P260	LisH domain and HEAT repeat-containing protein KIAA1468	2	99.58	0	0	0	0	0	0
KIAA1524	CIP2A_HUMAN	Q8TCG1	Protein CIP2A	12	99.58	0	0	0	0	0	0
KIAA1967	K1967_HUMAN	Q8N163	DBIRD complex subunit KIAA1967	0	0	5	99.58	2	97.94	0	0
KIF11	KIF11_HUMAN	P52732	Kinesin-like protein KIF11	34	99.58	18	99.58	14	99.58	27	99.58
KPNA1	IMA1_HUMAN	P52294	Importin subunit alpha-1	13	99.58	2	99.58	4	99.58	2	99.58
KPNA3	IMA3_HUMAN	O00505	Importin subunit alpha-3	2	99.58	0	0	0	0	0	0
KPNA6	IMA7_HUMAN	O60684	Importin subunit alpha-7	5	99.58	0	0	0	0	0	0
KPNA7	IMA8_HUMAN	A9QM74	Importin subunit alpha-8	2	99.58	0	0	0	0	0	0
KPRP	KPRP_HUMAN	Q5T749	Keratinocyte proline-rich protein	2	99.58	0	0	0	0	0	0
LANCL1	LANC1_HUMAN	O43813	LanC-like protein 1	0	0	0	0	0	0	4	99.58
LIG1	DNLI1_HUMAN	P18858	Leucine-rich repeats and immunoglobulin-like domains protei	3	99.58	0	0	0	0	0	0
LIN7A	LIN7A_HUMAN	O14910	Protein lin-7 homolog A	2	99.58	0	0	0	0	0	0
LRBA	LRBA_HUMAN	P50851	Lipopolysaccharide-responsive and beige-like anchor protein	5	99.58	0	0	0	0	0	0
LRRC40	LRC40_HUMAN	Q9H9A6	Leucine-rich repeat-containing protein 40	15	99.58	6	99.58	7	99.58	7	99.58
LTA4H	LKHA4_HUMAN	P09960	Leukotriene A-4 hydrolase	0	0	0	0	3	99.58	0	0

LYPLA2	LYPA2_HUMAN	O95372	Acyl-protein thioesterase 2	5	99.58	0	0	0	0	0	0
MAD2L1	MD2L1_HUMAN	Q13257	Mitotic spindle assembly checkpoint protein MAD2A	2	99.58	0	0	0	0	0	0
MAGED2	MAGD2_HUMAN	Q9UNF1	Melanoma-associated antigen D2	17	99.58	4	99.58	5	99.58	7	99.58
MAP2K1	MP2K1_HUMAN	Q02750	Dual specificity mitogen-activated protein kinase kinase 1	14	99.58	3	99.58	6	99.58	9	99.58
MAP2K2	MP2K2_HUMAN	P36507	Dual specificity mitogen-activated protein kinase kinase 2	14	99.58	2	99.58	3	99.58	3	99.58
MAP2K4	MP2K4_HUMAN	P45985	Dual specificity mitogen-activated protein kinase kinase 4	2	99.58	0	0	0	0	0	0
MAP4	MAP4_HUMAN	P27816	Microtubule-associated protein 4	2	99.58	2	99.58	0	0	0	0
MAPRE1	MARE1_HUMAN	Q15691	Microtubule-associated protein RP/EB family member 1	4	99.58	6	99.58	5	99.58	0	0
MARS	SYMC_HUMAN	P56192	Methionine--tRNA ligase, cytoplasmic	11	99.58	5	99.58	5	99.58	4	99.58
MBD3	MBD3_HUMAN	O95983	Methyl-CpG-binding domain protein 3	0	0	0	0	5	99.58	0	0
MDN1	MDN1_HUMAN	Q9NU22	Midasin	5	99.58	0	0	0	0	0	0
MED1	MED1_HUMAN	Q15648	Mediator of RNA polymerase II transcription subunit 1	3	99.58	0	0	0	0	0	0
MEF2C	MEF2C_HUMAN	Q06413	Myocyte-specific enhancer factor 2C	0	0	0	0	2	86.93	2	86.26
METTL16	MET16_HUMAN	Q86W50	Methyltransferase-like protein 16	2	99.58	0	0	0	0	0	0
MITD1	MITD1_HUMAN	Q8WV92	MIT domain-containing protein 1	0	0	0	0	2	92.57	0	0
MKI67	KI67_HUMAN	P46013	Antigen KI-67	2	82.42	0	0	0	0	0	0
MMS19	MMS19_HUMAN	Q96T76	MMS19 nucleotide excision repair protein homolog	3	99.58	0	0	0	0	0	0
MMS22L	MMS22_HUMAN	Q6ZRQ5	Protein MMS22-like	0	0	0	0	0	0	2	86.93
MOB1A	MOB1A_HUMAN	Q9H8S9	MOB kinase activator 1A	4	99.58	0	0	2	99.58	4	99.58
MOB1B	MOB1B_HUMAN	Q7L9L4	MOB kinase activator 1B	10	99.58	4	99.58	7	99.58	9	99.58
MON2	MON2_HUMAN	Q7Z3U7	Protein MON2 homolog	4	99.58	0	0	0	0	0	0
MSH2	MSH2_HUMAN	P43246	DNA mismatch repair protein Msh2	2	99.58	0	0	0	0	0	0
MSH6	MSH6_HUMAN	P52701	DNA mismatch repair protein Msh6	4	99.58	0	0	0	0	0	0
MST4	MST4_HUMAN	Q9P289	Serine/threonine-protein kinase MST4	3	99.58	2	88.85	3	97.66	3	99.58
MTA1	MTA1_HUMAN	Q13330	Metastasis-associated protein MTA1	0	0	0	0	8	99.58	0	0
MTA2	MTA2_HUMAN	O94776	Metastasis-associated protein MTA2	0	0	0	0	19	99.58	0	0
MTAP	MTAP_HUMAN	Q13126	S-methyl-5'-thioadenosine phosphorylase	0	0	0	0	3	99.58	0	0
MTOR	MTOR_HUMAN	P42345	Serine/threonine-protein kinase mTOR	4	99.58	0	0	0	0	0	0
MTPN	MTPN_HUMAN	P58546	Myotrophin	2	99.58	4	99.58	4	99.58	4	99.58
MYCBP	MYCBP_HUMAN	Q99417	C-Myc-binding protein	2	99.58	6	99.58	7	99.58	5	99.58
NACA	NACA_HUMAN	Q13765	Nascent polypeptide-associated complex subunit alpha	0	0	3	99.58	5	99.58	0	0
NAMPT	NAMPT_HUMAN	P43490	Nicotinamide phosphoribosyltransferase	24	99.58	6	99.58	15	99.58	8	99.58
NAP1L4	NP1L4_HUMAN	Q99733	Nucleosome assembly protein 1-like 4	2	99.58	2	99.58	2	99.58	2	99.58
NCAPD2	CND1_HUMAN	Q15021	Condensin complex subunit 1	18	99.58	0	0	3	99.58	0	0
NCAPD3	CNDD3_HUMAN	P42695	Condensin-2 complex subunit D3	2	99.58	0	0	0	0	0	0
NCAPG	CND3_HUMAN	Q9BPX3	Condensin complex subunit 3	10	99.58	0	0	7	99.58	0	0
NCAPG2	CNDG2_HUMAN	Q86XI2	Condensin-2 complex subunit G2	2	99.58	0	0	0	0	0	0
NCAPH	CND2_HUMAN	Q15003	Condensin complex subunit 2	6	99.58	2	99.58	2	99.58	0	0
NCBP1	NCBP1_HUMAN	Q09161	Nuclear cap-binding protein subunit 1	5	99.58	0	0	0	0	0	0
NDRG1	NDRG1_HUMAN	Q92597	Protein NDRG1	3	99.58	3	99.58	0	0	0	0
NDRG3	NDRG3_HUMAN	Q9UGV2	Protein NDRG3	2	99.58	0	0	0	0	0	0
NEK7	NEK7_HUMAN	Q8TDX7	Serine/threonine-protein kinase Nek7	2	99.58	0	0	0	0	0	0
NEK9	NEK9_HUMAN	Q8TD19	Serine/threonine-protein kinase Nek9	7	99.58	0	0	0	0	0	0
NOC4L	NOC4L_HUMAN	Q9BVI4	Nucleolar complex protein 4 homolog	0	0	0	0	0	0	2	99.58
NPM3	NPM3_HUMAN	O75607	Nucleoplasmin-3	2	99.58	0	0	2	99.58	0	0
NSRP1	NSRP1_HUMAN	Q9H0G5	Nuclear speckle splicing regulatory protein 1	2	99.58	2	99.58	0	0	3	99.58
NTPCR	NTPCR_HUMAN	Q9BSD7	Cancer-related nucleoside-triphosphatase	2	99.58	0	0	2	99.58	0	0
NUBP2	NUBP2_HUMAN	Q9Y5Y2	Cytosolic Fe-S cluster assembly factor NUBP2	2	99.58	0	0	2	99.58	0	0
NUDC	NUDC_HUMAN	Q9Y266	Nuclear migration protein nudC	20	99.58	15	99.58	13	99.58	10	99.58
NUP107	NU107_HUMAN	P57740	Nuclear pore complex protein Nup107	2	82.81	0	0	0	0	0	0
NUP133	NU133_HUMAN	Q8WUM0	Nuclear pore complex protein Nup133	13	99.58	0	0	0	0	0	0
NUP160	NU160_HUMAN	Q12769	Nuclear pore complex protein Nup160	2	99.58	0	0	0	0	0	0

NUP205	NU205_HUMAN	Q92621	Nuclear pore complex protein Nup205	17	99.58	0	0	3	99.58	2	99.58
NUPL1	NUPL1_HUMAN	Q9BVL2	Nucleoporin p58/p45	3	99.58	0	0	0	0	0	0
NUSAP1	NUSAP_HUMAN	Q9BXS6	Nucleolar and spindle-associated protein 1	0	0	3	99.58	0	0	0	0
OGT	OGT1_HUMAN	O15294	UDP-N-acetylglucosamine--peptide N-acetylglucosaminyltrans	0	0	4	99.58	2	99.58	5	99.58
ORC3	ORC3_HUMAN	Q9UBD5	Origin recognition complex subunit 3	6	99.58	0	0	0	0	0	0
ORC4	ORC4_HUMAN	O43929	Origin recognition complex subunit 4	3	99.58	0	0	0	0	0	0
ORC5	ORC5_HUMAN	O43913	Origin recognition complex subunit 5	4	99.58	0	0	0	0	0	0
OSBP	OSBP1_HUMAN	P22059	Oxysterol-binding protein 1	2	99.58	8	99.58	13	99.58	4	96.57
OSBP2	OSBP2_HUMAN	Q969R2	Oxysterol-binding protein 2	2	99.58	2	99.58	2	99.58	2	99.58
OTOF	OTOF_HUMAN	Q9HC10	Otoferlin	0	0	2	99.58	0	0	0	0
OTUB1	OTUB1_HUMAN	Q96FW1	Ubiquitin thioesterase OTUB1	12	99.58	12	99.58	13	99.58	10	99.58
OXSR1	OXSR1_HUMAN	O95747	Serine/threonine-protein kinase OSR1	15	99.58	4	99.58	5	99.58	8	99.58
PAICS	PUR6_HUMAN	P22234	Multifunctional protein ADE2;Phosphoribosylaminoimidazole-	2	99.58	2	99.58	9	99.58	0	0
PAK4	PAK4_HUMAN	O96013	Serine/threonine-protein kinase PAK 4	2	99.58	0	0	0	0	0	0
PCBD1	PHS_HUMAN	P61457	Pterin-4-alpha-carbinolamine dehydratase	2	99.58	0	0	0	0	0	0
PCGF1	PCGF1_HUMAN	Q9BSM1	Polycomb group RING finger protein 1	0	0	5	99.58	0	0	0	0
PCGF3	PCGF3_HUMAN	Q3KNV8	Polycomb group RING finger protein 3	0	0	5	99.58	0	0	0	0
PCGF5	PCGF5_HUMAN	Q86SE9	Polycomb group RING finger protein 5	0	0	4	99.58	0	0	0	0
PCGF6	PCGF6_HUMAN	Q9BYE7	Polycomb group RING finger protein 6	0	0	2	99.58	0	0	0	0
PCNP	PCNP_HUMAN	Q8WW12	PEST proteolytic signal-containing nuclear protein	0	0	3	99.58	0	0	0	0
PDAP1	HAP28_HUMAN	Q13442	28 kDa heat- and acid-stable phosphoprotein	3	99.58	2	99.58	3	99.58	0	0
PDCD10	PDC10_HUMAN	Q9BUL8	Programmed cell death protein 10	5	96.75	0	0	0	0	0	0
PDCD2	PDCD2_HUMAN	Q16342	Programmed cell death protein 2	4	99.58	4	99.58	0	0	2	99.44
PDCD5	PDCD5_HUMAN	O14737	Programmed cell death protein 5	0	0	4	99.58	5	99.58	2	99.58
PDCD6IP	PDC6I_HUMAN	Q8WUM4	Programmed cell death 6-interacting protein	35	99.58	15	99.58	20	99.58	16	99.58
PDIA4	PDIA4_HUMAN	P13667	Protein disulfide-isomerase A4	0	0	2	99.58	6	99.58	0	0
PDS5A	PDS5A_HUMAN	Q29RF7	Sister chromatid cohesion protein PDS5 homolog A	8	99.58	2	99.58	0	0	0	0
PDS5B	PDS5B_HUMAN	Q9NTI5	Sister chromatid cohesion protein PDS5 homolog B	2	99.58	0	0	0	0	0	0
PFDN2	PFD2_HUMAN	Q9UHV9	Prefoldin subunit 2	0	0	0	0	2	99.58	2	99.58
PFKL	K6PL_HUMAN	P17858	6-phosphofructokinase, liver type	8	99.58	0	0	0	0	5	99.58
PFKM	K6PF_HUMAN	P08237	6-phosphofructokinase, muscle type	2	99.58	0	0	0	0	2	91.42
PFKP	K6PP_HUMAN	Q01813	6-phosphofructokinase type C	11	99.58	0	0	0	0	0	0
PGD	6PGD_HUMAN	P52209	6-phosphogluconate dehydrogenase, decarboxylating	0	0	3	99.58	2	99.58	0	0
PGM1	PGM1_HUMAN	P36871	Phosphoglucomutase-1	0	0	2	99.58	2	99.58	0	0
PHC2	PHC2_HUMAN	Q8IXK0	Polyhomeotic-like protein 2	0	0	3	99.58	0	0	0	0
PICALM	PICAL_HUMAN	Q13492	Phosphatidylinositol-binding clathrin assembly protein	5	99.58	0	0	0	0	0	0
PIGB	PIGB_HUMAN	Q92521	GPI mannosyltransferase 3	0	0	0	0	2	99.58	0	0
PITHD1	PITH1_HUMAN	Q9GZP4	PITH domain-containing protein 1	6	99.58	0	0	0	0	14	99.58
PLIN3	PLIN3_HUMAN	O60664	Perilipin-3	5	99.58	2	99.58	0	0	0	0
PLK4	PLK4_HUMAN	O00444	Serine/threonine-protein kinase PLK4	0	0	2	86.93	0	0	0	0
PLS1	PLSI_HUMAN	Q14651	Plastin-1	0	0	0	0	3	99.58	0	0
PMVK	PMVK_HUMAN	Q15126	Phosphomevalonate kinase	9	99.58	0	0	3	99.58	0	0
PNO1	PNO1_HUMAN	Q9NRX1	RNA-binding protein PNO1	2	99.58	0	0	0	0	0	0
POLD1	DPOD1_HUMAN	P28340	DNA polymerase delta catalytic subunit	3	99.58	0	0	0	0	0	0
POLR1C	RPAC1_HUMAN	O15160	DNA-directed RNA polymerases I and III subunit RPAC1	2	99.58	0	0	0	0	0	0
POLR2E	RPAB1_HUMAN	P19388	DNA-directed RNA polymerases I, II, and III subunit RPABC1	4	99.58	0	0	5	99.58	0	0
POLR3C	RPC3_HUMAN	Q9BUI4	DNA-directed RNA polymerase III subunit RPC3	3	99.58	0	0	0	0	0	0
PPIL4	PPIL4_HUMAN	Q8WUA2	Peptidyl-prolyl cis-trans isomerase-like 4	27	99.58	25	99.58	10	99.58	20	99.58
PPM1G	PPM1G_HUMAN	O15355	Protein phosphatase 1G	2	99.58	2	99.58	0	0	10	99.58
PPP1CA	PP1A_HUMAN	P62136	Serine/threonine-protein phosphatase PP1-alpha catalytic sub	0	0	0	0	0	0	8	99.58
PPP1CB	PP1B_HUMAN	P62140	Serine/threonine-protein phosphatase PP1-beta catalytic subu	0	0	4	99.58	0	0	3	99.58
PPP1CC	PP1G_HUMAN	P36873	Serine/threonine-protein phosphatase PP1-gamma catalytic s	2	99.58	2	99.58	3	99.58	2	99.58

PPP1R10	PP1RA_HUMAN	Q96QC0	Serine/threonine-protein phosphatase 1 regulatory subunit 10	3	99.58	7	99.58	3	99.58	0	0
PPP2CA	PP2AA_HUMAN	P67775	Serine/threonine-protein phosphatase 2A catalytic subunit alp	2	99.58	2	99.58	2	99.58	2	99.58
PPP2R1B	2AAB_HUMAN	P30154	Serine/threonine-protein phosphatase 2A 65 kDa regulatory s	3	99.58	0	0	0	0	0	0
PPP2R5C	2A5G_HUMAN	Q13362	Serine/threonine-protein phosphatase 2A 56 kDa regulatory s	2	99.58	0	0	0	0	0	0
PPP2R5D	2A5D_HUMAN	Q14738	Serine/threonine-protein phosphatase 2A 56 kDa regulatory s	3	98.17	0	0	0	0	0	0
PRKAA1	AAPK1_HUMAN	Q13131	5'-AMP-activated protein kinase catalytic subunit alpha-1	2	99.58	0	0	0	0	0	0
PRKAG1	AAKG1_HUMAN	P54619	5'-AMP-activated protein kinase subunit gamma-1	2	99.58	0	0	0	0	0	0
PRKAR2A	KAP2_HUMAN	P13861	cAMP-dependent protein kinase type II-alpha regulatory subu	5	99.58	0	0	0	0	3	99.58
PRPF40A	PR40A_HUMAN	O75400	Pre-mRNA-processing factor 40 homolog A	2	99.58	0	0	0	0	0	0
PRPS1	PRPS1_HUMAN	P60891	Ribose-phosphate pyrophosphokinase 1	16	99.58	16	99.58	12	99.58	0	0
PRPS2	PRPS2_HUMAN	P11908	Ribose-phosphate pyrophosphokinase 2	4	99.58	4	99.58	3	99.58	0	0
PRPSAP1	KPRA_HUMAN	Q14558	Phosphoribosyl pyrophosphate synthase-associated protein 1	4	99.58	5	99.58	2	99.58	0	0
PRPSAP2	KPRB_HUMAN	O60256	Phosphoribosyl pyrophosphate synthase-associated protein 2	18	99.58	11	99.58	5	99.58	0	0
PRRC1	PRRC1_HUMAN	Q96M27	Protein PRRC1	2	99.58	7	99.58	2	99.58	5	99.58
PRRC2A	PRC2A_HUMAN	P48634	Protein PRRC2A	2	99.58	0	0	0	0	0	0
PRUNE	PRUNE_HUMAN	Q86TP1	Protein prune homolog	2	99.58	0	0	0	0	0	0
PSAT1	SERC_HUMAN	Q9Y617	Phosphoserine aminotransferase	2	99.58	4	99.58	3	99.58	2	99.58
PSMA1	PSA1_HUMAN	P25786	Proteasome subunit alpha type-1	2	99.58	4	99.58	6	99.58	3	99.58
PSMA3	PSA3_HUMAN	P25788	Proteasome subunit alpha type-3	0	0	6	99.58	6	99.58	2	99.58
PSMA4	PSA4_HUMAN	P25789	Proteasome subunit alpha type-4	4	99.58	3	99.58	8	99.58	0	0
PSMA5	PSA5_HUMAN	P28066	Proteasome subunit alpha type-5	13	99.58	5	99.58	6	99.58	0	0
PSMA7	PSA7_HUMAN	O14818	Proteasome subunit alpha type-7	2	99.58	3	99.58	6	99.58	0	0
PSMA8	PSA7L_HUMAN	Q8TAA3	Proteasome subunit alpha type-7-like	2	99.58	0	0	5	99.58	0	0
PSMB1	PSB1_HUMAN	P20618	Proteasome subunit beta type-1	0	0	7	99.58	6	99.58	0	0
PSMB2	PSB2_HUMAN	P49721	Proteasome subunit beta type-2	2	99.58	2	99.58	3	99.58	2	99.58
PSMB3	PSB3_HUMAN	P49720	Proteasome subunit beta type-3	0	0	0	0	3	99.58	0	0
PSMB4	PSB4_HUMAN	P28070	Proteasome subunit beta type-4	0	0	0	0	4	99.58	2	99.58
PSMB5	PSB5_HUMAN	P28074	Proteasome subunit beta type-5	3	99.58	7	99.58	8	99.58	0	0
PSMB7	PSB7_HUMAN	Q99436	Proteasome subunit beta type-7	0	0	2	99.58	2	99.58	0	0
PSMC4	PRS6B_HUMAN	P43686	26S protease regulatory subunit 6B	4	99.58	4	99.58	7	99.58	0	0
PSMC5	PRS8_HUMAN	P62195	26S protease regulatory subunit 8	2	99.58	2	99.58	6	99.58	0	0
PSMD1	PSMD1_HUMAN	Q99460	26S proteasome non-ATPase regulatory subunit 1	3	99.58	3	99.58	7	99.58	0	0
PSMD10	PSD10_HUMAN	O75832	26S proteasome non-ATPase regulatory subunit 10	0	0	0	0	0	0	2	99.58
PSMD11	PSD11_HUMAN	O00231	26S proteasome non-ATPase regulatory subunit 11	4	99.58	2	99.58	3	99.58	2	99.58
PSMD12	PSD12_HUMAN	O00232	26S proteasome non-ATPase regulatory subunit 12	2	99.58	3	99.58	2	99.58	0	0
PSMD13	PSD13_HUMAN	Q9UNM6	26S proteasome non-ATPase regulatory subunit 13	4	99.58	2	99.58	6	99.58	0	0
PSMD14	PSDE_HUMAN	O00487	26S proteasome non-ATPase regulatory subunit 14	2	99.58	0	0	0	0	0	0
PSMD3	PSMD3_HUMAN	O43242	26S proteasome non-ATPase regulatory subunit 3	3	99.58	5	99.58	7	99.58	0	0
PSMD4	PSMD4_HUMAN	P55036	26S proteasome non-ATPase regulatory subunit 4	2	99.58	3	99.58	0	0	7	99.58
PSMD6	PSMD6_HUMAN	Q15008	26S proteasome non-ATPase regulatory subunit 6	4	99.58	3	99.58	4	99.58	0	0
PSMD7	PSD7_HUMAN	P51665	26S proteasome non-ATPase regulatory subunit 7	2	99.58	2	99.58	0	0	0	0
PSME1	PSME1_HUMAN	Q06323	Proteasome activator complex subunit 1	2	99.58	0	0	0	0	2	98.85
PSME2	PSME2_HUMAN	Q9UL46	Proteasome activator complex subunit 2	9	99.58	0	0	0	0	3	99.58
PSME3	PSME3_HUMAN	P61289	Proteasome activator complex subunit 3	29	99.58	6	99.58	15	99.58	7	99.58
PSMF1	PSMF1_HUMAN	Q92530	Proteasome inhibitor PI31 subunit	7	99.58	0	0	2	99.58	0	0
PSMG1	PSMG1_HUMAN	O95456	Proteasome assembly chaperone 1	2	99.58	2	99.58	0	0	0	0
PTMA	PTMA_HUMAN	P06454	Prothymosin alpha;Thymosin alpha-1	5	99.58	6	99.58	7	99.58	5	99.58
PTMS	PTMS_HUMAN	P20962	Parathymosin	0	0	3	99.58	3	99.58	2	99.58
PYGB	PYGB_HUMAN	P11216	Glycogen phosphorylase, brain form	0	0	2	99.58	0	0	0	0
PYGM	PYGM_HUMAN	P11217	Glycogen phosphorylase, muscle form	0	0	2	99.58	0	0	0	0
QARS	SYQ_HUMAN	P47897	Glutamine--tRNA ligase	2	99.58	2	99.58	0	0	0	0
RAB12	RAB12_HUMAN	Q6IQ22	Ras-related protein Rab-12	2	99.58	2	99.58	2	99.58	2	99.58

RAB15	RAB15_HUMAN	P59190	Ras-related protein Rab-15	2	99.58	2	99.58	2	99.58	2	99.58
RAB1A	RAB1A_HUMAN	P62820	Ras-related protein Rab-1A	0	0	0	0	2	99.58	0	0
RAB39A	RB39A_HUMAN	Q14964	Ras-related protein Rab-39A	2	99.58	2	99.58	2	99.58	2	99.58
RAB3GAP1	RB3GP_HUMAN	Q15042	Rab3 GTPase-activating protein catalytic subunit	7	99.58	0	0	0	0	0	0
RAB3GAP2	RBGPR_HUMAN	Q9H2M9	Rab3 GTPase-activating protein non-catalytic subunit	14	99.58	0	0	0	0	0	0
RAB6A	RAB6A_HUMAN	P20340	Ras-related protein Rab-6A	2	99.58	2	99.58	2	99.58	2	99.58
RABGAP1	RBGP1_HUMAN	Q9Y3P9	Rab GTPase-activating protein 1	2	99.58	0	0	0	0	0	0
RAD23B	RD23B_HUMAN	P54727	UV excision repair protein RAD23 homolog B	0	0	2	99.58	2	99.58	0	0
RAD50	RAD50_HUMAN	Q92878	DNA repair protein RAD50	10	99.58	0	0	2	99.58	0	0
RAD51C	RA51C_HUMAN	O43502	DNA repair protein RAD51 homolog 3	2	99.58	0	0	0	0	0	0
RANBP2	RBP2_HUMAN	P49792	E3 SUMO-protein ligase RanBP2;Putative peptidyl-prolyl cis-tr	5	99.58	0	0	0	0	2	99.58
RANBP6	RNBP6_HUMAN	O60518	Ran-binding protein 6	4	99.58	3	99.58	4	99.58	2	99.58
RANBP9	RANB9_HUMAN	Q96S59	Ran-binding protein 9	0	0	2	99.58	0	0	0	0
RANGAP1	RAGP1_HUMAN	P46060	Ran GTPase-activating protein 1	17	99.58	6	99.58	10	99.58	2	99.58
RASSF6	RASF6_HUMAN	Q6ZTQ3	Ras association domain-containing protein 6	0	0	2	92.22	0	0	0	0
RBBP4	RBBP4_HUMAN	Q09028	Histone-binding protein RBBP4	0	0	0	0	0	0	8	99.58
RBBP7	RBBP7_HUMAN	Q16576	Histone-binding protein RBBP7	0	0	0	0	4	99.58	2	99.58
RBM33	RBM33_HUMAN	Q96EV2	RNA-binding protein 33	0	0	0	0	0	0	2	99.58
RBM4B	RBM4B_HUMAN	Q9BQ04	RNA-binding protein 4B	0	0	10	99.58	0	0	2	99.58
RBX1	RBX1_HUMAN	P62877	E3 ubiquitin-protein ligase RBX1	2	99.58	0	0	0	0	0	0
RFC1	RFC1_HUMAN	P35251	Folate transporter 1	5	99.58	0	0	0	0	0	0
RHOA	RHOA_HUMAN	P61586	Transforming protein RhoA	6	99.58	0	0	3	99.58	0	0
RIC8A	RIC8A_HUMAN	Q9NPQ8	Synembryn-A	10	99.58	0	0	2	99.58	0	0
RIF1	RIF1_HUMAN	Q5UIP0	Telomere-associated protein RIF1	12	99.58	0	0	3	99.12	0	0
RING1	RING1_HUMAN	Q06587	E3 ubiquitin-protein ligase RING1	0	0	9	99.58	0	0	0	0
RMND5A	RMD5A_HUMAN	Q9H871	Protein RMD5 homolog A	0	0	2	99.58	0	0	0	0
RNF2	RING2_HUMAN	Q99496	E3 ubiquitin-protein ligase RING2	0	0	41	99.58	0	0	0	0
RNF219	RN219_HUMAN	Q5W0B1	RING finger protein 219	7	99.58	12	99.58	0	0	2	99.58
RNGTT	MCE1_HUMAN	O60942	mRNA-capping enzyme;Polynucleotide 5'-triphosphatase;mRN	2	99.58	0	0	0	0	0	0
ROCK1	ROCK1_HUMAN	Q13464	Rho-associated protein kinase 1	2	99.58	0	0	0	0	0	0
RPL12	RL12_HUMAN	P30050	60S ribosomal protein L12	11	99.58	0	0	0	0	0	0
RPS6KA1	KS6A1_HUMAN	Q15418	Ribosomal protein S6 kinase alpha-1	2	99.58	0	0	0	0	0	0
RPSA	RSSA_HUMAN	P08865	40S ribosomal protein SA	7	99.58	0	0	0	0	0	0
RQCD1	RCD1_HUMAN	Q92600	Cell differentiation protein RCD1 homolog	4	99.58	0	0	0	0	0	0
RSU1	RSU1_HUMAN	Q15404	Ras suppressor protein 1	4	99.58	0	0	0	0	0	0
RYBP	RYBP_HUMAN	Q8N488	RING1 and YY1-binding protein	0	0	2	99.58	0	0	0	0
S100A11	S10AB_HUMAN	P31949	Protein S100-A11	0	0	2	99.58	2	99.58	0	0
S100A8	S10A8_HUMAN	P05109	Protein S100-A8	0	0	0	0	2	99.58	0	0
SAAL1	SAAL1_HUMAN	Q96ER3	Protein SAAL1	4	99.12	0	0	0	0	0	0
SAMHD1	SAMH1_HUMAN	Q9Y3Z3	SAM domain and HD domain-containing protein 1	2	99.58	0	0	0	0	0	0
SAR1A	SAR1A_HUMAN	Q9NR31	GTP-binding protein SAR1a	6	99.58	14	99.58	15	99.58	12	99.58
SAR1B	SAR1B_HUMAN	Q9Y6B6	GTP-binding protein SAR1b	0	0	0	0	3	99.58	0	0
SARS	SYSC_HUMAN	P49591	Serine--tRNA ligase, cytoplasmic	3	99.58	4	99.58	3	99.58	0	0
SCAF8	SCAF8_HUMAN	Q9UPN6	Protein SCAF8	4	99.58	0	0	0	0	0	0
SCYL1	NTKL_HUMAN	Q96KG9	N-terminal kinase-like protein	2	99.58	0	0	0	0	0	0
SEC13	SEC13_HUMAN	P55735	Protein SEC13 homolog	4	99.58	3	99.58	2	99.58	0	0
SEC23IP	S23IP_HUMAN	Q9Y6Y8	SEC23-interacting protein	2	99.58	0	0	0	0	0	0
SEC24C	SC24C_HUMAN	P53992	Protein transport protein Sec24C	2	99.58	0	0	0	0	0	0
SEC31A	SC31A_HUMAN	O94979	Protein transport protein Sec31A	2	99.58	0	0	0	0	0	0
SEPT11	SEP11_HUMAN	Q9NVA2	Septin-11	0	0	0	0	0	0	0	0
SERPINB12	SPB12_HUMAN	Q96P63	Serpin B12	0	0	0	0	4	99.58	0	0
SH3BGRL	SH3L1_HUMAN	O75368	SH3 domain-binding glutamic acid-rich-like protein	0	0	0	0	2	99.58	0	0

SH3GL1	SH3G1_HUMAN	Q99961	Endophilin-A2	0	0	10	99.58	0	0	0	0
SH3GLB1	SHLB1_HUMAN	Q9Y371	Endophilin-B1	8	99.58	9	99.58	5	99.58	4	99.58
SKA1	SKA1_HUMAN	Q96BD8	Spindle and kinetochore-associated protein 1	0	0	0	0	0	0	2	99.58
SKIV2L2	SK2L2_HUMAN	P42285	Superkiller viralicidic activity 2-like 2	8	99.58	0	0	2	99.58	0	0
SKP1	SKP1_HUMAN	P63208	S-phase kinase-associated protein 1	0	0	3	99.58	2	99.58	0	0
SMARCA2	SMCA2_HUMAN	P51531	Probable global transcription activator SNF2L2	10	99.58	0	0	0	0	2	99.58
SMARCA4	SMCA4_HUMAN	P51532	Transcription activator BRG1	1	99.58	0	0	0	0	0	0
SMARCA5	SMCA5_HUMAN	O60264	SWI/SNF-related matrix-associated actin-dependent regulator	0	0	0	0	3	99.58	0	0
SMARCAD1	SMRCD_HUMAN	Q9H4L7	SWI/SNF-related matrix-associated actin-dependent regulator	4	99.58	0	0	0	0	0	0
SMC1A	SMC1A_HUMAN	Q14683	Structural maintenance of chromosomes protein 1A	12	99.58	2	99.58	0	0	0	0
SMC2	SMC2_HUMAN	O95347	Structural maintenance of chromosomes protein 2	32	99.58	5	99.58	12	99.58	9	99.58
SMC3	SMC3_HUMAN	Q9UQE7	Structural maintenance of chromosomes protein 3	24	99.58	4	99.58	3	99.58	2	99.58
SMCHD1	SMHD1_HUMAN	A6NHR9	Structural maintenance of chromosomes flexible hinge domai	3	99.58	0	0	0	0	0	0
SMN2	SMN_HUMAN	Q16637	Survival motor neuron protein	7	99.58	12	99.58	0	0	19	99.58
SNX1	SNX1_HUMAN	Q13596	Sorting nexin-1	2	99.58	0	0	4	99.58	2	99.58
SNX12	SNX12_HUMAN	Q9UMY4	Sorting nexin-12	2	99.58	0	0	0	0	2	99.58
SNX2	SNX2_HUMAN	O60749	Sorting nexin-2	12	99.58	0	0	2	99.58	2	99.58
SNX3	SNX3_HUMAN	O60493	Sorting nexin-3	2	99.58	0	0	0	0	0	0
SNX4	SNX4_HUMAN	O95219	Sorting nexin-4	3	99.58	0	0	2	99.58	0	0
SNX5	SNX5_HUMAN	Q9Y5X3	Sorting nexin-5	0	0	0	0	6	99.58	0	0
SNX6	SNX6_HUMAN	Q9UNH7	Sorting nexin-6	3	99.58	2	99.58	3	99.29	0	0
SPC25	SPC25_HUMAN	Q9HBM1	Signal peptidase complex subunit 2	3	99.58	0	0	0	0	0	0
SPG20	SPG20_HUMAN	Q8N0X7	Spartin	2	99.58	0	0	0	0	0	0
SPR	SPRE_HUMAN	P35270	Sepiapterin reductase	2	99.58	0	0	0	0	0	0
SPTB	SPTB1_HUMAN	P11277	Spectrin beta chain, erythrocytic	0	0	0	0	0	0	2	99.58
SRM	SPEE_HUMAN	P19623	Spermidine synthase	0	0	2	94.12	2	96	0	0
SRR	SRR_HUMAN	Q9GZT4	Serine racemase	5	99.58	0	0	0	0	0	0
ST13	F10A1_HUMAN	P50502	Hsc70-interacting protein	0	0	0	0	8	99.58	0	0
STAG2	STAG2_HUMAN	Q8N3U4	Cohesin subunit SA-2	2	99.58	0	0	0	0	0	0
STAT1	STAT1_HUMAN	P42224	Signal transducer and activator of transcription 1-alpha/beta	2	99.58	0	0	0	0	0	0
STAT3	STAT3_HUMAN	P40763	Signal transducer and activator of transcription 3	15	99.58	0	0	0	0	0	0
STIP1	STIP1_HUMAN	P31948	Stress-induced-phosphoprotein 1	10	99.58	13	99.58	13	99.58	2	99.58
STMN2	STMN2_HUMAN	Q93045	Stathmin-2	2	99.58	2	99.58	2	99.58	2	99.58
STRAP	STRAP_HUMAN	Q9Y3F4	Serine-threonine kinase receptor-associated protein	13	99.58	9	99.58	4	99.58	11	99.58
STRN3	STRN3_HUMAN	Q13033	Striatin-3	0	0	0	0	5	99.58	0	0
SUGT1	SUGT1_HUMAN	Q9Y2Z0	Suppressor of G2 allele of SKP1 homolog	2	99.58	0	0	0	0	0	0
SULT1A1	ST1A1_HUMAN	P50225	Sulfotransferase 1A1	4	95.85	0	0	0	0	0	0
SYAP1	SYAP1_HUMAN	Q96A49	Synapse-associated protein 1	10	99.58	20	99.58	10	99.58	22	99.58
SYMPK	SYMPK_HUMAN	Q92797	Symplekin	6	99.58	0	0	0	0	0	0
TAB1	TAB1_HUMAN	Q15750	TGF-beta-activated kinase 1 and MAP3K7-binding protein 1	6	99.58	4	99.58	3	99.58	7	99.58
TAF4	TAF4_HUMAN	O00268	Transcription initiation factor TFIID subunit 4	0	0	0	0	4	99.58	0	0
TAGLN3	TAGL3_HUMAN	Q9UI15	Transgelin-3	0	0	2	99.58	0	0	0	0
TALDO1	TALDO_HUMAN	P37837	Transaldolase	2	99.58	4	99.58	7	99.58	0	0
TARS	SYTC_HUMAN	P26639	Threonine--tRNA ligase, cytoplasmic	0	0	0	0	3	99.58	0	0
TBC1D15	TBC15_HUMAN	Q8TC07	TBC1 domain family member 15	4	99.58	0	0	2	99.58	0	0
TBC1D4	TBCD4_HUMAN	O60343	TBC1 domain family member 4	5	99.58	2	99.47	0	0	0	0
TBCB	TBCB_HUMAN	Q99426	Tubulin-folding cofactor B	2	99.58	2	99.58	0	0	0	0
TELO2	TELO2_HUMAN	Q9Y4R8	Telomere length regulation protein TEL2 homolog	3	99.58	0	0	0	0	0	0
TFIP11	TFP11_HUMAN	Q9UBB9	Tuftelin-interacting protein 11	5	99.58	0	0	0	0	0	0
TH1L	NELFD_HUMAN	Q8IXH7	Negative elongation factor C/D	8	99.58	0	0	0	0	2	90.13
THOC2	THOC2_HUMAN	Q8NI27	THO complex subunit 2	3	99.58	0	0	0	0	0	0
TIMM13	TIM13_HUMAN	Q9Y5L4	Mitochondrial import inner membrane translocase subunit Ti	5	99.58	6	99.58	7	99.58	5	99.58

TIMM8B	TIM8B_HUMAN	Q9Y5J9	Mitochondrial import inner membrane translocase subunit Ti	0	0	0	0	2	99.58	2	99.58
TIMM9	TIM9_HUMAN	Q9Y5J7	Mitochondrial import inner membrane translocase subunit Ti	0	0	4	99.58	0	0	0	0
TIPRL	TIPRL_HUMAN	O75663	TIP41-like protein	5	99.58	0	0	0	0	0	0
TLE1	TLE1_HUMAN	Q04724	Transducin-like enhancer protein 1	2	99.58	0	0	0	0	0	0
TLE2	TLE2_HUMAN	Q04725	Transducin-like enhancer protein 2	2	99.58	0	0	0	0	0	0
TMSB10	TYB10_HUMAN	P63313	Thymosin beta-10	2	99.58	5	99.58	2	99.58	2	99.58
TMSB15A	TB15A_HUMAN	P0CG34	Thymosin beta-15A	0	0	2	99.58	0	0	0	0
TNPO1	TNPO1_HUMAN	Q92973	Transportin-1	27	99.58	7	99.58	11	99.58	8	99.58
TNPO3	TNPO3_HUMAN	Q9Y5L0	Transportin-3	4	99.58	0	0	0	0	0	0
TPD52L2	TPD54_HUMAN	O43399	Tumor protein D54	11	99.58	6	99.58	4	99.58	3	99.58
TPP1	TPP1_HUMAN	O14773	Alpha-tocopherol transfer protein	2	99.58	6	99.58	5	99.58	2	99.58
TPR	TPR_HUMAN	P12270	Nucleoprotein TPR	5	99.58	2	99.58	2	99.58	0	0
TPT1	TCTP_HUMAN	P13693	Translationally-controlled tumor protein	0	0	0	0	3	99.58	0	0
TRIM25	TRI25_HUMAN	Q14258	E3 ubiquitin/ISG15 ligase TRIM25	0	0	2	99.58	0	0	0	0
TRIM28	TIF1B_HUMAN	Q13263	Transcription intermediary factor 1-beta	27	99.58	0	0	0	0	0	0
TRIM33	TRI33_HUMAN	Q9UPN9	E3 ubiquitin-protein ligase TRIM33	2	99.58	0	0	0	0	0	0
TRIP13	PCH2_HUMAN	Q15645	Pachytene checkpoint protein 2 homolog	19	99.58	4	99.58	2	99.58	0	0
TSN	TSN_HUMAN	Q15631	Translin	2	99.58	0	0	0	0	0	0
TSNAX	TSNAX_HUMAN	Q99598	Translin-associated protein X	2	99.58	0	0	0	0	0	0
TTC4	TTC4_HUMAN	O95801	Tetratricopeptide repeat protein 4	7	99.58	0	0	0	0	0	0
TUBAL3	TBAL3_HUMAN	A6NHL2	Tubulin alpha chain-like 3	2	99.58	0	0	0	0	2	99.58
TUBB3	TBB3_HUMAN	Q13509	Tubulin beta-3 chain	2	99.58	4	99.58	4	99.58	4	99.58
TUBG1	TBG1_HUMAN	P23258	Tubulin gamma-1 chain	8	99.58	0	0	0	0	0	0
TUBGCP2	GCP2_HUMAN	Q9BSJ2	Gamma-tubulin complex component 2	4	99.58	2	99.58	0	0	0	0
TXNDC17	TXD17_HUMAN	Q9BRA2	Thioredoxin domain-containing protein 17	0	0	0	0	4	99.58	0	0
TXNDC5	TXND5_HUMAN	Q8NBS9	Thioredoxin domain-containing protein 5	0	0	2	80	0	0	0	0
TYMS	TYSY_HUMAN	P04818	Thymidylate synthase	0	0	0	0	2	99.58	0	0
UBA5	UBA5_HUMAN	Q9GZZ9	Ubiquitin-like modifier-activating enzyme 5	9	99.58	0	0	0	0	0	0
UBE2D2	UB2D2_HUMAN	P62837	Ubiquitin-conjugating enzyme E2 D2	2	99.58	0	0	0	0	0	0
UBE2E1	UB2E1_HUMAN	P51965	Ubiquitin-conjugating enzyme E2 E1	4	99.58	0	0	3	99.58	2	99.58
UBE2K	UBE2K_HUMAN	P61086	Ubiquitin-conjugating enzyme E2 K	6	99.58	3	99.58	4	99.58	6	99.58
UBE2M	UBC12_HUMAN	P61081	NEDD8-conjugating enzyme Ubc12	10	99.58	8	99.58	6	99.58	7	99.58
UBE2N	UBE2N_HUMAN	P61088	Ubiquitin-conjugating enzyme E2 N	9	99.58	6	99.58	6	99.58	8	99.58
UBE2O	UBE2O_HUMAN	Q9C0C9	Ubiquitin-conjugating enzyme E2 O	4	99.58	2	99.58	4	99.58	0	0
UBE2S	UBE2S_HUMAN	Q16763	Ubiquitin-conjugating enzyme E2 S	2	99.58	0	0	0	0	0	0
UBE2V1	UB2V1_HUMAN	Q13404	Ubiquitin-conjugating enzyme E2 variant 1	0	0	0	0	2	99.58	0	0
UBE2Z	UBE2Z_HUMAN	Q9H832	Ubiquitin-conjugating enzyme E2 Z	4	99.58	0	0	2	99.58	2	99.58
UBL4A	UBL4A_HUMAN	P11441	Ubiquitin-like protein 4A	3	99.58	0	0	0	0	0	0
UBR4	UBR4_HUMAN	Q5T4S7	E3 ubiquitin-protein ligase UBR4	5	99.58	0	0	0	0	0	0
UCHL5	UCHL5_HUMAN	Q9Y5K5	Ubiquitin carboxyl-terminal hydrolase isozyme L5	6	99.58	0	0	4	99.58	0	0
UMPS	UMPS_HUMAN	P11172	Uridine 5'-monophosphate synthase;Orotate phosphoribosylt	3	99.58	0	0	0	0	0	0
UNC45A	UN45A_HUMAN	Q9H3U1	Protein unc-45 homolog A	3	99.58	0	0	0	0	0	0
UNC5D	UNC5D_HUMAN	Q6UXZ4	Netrin receptor UNC5D	2	99.58	0	0	0	0	0	0
USO1	USO1_HUMAN	O60763	General vesicular transport factor p115	35	99.58	5	99.58	11	99.58	14	99.58
USP10	UBP10_HUMAN	Q14694	Ubiquitin carboxyl-terminal hydrolase 32	5	99.58	0	0	0	0	0	0
USP11	UBP11_HUMAN	P51784	Ubiquitin carboxyl-terminal hydrolase 11	3	99.58	0	0	0	0	0	0
USP15	UBP15_HUMAN	Q9Y4E8	Ubiquitin carboxyl-terminal hydrolase 15	2	99.58	2	99.58	2	99.58	0	0
USP22	UBP22_HUMAN	Q9UPT9	Ubiquitin carboxyl-terminal hydrolase 22	2	99.58	0	0	0	0	0	0
USP24	UBP24_HUMAN	Q9UPU5	Ubiquitin carboxyl-terminal hydrolase 24	5	99.58	0	0	0	0	0	0
USP48	UBP48_HUMAN	Q86UV5	Ubiquitin carboxyl-terminal hydrolase 48	2	99.58	0	0	0	0	0	0
USP7	UBP7_HUMAN	Q93009	Ubiquitin carboxyl-terminal hydrolase 7	2	99.58	6	99.58	0	0	0	0
USP9X	USP9X_HUMAN	Q93008	Probable ubiquitin carboxyl-terminal hydrolase FAF-X	2	99.58	0	0	0	0	0	0

VAT1	VAT1_HUMAN	Q99536	Chromaffin granule amine transporter	8	99.58	7	99.58	3	99.58	2	94.17
VCL	VINC_HUMAN	P18206	Vinculin	2	99.58	2	99.58	0	0	0	0
VPRBP	VPRBP_HUMAN	Q9Y4B6	Protein VPRBP	2	99.58	0	0	0	0	0	0
VPS35	VPS35_HUMAN	Q96QK1	Vacuolar protein sorting-associated protein 35	0	0	0	0	2	99.58	0	0
VTA1	VTA1_HUMAN	Q9NP79	Vacuolar protein sorting-associated protein VTA1 homolog	5	99.58	0	0	0	0	0	0
WDR26	WDR26_HUMAN	Q9H7D7	WD repeat-containing protein 26	2	99.58	3	99.44	0	0	0	0
WDR44	WDR44_HUMAN	Q5JSH3	WD repeat-containing protein 44	0	0	6	99.58	0	0	2	99.58
WDR5	WDR5_HUMAN	P61964	WD repeat-containing protein 5	0	0	2	99.58	0	0	0	0
WRNIP1	WRIP1_HUMAN	Q96S55	ATPase WRNIP1	8	99.58	0	0	0	0	0	0
XPNPEP1	XPP1_HUMAN	Q9NQW7	Xaa-Pro aminopeptidase 1	0	0	0	0	2	99.58	0	0
XPO5	XPO5_HUMAN	Q9HAV4	Exportin-5	40	99.58	5	99.58	7	99.58	8	99.58
XRCC6	XRCC6_HUMAN	P12956	X-ray repair cross-complementing protein 6	17	99.58	0	0	19	99.58	0	0
YAF2	YAF2_HUMAN	Q8IY57	YY1-associated factor 2	0	0	3	99.58	0	0	0	0
YKT6	YKT6_HUMAN	O15498	Synaptobrevin homolog YKT6	14	99.58	3	99.58	3	99.58	3	99.58
YWHAG	1433G_HUMAN	P61981	14-3-3 protein gamma;14-3-3 protein gamma, N-terminally pr	8	99.58	8	99.58	4	99.58	4	99.58
YWHAH	1433F_HUMAN	Q04917	14-3-3 protein eta	4	99.58	4	99.58	0	0	4	99.58
ZC3HC1	NIPA_HUMAN	Q86WB0	Nuclear-interacting partner of ALK	4	99.58	0	0	0	0	0	0
ZNF24	ZNF24_HUMAN	P17028	Zinc finger protein 24	0	0	6	99.58	0	0	0	0
ZNF570	ZN570_HUMAN	Q96NI8	Zinc finger protein 570	0	0	2	99.58	0	0	0	0
ZRANB2	ZRAB2_HUMAN	O95218	Zinc finger Ran-binding domain-containing protein 2	2	99.58	0	0	0	0	0	0
ZW10	ZW10_HUMAN	O43264	Centromere/kinetochore protein zw10 homolog	2	99.58	2	99.58	2	99.58	0	0

Note: Peptide counts reflect the relative abundance of the protein within the purification.
