# Supplementary material for: Efficient prediction of human protein-protein interactions at a global scale
Source: BMC Bioinformatics. 2014 Dec 10;15(1):383. doi: 10.1186/s12859-014-0383-1 (PMC4272565; doi:10.1186/s12859-014-0383-1)
Supplement: Additional file 5: — List of interactions for fibroblast growth factors (FGFs) and cyclin-dependent kinases (CDKs) and FGF regulators and CDK inhibitor/activators. *are interactors that have not been previously reported. [file 12859_2014_383_MOESM5_ESM.pdf]

The interactions of fibroblast growth factors (FGFs) with fibroblast growth factor receptors (FGFRs).	
**FGF**	**FGFR**
P61328	
P31371	
Q9NSA1	
Q9GZV9	
P21781	P11362*, P22607*, P21802
O43320	
O95750	P22455
O15520	P21802
P11487	
P55075	
P12034	
P10767	
P08620	
P09038	P11362, P22607*, P21802*
Q92914	
Q92915	
O76093	
Q92913	
P05230	P11362, P22607, P21802, P22455
O60258	
Q9HCT0	
Q9NP95	

The interactions of cyclin-dependent kinases (CDKs) with regulators of cyclin-dependent kinases.	
**CDK**	**Regulator of CDK**
Q8IZL9	
Q96Q40	P63104*, P31946*
O94921	P63104*, P31946
P50613	
Q9UQ88	P31946*, P63104*
Q00526	P31946*, P49918*, P42773*, Q16667, P46527, P38936, P55273*, Q13319*, P63104*, Q15078*, Q96SZ6*, Q8IWU2*, O15392*
Q00534	O15392*, P42772*, P46527, P49918*, P42773, P38936, Q8N726, P55273
Q00537	P63104, P31946*, Q15078*
Q00536	P63104, P31946*, Q15078
Q00535	O15392*, P38936*, P46527, P31946*, Q13319, Q96SZ6, Q8IWU2, P63104*, Q15078, Q16667*
Q9BWU1	O75448*, P42772*, Q15648*, Q93074*, Q9UHV7*, O75586*
Q14004	
Q9NYV4	
Q15131	P31946*, P63104*
P50750	Q15078
P06493	P46527*, Q13319*, P63104*, Q96SZ6*, P38936, Q8IWU2*, P31946*, Q16667, O15392, Q15078*
P11802	P38936, P42773, Q8N726, P55273, P42772, P46527, P49918, O15392, P42771*
P21127	P31946, P63104*
P24941	P38936, P31946*, P63104*, P46527, Q16667, Q8IWU2*, P49918*, Q15078*, Q96SZ6*, Q13319*, O15392
Q07002	P31946*, P63104*, Q15078*
P49336	O75586, P42771*, Q93074, Q15648, P42772, Q9UHV7, O75448
