# Supplementary material for: Efficient prediction of human protein-protein interactions at a global scale
Source: BMC Bioinformatics. 2014 Dec 10;15(1):383. doi: 10.1186/s12859-014-0383-1 (PMC4272565; doi:10.1186/s12859-014-0383-1)
Supplement: Additional file 6: — List of hub proteins in H. sapiens ranked by the number of neighbors for each protein. [file 12859_2014_383_MOESM6_ESM.pdf]

Protein	# of Neighbours	# of Known Neighbours	# of Novel Neighbours
Q156A1	3773	0	3773
P02811	1174	0	1174
Q15072	1018	0	1018
P51522	1004	2	1002
Q5EBM4	938	0	938
Q96CX3	937	0	937
Q8N782	895	0	895
Q3ZCT1	884	0	884
Q8N3J9	875	0	875
P84022	836	184	652
Q96N58	797	0	797
Q96SK3	759	1	758
Q9H963	743	0	743
Q14585	741	0	741
P31946	722	127	595
P63104	721	135	586
P17039	721	0	721
Q04917	719	60	659
P27348	716	114	602
P61981	713	245	468
P31947	710	108	602
Q8NB50	706	0	706
Q8TAQ5	694	0	694
Q15796	672	168	504
P62258	671	70	601
Q8TF20	671	0	671
P12931	670	223	447
Q99717	669	37	632
Q15797	661	115	546
Q6ZR52	645	0	645
Q15940	634	0	634
P06241	624	153	471
P07948	624	100	524
P06239	615	103	512
O15198	611	105	506
Q6AZW8	604	0	604
Q6ZTB9	600	0	600
P08631	597	53	544
P09769	594	18	576
A8MUV8	590	0	590

P07947	584	29	555
Q6P3V2	583	0	583
Q52M93	582	1	581
Q9Y2P7	581	0	581
Q9H7R5	578	0	578
A6NN14	568	0	568
P51451	554	12	542
Q6ZN57	545	1	544
P04637	527	263	264
Q8N972	520	0	520
Q3KP31	516	0	516
A8MXY4	504	0	504
O43345	495	1	494
Q05481	494	1	493
P17252	493	173	320
P06493	481	120	361
P05771	477	69	408
P28482	470	168	302
Q9UII5	468	2	466
Q5SXM1	467	0	467
P27361	465	116	349
Q09472	464	219	245
Q9P2J8	460	0	460
Q92793	454	219	235
P68133	452	111	341
P63267	451	2	449
P63261	451	46	405
P60709	451	106	345
Q562R1	450	0	450
P68032	450	32	418
Q9BYX7	449	0	449
P62736	449	15	434
P10275	446	158	288
Q13485	445	154	291
P03372	437	203	234
P24941	436	95	341
Q86YE8	435	0	435
Q00526	428	15	413
P05129	425	47	378
P38398	424	161	263
P17017	419	0	419

Q6ZNH5	395	0	395
Q15834	392	125	267
P06400	386	145	241
Q13547	382	151	231
P62993	372	217	155
P17030	371	0	371
Q00535	367	49	318
Q92769	364	91	273
Q8TBZ8	364	0	364
P17612	357	158	199
P22694	355	4	351
Q99750	352	101	251
O15379	351	68	283
P04280	351	0	351
P22612	350	1	349
Q8N7K0	340	0	340
Q9BYQ5	339	0	339
Q9BYQ8	338	0	338
Q9BYQ6	338	0	338
Q9BYR0	338	0	338
Q9BYR3	338	0	338
Q9BQ66	338	74	264
A8MXZ3	338	0	338
Q9BYR4	338	0	338
Q9BYR5	338	0	338
Q14591	333	0	333
Q9BYQ7	332	0	332
Q15929	331	0	331
Q9BYQ9	331	0	331
Q9BYR2	331	0	331
A8MTL4	331	0	331
P63000	329	101	228
Q6ZN19	326	0	326
P61163	325	6	319
Q96IR2	324	0	324
Q13588	322	11	311
Q13263	319	26	293
P84095	317	18	299
Q9NRR5	316	143	173
P68400	313	175	138
P19784	311	76	235

P00533	310	159	151
P04281	308	0	308
Q8TC17	307	0	307
Q9BYD9	305	0	305
P60763	304	5	299
Q6VMQ6	303	61	242
P15622	303	11	292
O14964	300	89	211
P36897	299	147	152
Q08AN1	297	0	297
Q9BX82	297	0	297
O43830	296	0	296
Q12933	296	148	148
Q01844	295	115	180
Q9NXT0	294	0	294
P63167	294	64	230
Q96FJ2	294	11	283
P15153	292	17	275
Q9NSJ1	292	0	292
Q8N8L2	291	0	291
Q9Y4K3	291	105	186
P24928	288	84	204
Q96JF6	286	1	285
Q15973	283	0	283
P08670	280	111	169
B1ANC4	280	0	280
Q9Y6R6	279	0	279
P54253	278	146	132
P17038	271	0	271
P31749	271	121	150
P63165	270	66	204
P35222	268	146	122
Q8IYB9	267	0	267
O75290	266	0	266
P31751	266	20	246
P61956	265	29	236
P61586	263	84	179
Q7L3S4	263	0	263
Q96I27	263	0	263
P60953	261	96	165
Q9Y2A4	261	0	261

Q05516	260	83	177
Q6EEV6	259	74	185
Q96GE5	258	0	258
Q5T3J3	257	95	162
P56524	255	49	206
Q6ZNG1	255	0	255
Q5UIP0	254	89	165
P08134	253	6	247
P17081	251	17	234
O14641	249	50	199
P04150	249	99	150
Q86YH7	248	0	248
P52743	247	0	247
P49910	244	14	230
Q494X3	244	0	244
Q13432	243	79	164
O95129	242	0	242
P51504	242	0	242
P42025	241	3	238
P11142	241	66	175
P55854	240	20	220
O95132	240	0	240
Q4V348	240	0	240
Q86UE3	239	0	239
Q9Y243	239	5	234
Q6ZNA1	239	0	239
P14136	238	23	215
P08107	238	7	231
P54652	238	4	234
O15162	237	71	166
Q8N8Z8	237	0	237
Q15505	236	0	236
P35789	236	0	236
P34931	235	0	235
P16415	235	0	235
P54257	233	56	177
P17024	233	2	231
Q6N045	232	0	232

… (continued)
