# Supplementary material for: Efficient prediction of human protein-protein interactions at a global scale
Source: BMC Bioinformatics. 2014 Dec 10;15(1):383. doi: 10.1186/s12859-014-0383-1 (PMC4272565; doi:10.1186/s12859-014-0383-1)
Supplement: Additional file 7: — List of betweenness centrality proteins in H. sapiens ranked by highest betweenness centrality measure. [file 12859_2014_383_MOESM7_ESM.pdf]

Protein	Betweenness Centrality
Q156A1	52021233.9
P02811	4789053.495
P84022	2020362.837
P04637	1512443.906
Q01844	1502425.216
Q9NRR5	1308161.975
Q04917	1081315.053
P31946	1080073.719
P63104	1074074.893
P61981	1066679.146
P27348	1055129.145
P31947	1020967.678
Q15796	997823.9529
P12931	968522.5928
P62258	936053.7736
P06493	924389.88
Q15072	905510.9073
P17252	896109.4058
Q99717	889536.9031
P51522	856438.3568
Q15797	844447.963
Q15834	837591.8195
P07948	836470.2039
P00533	832163.2601
P05771	810413.1646
O15198	778728.1743
Q12933	777884.9436
P24941	776428.9543
P36897	773216.1677
Q00526	758712.9459
P38398	744745.8584
P06241	739865.7993
Q13485	735311.963
P06239	729641.8094
Q96CX3	680181.1306
Q99750	679463.9587
P08631	676504.3603
P09769	654735.3606
Q9Y4K3	631413.5406
Q5EBM4	629314.483

P05129	626372.9027
P07947	622006.694
P68400	615077.4797
P19784	610290.9836
P51451	602386.2418
P03372	599551.7374
P17612	592693.4733
P22694	588460.7496
P35222	577673.2005
P12956	568535.4678
P22612	568162.7053
Q00535	562788.8777
Q3ZCT1	539780.0972
P42574	532473.4279
Q8N782	526007.7482
P21246	499711.1552
P28482	489364.9159
Q6VMQ6	475817.4288
Q8N3J9	473968.6411
Q9UMX0	470085.947
P04278	469193.0433
P27361	462853.3575
Q13432	441731.4494
P06400	439611.8266
P68133	432407.786
Q8TAQ5	417345.8298
P14923	415283.5588
P68032	415173.6732
P60709	414821.7951
P63261	414821.7951
P63267	414821.7951
P23743	414390.8148
Q562R1	411180.115
P62736	409673.9766
Q9BYX7	408689.39
P05556	407941.0009
P12757	406611.3195
Q9HAU4	405209.0784
P62993	403615.1116
P08670	401341.9222
P26641	398500.007

P78352	383536.6502
Q15047	374974.569
P29350	369556.5808
Q14585	359840.7075
P46379	354102.8926
P63279	351720.3849
P14136	347096.1649
Q96N58	346680.7353
P05067	346670.8886
Q06124	346652.7926
P31749	342284.2401
Q8NER5	341501.3381
Q13547	339785.8519
P56537	325428.6134
O95777	323741.9206
P63000	323535.8183
Q15645	322672.9701
P63165	322653.9224
P31751	321530.7232
P10415	320624.7273
O15360	317689.1556
P02751	317480.6509
P54257	315131.2275
P61956	315078.9184
Q7L5N1	310155.6736
P04280	310094.6182
Q13588	309839.1937
P68104	306817.9115
Q05639	306817.9115
Q5VTE0	306817.9115
Q14050	305987.7037
P08195	305488.2983
Q96SK3	305018.1426
P04281	304859.9121
P08123	302179.7905
Q6EEV6	300214.0731
Q05655	299604.8998
Q9HCE7	299214.8227
Q92769	296664.9502
P84095	295983.9751
P00519	293204.22

Q9UNE7	287977.0422
Q14055	286652.2885
P40337	285680.4777
Q92793	285540.8038
Q13873	285321.1953
Q5UIP0	284660.9884
Q07954	284568.7354
Q99972	281665.9663
Q09472	280564.4606
P17039	279781.5959
P63208	278163.6834
P40763	276376.1561
O15379	275066.6071
Q07817	273685.1291
P10275	272804.2886
P55854	272671.3297
O75400	270424.6946
P56539	267258.7326
P63167	266021.0142
Q96FJ2	266021.0142
Q9Y243	265311.7281
O14745	264454.6557
Q06609	263866.3293
Q8TC17	262492.4542
Q92796	262133.5964
Q15287	261679.1612
P01106	260629.7906
P60763	259338.3746
Q9H963	257228.1751
P04049	256666.8299
Q9H7R5	256504.3926
Q03135	254665.5215
Q16665	252528.0956
Q9NY93	252510.8494
P22607	249439.2376
Q8NB50	247371.4905
P15153	245462.5229
Q8TF20	245224.6769
P01112	244979.9036
O43586	244829.4337
Q14721	238713.0272

P61163	236059.9985
P21673	236009.3696
P67870	234386.0056
Q5T3J3	233453.1176
P17661	230641.411
P11142	229619.9209
P36873	228331.2813
Q12959	228114.5168
P01042	228091.1253
P02647	227794.9739
P09958	227183.2269
Q12873	226141.6866
Q8N726	226070.1162
P08107	223197.6805
P54652	223197.6805
Q6ZN57	222390.0087
P36575	221710.3069
P21333	221273.0424
Q99689	220979.2807
P62136	220717.7876
Q6AZW8	218180.2579
P24928	217615.0626
Q9NZR2	217034.5122
O14641	216804.2392
Q16539	216133.3308
P34931	215554.7688
P20023	210689.6985
Q9BYD9	207897.8158
Q9Y6T7	207161.6667
P54253	207067.6117
O15162	205845.8895
Q9Y3C5	205781.1239
Q9NRY7	203812.8736
O15287	203196.2827
Q15700	203070.0635
P62140	202601.377
P01111	201076.925

… (continued)
