# Supplementary material for: Efficient prediction of human protein-protein interactions at a global scale
Source: BMC Bioinformatics. 2014 Dec 10;15(1):383. doi: 10.1186/s12859-014-0383-1 (PMC4272565; doi:10.1186/s12859-014-0383-1)
Supplement: Additional file 8: — List of protein mutations associated with resistance to breast cancer therapeutics doxorubicin and Trastuzumab. [file 12859_2014_383_MOESM8_ESM.pdf]

Mutation Name	Protein Name	Mutation
P04637a	P04637	First 204 amino acids only
P04637b	P04637	Codon 249, R replaced with G
P04637c	P04637	Codons 217-221 deleted
P04637d	P04637	Codon 248, R replaced with Q
P04626a	P04626	First 611 amino acids only
P04626b	P04626	First 648 amino acids only
