# Supplementary material for: Efficient prediction of human protein-protein interactions at a global scale
Source: BMC Bioinformatics. 2014 Dec 10;15(1):383. doi: 10.1186/s12859-014-0383-1 (PMC4272565; doi:10.1186/s12859-014-0383-1)
Supplement: Additional file 9: — List of proteins from the acute phase response pathway, complement pathway and glucocorticoids receptor pathway. [file 12859_2014_383_MOESM9_ESM.pdf]

A1L4G7
A5PL27
B1AMY1
B3KNT3
B5BUQ7
C9IYG8
C9IZP8
C9J1C7
C9J1D9
C9JC72
C9JHD2
C9JU00
C9JV77
D3DUU2
D5M8Q2
D6REL8
E7EMZ6
E7ERA4
E7ET33
E9PD65
E9PDS4
E9PER2
E9PF72
E9PI80
E9PJN2
E9PK97
O00187
O14543
O43524
O60244
O60424
O75051
O75376
O95608
P00736
P00738
P00746
P00749
P00751
P01009
P01011
P01019
P01023
P01024
P01031
P01100
P01160

P01375
P01579
P01584
P02735
P02741
P02743
P02749
P02760
P02763
P02766
P02790
P02818
P04049
P04083
P04141
P04150
P04196
P05112
P05113
P05121
P05231
P05412
P05546
P06401
P06681
P07550
P07900
P08603
P08700
P08887
P09429
P09601
P10145
P10147
P10415
P10643
P11226
P11684
P12314
P13500
P13501
P15529
P16581
P17676
P18510
P19320
P19419

P19875
P21730
P22301
P23458
P25963
P27352
P28562
P29353
P31749
P32241
P34932
P35225
P35228
P38936
P40424
P42224
P42345
P45983
P48552
P48736
P49715
P49918
P51606
P51617
P51692
P52333
P61201
P61978
P62993
P63000
P63165
P63279
P81172
Q00403
Q02790
Q04206
Q04917
Q06AH7
Q06DL9
Q07817
Q09472
Q13233
Q13451
Q13514
Q13546
Q14551
Q14624

Q14978
Q14UF5
Q15185
Q15596
Q15628
Q15648
Q15750
Q15788
Q16334
Q16581
Q16822
Q19MP7
Q4FCH6
Q506Q0
Q5JNX2
Q5SBK4
Q5T7S2
Q6I9T4
Q6LDG4
Q6LDI0
Q6LDP0
Q6LE88
Q6NWP6
Q6PIW7
Q6QR78
Q86SI0
Q8TCE8
Q8TCF0
Q8TD58
Q92831
Q96DZ4
Q99557
Q99558
Q99576
Q99616
Q99650
Q99683
Q99836
Q99893
Q99933
Q9BQ95
Q9BXH2
Q9BYG6
Q9NZ70
Q9UGE8
Q9UGI4
Q9UMI2

Q9Y478
Q9Y6K9
Q9Y6Q9
