# Supplementary material for: Efficient prediction of human protein-protein interactions at a global scale
Source: BMC Bioinformatics. 2014 Dec 10;15(1):383. doi: 10.1186/s12859-014-0383-1 (PMC4272565; doi:10.1186/s12859-014-0383-1)
Supplement: Additional file 10: — List of paracliques identified in this study including the member proteins for the paraclique, interactions in the paraclique as well as GO tags for molecular function and biological process shared by the members of the paraclique with P-value 1E-06. [file 12859_2014_383_MOESM10_ESM.pdf]

Paraclique 1 (29 members)
O14514
O43707
P00533
P02811
P07948
P12814
P12830
P15311
P22223
P35221
P35609
P42025
P60709
P61163
P62736
P63261
P63267
P68032
P68133
P78352
Q08043
Q12959
Q156A1
Q15700
Q562R1
Q92796
Q96QZ7
Q9BYD9
Q9BYX7

O14514	P02811	novel
O14514	P78352	known
O14514	Q12959	novel
O14514	Q156A1	novel
O14514	Q15700	novel
O14514	Q92796	novel
O14514	Q96QZ7	known
O43707	P02811	novel
O43707	P12814	novel
O43707	P12830	novel
O43707	P35221	novel
O43707	P35609	novel
O43707	P42025	novel
O43707	P60709	novel
O43707	P61163	novel
O43707	P62736	novel
O43707	P63261	novel
O43707	P63267	novel
O43707	P68032	known
O43707	P68133	novel
O43707	P78352	novel
O43707	Q08043	known
O43707	Q12959	novel
O43707	Q156A1	novel
O43707	Q15700	novel
O43707	Q562R1	novel
O43707	Q92796	novel
O43707	Q96QZ7	known
O43707	Q9BYD9	novel
O43707	Q9BYX7	novel
P00533	P02811	novel
P00533	P07948	known
P00533	P12830	known
P00533	P15311	known
P00533	P22223	novel
P00533	P42025	novel
P00533	P60709	novel
P00533	P61163	novel
P00533	P62736	novel
P00533	P63261	novel

P00533	P63267	novel
P00533	P68032	novel
P00533	P68133	known
P00533	P78352	novel
P00533	Q12959	novel
P00533	Q156A1	novel
P00533	Q15700	novel
P00533	Q562R1	novel
P00533	Q92796	novel
P00533	Q9BYD9	novel
P00533	Q9BYX7	novel
P02811	P07948	novel
P02811	P12814	novel
P02811	P12830	novel
P02811	P35609	novel
P02811	P42025	novel
P02811	P60709	novel
P02811	P61163	novel
P02811	P62736	novel
P02811	P63261	novel
P02811	P63267	novel
P02811	P68032	novel
P02811	P68133	novel
P02811	P78352	novel
P02811	Q08043	novel
P02811	Q12959	novel
P02811	Q156A1	novel
P02811	Q15700	novel
P02811	Q562R1	novel
P02811	Q92796	novel
P02811	Q96QZ7	novel
P02811	Q9BYD9	novel
P02811	Q9BYX7	novel
P07948	P12830	novel
P07948	P15311	novel
P07948	P42025	novel
P07948	P60709	known
P07948	P61163	novel
P07948	P62736	novel
P07948	P63261	novel
P07948	P63267	novel
P07948	P68032	novel
P07948	P68133	novel
P07948	P78352	known
P07948	Q12959	novel
P07948	Q156A1	novel
P07948	Q15700	novel
P07948	Q562R1	novel
P07948	Q92796	novel
P07948	Q9BYD9	novel
P07948	Q9BYX7	novel
P12814	P12830	known
P12814	P22223	novel
P12814	P35221	known
P12814	P35609	novel
P12814	P42025	novel
P12814	P60709	novel
P12814	P61163	novel
P12814	P62736	novel
P12814	P63261	novel
P12814	P63267	novel
P12814	P68032	novel
P12814	P68133	novel
P12814	P78352	novel
P12814	Q08043	novel
P12814	Q12959	novel
P12814	Q156A1	novel
P12814	Q15700	novel
P12814	Q562R1	novel
P12814	Q92796	novel

P12814	Q96QZ7	novel
P12814	Q9BYD9	novel
P12814	Q9BYX7	novel
P12830	P15311	known
P12830	P22223	known
P12830	P35221	known
P12830	P35609	novel
P12830	P42025	novel
P12830	P60709	novel
P12830	P61163	novel
P12830	P62736	novel
P12830	P63261	known
P12830	P63267	novel
P12830	P68032	novel
P12830	P68133	novel
P12830	Q08043	novel
P12830	Q156A1	novel
P12830	Q562R1	novel
P12830	Q96QZ7	known
P12830	Q9BYD9	novel
P12830	Q9BYX7	novel
P15311	P22223	novel
P15311	P42025	novel
P15311	P60709	known
P15311	P61163	novel
P15311	P62736	novel
P15311	P63261	novel
P15311	P63267	novel
P15311	P68032	known
P15311	P68133	novel
P15311	P78352	novel
P15311	Q12959	known
P15311	Q156A1	novel
P15311	Q15700	novel
P15311	Q562R1	novel
P15311	Q92796	novel
P15311	Q9BYD9	novel
P15311	Q9BYX7	novel
P22223	P35221	known
P22223	P60709	novel
P22223	P62736	novel
P22223	P63261	novel
P22223	P63267	novel
P22223	P68032	novel
P22223	P68133	novel
P22223	Q156A1	novel
P22223	Q562R1	novel
P22223	Q96QZ7	novel
P22223	Q9BYX7	novel
P35221	P35609	novel
P35221	P78352	novel
P35221	Q08043	novel
P35221	Q12959	known
P35221	Q15700	novel
P35221	Q92796	novel
P35609	P42025	novel
P35609	P60709	novel
P35609	P61163	novel
P35609	P62736	novel
P35609	P63261	novel
P35609	P63267	novel
P35609	P68032	novel
P35609	P68133	novel
P35609	P78352	known
P35609	Q08043	known
P35609	Q12959	known
P35609	Q156A1	novel
P35609	Q15700	novel
P35609	Q562R1	novel
P35609	Q92796	novel

P35609	Q96QZ7	novel
P35609	Q9BYD9	novel
P35609	Q9BYX7	novel
P42025	P60709	novel
P42025	P61163	novel
P42025	P62736	novel
P42025	P63261	novel
P42025	P63267	novel
P42025	P68032	novel
P42025	P68133	known
P42025	Q08043	novel
P42025	Q12959	novel
P42025	Q156A1	novel
P42025	Q562R1	novel
P42025	Q9BYD9	novel
P42025	Q9BYX7	novel
P60709	P61163	novel
P60709	P62736	novel
P60709	P63261	known
P60709	P63267	novel
P60709	P68032	known
P60709	P68133	novel
P60709	P78352	novel
P60709	Q08043	novel
P60709	Q12959	novel
P60709	Q156A1	novel
P60709	Q15700	novel
P60709	Q562R1	novel
P60709	Q92796	novel
P60709	Q9BYD9	novel
P60709	Q9BYX7	novel
P61163	P62736	novel
P61163	P63261	novel
P61163	P63267	novel
P61163	P68032	novel
P61163	P68133	novel
P61163	Q08043	novel
P61163	Q12959	novel
P61163	Q156A1	novel
P61163	Q562R1	novel
P61163	Q9BYD9	novel
P61163	Q9BYX7	novel
P62736	P63261	novel
P62736	P63267	novel
P62736	P68032	novel
P62736	P68133	novel
P62736	P78352	novel
P62736	Q08043	novel
P62736	Q12959	novel
P62736	Q156A1	novel
P62736	Q15700	novel
P62736	Q562R1	novel
P62736	Q92796	novel
P62736	Q9BYD9	novel
P62736	Q9BYX7	novel
P63261	P63267	novel
P63261	P68032	novel
P63261	P68133	novel
P63261	P78352	novel
P63261	Q08043	novel
P63261	Q12959	novel
P63261	Q156A1	novel
P63261	Q15700	novel
P63261	Q562R1	novel
P63261	Q92796	novel
P63261	Q9BYD9	novel
P63261	Q9BYX7	novel
P63267	P68032	novel
P63267	P68133	novel
P63267	P78352	novel

P63267	Q08043	novel
P63267	Q12959	novel
P63267	Q156A1	novel
P63267	Q15700	novel
P63267	Q562R1	novel
P63267	Q92796	novel
P63267	Q9BYD9	novel
P63267	Q9BYX7	novel
P68032	P68133	novel
P68032	P78352	novel
P68032	Q08043	novel
P68032	Q12959	novel
P68032	Q156A1	novel
P68032	Q15700	novel
P68032	Q562R1	novel
P68032	Q92796	novel
P68032	Q9BYD9	novel
P68032	Q9BYX7	novel
P68133	P78352	novel
P68133	Q08043	novel
P68133	Q12959	known
P68133	Q156A1	novel
P68133	Q15700	novel
P68133	Q562R1	novel
P68133	Q92796	novel
P68133	Q9BYD9	novel
P68133	Q9BYX7	novel
P78352	Q08043	novel
P78352	Q12959	known
P78352	Q156A1	novel
P78352	Q15700	known
P78352	Q562R1	novel
P78352	Q92796	known
P78352	Q9BYX7	novel
Q08043	Q12959	novel
Q08043	Q156A1	novel
Q08043	Q15700	novel
Q08043	Q562R1	novel
Q08043	Q92796	novel
Q08043	Q96QZ7	novel
Q08043	Q9BYD9	novel
Q08043	Q9BYX7	novel
Q12959	Q156A1	novel
Q12959	Q15700	known
Q12959	Q562R1	novel
Q12959	Q92796	known
Q12959	Q9BYD9	novel
Q12959	Q9BYX7	novel
Q156A1	Q15700	novel
Q156A1	Q562R1	novel
Q156A1	Q92796	novel
Q156A1	Q96QZ7	novel
Q156A1	Q9BYD9	novel
Q156A1	Q9BYX7	novel
Q15700	Q562R1	novel
Q15700	Q92796	novel
Q15700	Q9BYX7	novel
Q562R1	Q92796	novel
Q562R1	Q9BYD9	novel
Q562R1	Q9BYX7	novel
Q92796	Q9BYX7	novel
Q9BYD9	Q9BYX7	novel

(MF)	adherens junction organization	4.84664511e-10	17.2414% (5/29)	0.1421% (32/22513)
(MF)	cell junction assembly	4.98227744e-10	20.6897% (6/29)	0.3331% (75/22513)
(MF)	cell-cell junction organization	9.85577767e-09	17.2414% (5/29)	0.2532% (57/22513)
(BP)	ATP binding	1.12495587e-08	44.8276% (13/29)	6.6273% (1492/22513)
(BP)	integrin binding	4.27579274e-08	17.2414% (5/29)	0.3376% (76/22513)
(MF)	platelet degranulation	5.54402301e-08	17.2414% (5/29)	0.3554% (80/22513)
(MF)	axon guidance	9.66504979e-08	24.1379% (7/29)	1.3592% (306/22513)
(MF)	muscle filament sliding	1.58962300e-07	13.7931% (4/29)	0.1688% (38/22513)

(BP) guanylate kinase activity 3.14884047e-07 10.3448% (3/29) 0.0489% (11/22513)
(BP) nucleotide binding 7.36072775e-07 44.8276% (13/29) 9.4523% (2128/22513)
(BP) protein binding 8.43379830e-07 62.0690% (18/29) 19.9307% (4487/22513)

Paraclique 2 (29 members)
A6NMY6
P00533
P06213
P06731
P07947
P09769
P11464
P11465
P12931
P13688
P29350
P29353
P31997
P40199
P49023
P84022
Q00887
Q00888
Q00889
Q05209
Q06124
Q13046
Q14002
Q15235
Q15238
Q16557
Q96A09
Q9UQ72
Q9UQ74
A6NMY6 P06731 novel
A6NMY6 P07947 novel
A6NMY6 P09769 novel
A6NMY6 P11464 novel
A6NMY6 P11465 novel
A6NMY6 P12931 novel
A6NMY6 P13688 novel
A6NMY6 P31997 novel
A6NMY6 P40199 novel
A6NMY6 Q00887 novel
A6NMY6 Q00888 novel
A6NMY6 Q00889 novel
A6NMY6 Q13046 novel
A6NMY6 Q14002 novel
A6NMY6 Q15235 novel
A6NMY6 Q15238 novel
A6NMY6 Q16557 novel
A6NMY6 Q9UQ72 novel
A6NMY6 Q9UQ74 novel
P00533 P06213 novel
P00533 P06731 novel
P00533 P07947 novel
P00533 P09769 novel
P00533 P11464 novel
P00533 P11465 novel
P00533 P12931 known
P00533 P13688 known
P00533 P29350 known
P00533 P29353 known
P00533 P31997 novel
P00533 P40199 novel
P00533 Q00887 novel
P00533 Q00888 novel
P00533 Q00889 novel
P00533 Q06124 known

P00533	Q13046	novel
P00533	Q14002	novel
P00533	Q15235	novel
P00533	Q15238	novel
P00533	Q16557	novel
P00533	Q9UQ72	novel
P00533	Q9UQ74	novel
P06213	P06731	novel
P06213	P07947	novel
P06213	P09769	novel
P06213	P11464	novel
P06213	P11465	novel
P06213	P12931	known
P06213	P13688	known
P06213	P29350	known
P06213	P29353	known
P06213	P31997	novel
P06213	P40199	novel
P06213	P49023	novel
P06213	P84022	novel
P06213	Q00887	novel
P06213	Q00888	novel
P06213	Q00889	novel
P06213	Q05209	known
P06213	Q06124	known
P06213	Q13046	novel
P06213	Q14002	novel
P06213	Q15235	novel
P06213	Q15238	novel
P06213	Q16557	novel
P06213	Q9UQ72	novel
P06213	Q9UQ74	novel
P06731	P07947	novel
P06731	P09769	novel
P06731	P11464	novel
P06731	P11465	novel
P06731	P12931	novel
P06731	P13688	novel
P06731	P29350	novel
P06731	P29353	novel
P06731	P31997	novel
P06731	P40199	novel
P06731	P49023	novel
P06731	P84022	novel
P06731	Q00887	novel
P06731	Q00888	novel
P06731	Q00889	novel
P06731	Q05209	novel
P06731	Q06124	novel
P06731	Q13046	novel
P06731	Q14002	novel
P06731	Q15235	novel
P06731	Q15238	novel
P06731	Q16557	novel
P06731	Q9UQ72	novel
P06731	Q9UQ74	novel
P07947	P09769	novel
P07947	P11464	novel
P07947	P12931	novel
P07947	P13688	novel
P07947	P29350	novel
P07947	P29353	novel
P07947	P31997	novel
P07947	P40199	novel
P07947	P49023	novel
P07947	Q06124	novel
P07947	Q14002	novel
P09769	P11464	novel
P09769	P12931	known
P09769	P13688	novel

P09769	P29350	novel
P09769	P29353	novel
P09769	P31997	novel
P09769	P40199	novel
P09769	P49023	novel
P09769	Q06124	novel
P09769	Q14002	novel
P11464	P11465	novel
P11464	P12931	novel
P11464	P13688	novel
P11464	P29350	novel
P11464	P29353	novel
P11464	P31997	novel
P11464	P40199	novel
P11464	P49023	novel
P11464	P84022	novel
P11464	Q00888	novel
P11464	Q05209	novel
P11464	Q06124	novel
P11464	Q13046	novel
P11464	Q14002	novel
P11464	Q15235	novel
P11464	Q15238	novel
P11464	Q16557	novel
P11464	Q96A09	novel
P11464	Q9UQ72	novel
P11464	Q9UQ74	novel
P11465	P12931	novel
P11465	P13688	novel
P11465	P29350	novel
P11465	P29353	novel
P11465	P31997	novel
P11465	P40199	novel
P11465	P49023	novel
P11465	P84022	novel
P11465	Q00888	novel
P11465	Q05209	novel
P11465	Q06124	novel
P11465	Q14002	novel
P11465	Q96A09	novel
P12931	P13688	known
P12931	P29350	known
P12931	P29353	known
P12931	P31997	novel
P12931	P40199	novel
P12931	P49023	known
P12931	Q00887	novel
P12931	Q00888	novel
P12931	Q00889	novel
P12931	Q06124	known
P12931	Q13046	novel
P12931	Q14002	novel
P12931	Q15235	novel
P12931	Q15238	novel
P12931	Q16557	novel
P12931	Q9UQ72	novel
P12931	Q9UQ74	novel
P13688	P29350	known
P13688	P29353	known
P13688	P31997	known
P13688	P40199	known
P13688	P49023	known
P13688	Q00887	novel
P13688	Q00888	novel
P13688	Q00889	novel
P13688	Q06124	known
P13688	Q13046	novel
P13688	Q14002	novel
P13688	Q15235	novel
P13688	Q15238	novel

P13688	Q16557	novel
P13688	Q9UQ72	novel
P13688	Q9UQ74	novel
P29350	P29353	known
P29350	P31997	novel
P29350	P40199	novel
P29350	Q00887	novel
P29350	Q00888	novel
P29350	Q00889	novel
P29350	Q06124	known
P29350	Q13046	novel
P29350	Q14002	novel
P29350	Q15235	novel
P29350	Q15238	novel
P29350	Q16557	novel
P29350	Q9UQ72	novel
P29350	Q9UQ74	novel
P29353	P31997	novel
P29353	P40199	novel
P29353	Q00887	novel
P29353	Q00888	novel
P29353	Q00889	novel
P29353	Q05209	known
P29353	Q06124	known
P29353	Q13046	novel
P29353	Q14002	novel
P29353	Q15235	novel
P29353	Q15238	novel
P29353	Q16557	novel
P29353	Q9UQ72	novel
P29353	Q9UQ74	novel
P31997	P40199	known
P31997	P49023	novel
P31997	P84022	novel
P31997	Q00887	novel
P31997	Q00888	novel
P31997	Q00889	novel
P31997	Q05209	novel
P31997	Q06124	novel
P31997	Q13046	novel
P31997	Q14002	novel
P31997	Q15235	novel
P31997	Q15238	novel
P31997	Q16557	novel
P31997	Q9UQ72	novel
P31997	Q9UQ74	novel
P40199	P49023	novel
P40199	P84022	novel
P40199	Q00887	novel
P40199	Q00888	novel
P40199	Q00889	novel
P40199	Q05209	novel
P40199	Q06124	novel
P40199	Q13046	novel
P40199	Q14002	novel
P40199	Q15235	novel
P40199	Q15238	novel
P40199	Q16557	novel
P40199	Q9UQ72	novel
P40199	Q9UQ74	novel
P49023	Q00887	novel
P49023	Q00888	novel
P49023	Q00889	novel
P49023	Q05209	known
P49023	Q06124	known
P49023	Q13046	novel
P49023	Q14002	novel
P49023	Q15235	novel
P49023	Q15238	novel
P49023	Q16557	novel

```
P49023  Q9UQ72  novel
P49023  Q9UQ74  novel
P84022  Q00887  novel
P84022  Q00888  novel
P84022  Q00889  novel
P84022  Q05209  novel
P84022  Q13046  novel
P84022  Q15235  novel
P84022  Q15238  novel
P84022  Q16557  novel
P84022  Q9UQ72  novel
P84022  Q9UQ74  novel
Q00887  Q05209  known
Q00887  Q06124  novel
Q00887  Q14002  novel
Q00888  Q00889  novel
Q00888  Q05209  novel
Q00888  Q06124  novel
Q00888  Q13046  novel
Q00888  Q14002  novel
Q00888  Q15235  novel
Q00888  Q15238  novel
Q00888  Q16557  novel
Q00888  Q96A09  novel
Q00888  Q9UQ72  novel
Q00888  Q9UQ74  novel
Q00889  Q05209  novel
Q00889  Q06124  novel
Q00889  Q14002  novel
Q05209  Q13046  novel
Q05209  Q15235  novel
Q05209  Q15238  novel
Q05209  Q16557  novel
Q05209  Q9UQ72  novel
Q05209  Q9UQ74  novel
Q06124  Q13046  novel
Q06124  Q14002  novel
Q06124  Q15235  novel
Q06124  Q15238  novel
Q06124  Q16557  novel
Q06124  Q9UQ72  novel
Q06124  Q9UQ74  novel
Q13046  Q14002  novel
Q13046  Q15235  novel
Q13046  Q96A09  novel
Q13046  Q9UQ72  novel
Q14002  Q15235  novel
Q14002  Q15238  novel
Q14002  Q16557  novel
Q14002  Q9UQ72  novel
Q14002  Q9UQ74  novel
Q15238  Q96A09  novel
Q16557  Q96A09  novel
(MF)    female pregnancy        1.73638231e-16  31.0345% (9/29) 0.3154% (71/22513)
(BP)    protein tyrosine kinase activity        3.45198727e-09  20.6897% (6/29) 0.4575% (103/22513)
(MF)    peptidyl-tyrosine phosphorylation       5.55890051e-09  17.2414% (5/29) 0.2265% (51/22513)
(MF)    leukocyte migration     2.39344352e-07  17.2414% (5/29) 0.4753% (107/22513)
(MF)    epidermal growth factor receptor signaling pathway      6.07648267e-07  17.2414% (5/29) 0.5730%
(129/22513)


Paraclique 3 (29 members)
O00506
O00743
O43815
O60285
P02811
P17844
P23508
P30153
```

P30154		
P36873		
P60510		
P62714		
P63151		
P67775		
Q00005		
Q13033		
Q14BN4		
Q156A1		
Q5VSL9		
Q66LE6		
Q8WZ74		
Q9BUL8		
Q9NRL3		
Q9NVK5		
Q9P289		
Q9ULQ0		
Q9Y2T4		
Q9Y3A3		
Q9Y6E0		
O00506	O00743	novel
O00506	O43815	known
O00506	P02811	novel
O00506	P17844	novel
O00506	P23508	novel
O00506	P30153	known
O00506	P30154	known
O00506	P36873	novel
O00506	P60510	novel
O00506	P62714	novel
O00506	P67775	known
O00506	Q13033	known
O00506	Q14BN4	known
O00506	Q5VSL9	known
O00506	Q8WZ74	novel
O00506	Q9BUL8	known
O00506	Q9NRL3	known
O00506	Q9NVK5	known
O00506	Q9P289	novel
O00506	Q9ULQ0	known
O00506	Q9Y3A3	known
O00506	Q9Y6E0	known
O00743	O43815	novel
O00743	O60285	novel
O00743	P02811	novel
O00743	P30153	novel
O00743	P30154	novel
O00743	P36873	novel
O00743	P60510	novel
O00743	P62714	novel
O00743	P63151	novel
O00743	P67775	novel
O00743	Q00005	novel
O00743	Q13033	novel
O00743	Q156A1	novel
O00743	Q5VSL9	novel
O00743	Q66LE6	novel
O00743	Q8WZ74	novel
O00743	Q9BUL8	novel
O00743	Q9NRL3	novel
O00743	Q9Y2T4	novel
O00743	Q9Y3A3	novel
O00743	Q9Y6E0	novel
O43815	P23508	known
O43815	P30153	known
O43815	P30154	known
O43815	P60510	novel
O43815	P62714	known
O43815	P63151	novel

O43815	P67775	known
O43815	Q13033	known
O43815	Q14BN4	known
O43815	Q5VSL9	known
O43815	Q66LE6	novel
O43815	Q8WZ74	known
O43815	Q9BUL8	known
O43815	Q9NRL3	known
O43815	Q9NVK5	known
O43815	Q9P289	novel
O43815	Q9ULQ0	known
O43815	Q9Y3A3	known
O43815	Q9Y6E0	known
O60285	P30153	novel
O60285	P30154	known
O60285	P60510	novel
O60285	P62714	novel
O60285	P63151	known
O60285	P67775	known
O60285	Q00005	novel
O60285	Q66LE6	novel
O60285	Q9Y2T4	novel
P02811	P17844	novel
P02811	P30153	novel
P02811	P30154	novel
P02811	P36873	novel
P02811	P60510	novel
P02811	P62714	novel
P02811	P67775	novel
P02811	Q14BN4	novel
P02811	Q156A1	novel
P02811	Q9NRL3	novel
P02811	Q9NVK5	novel
P02811	Q9P289	novel
P02811	Q9Y3A3	novel
P02811	Q9Y6E0	novel
P17844	Q156A1	novel
P17844	Q9P289	novel
P17844	Q9Y6E0	known
P23508	P30153	known
P23508	P30154	novel
P23508	Q13033	known
P23508	Q156A1	novel
P23508	Q5VSL9	known
P23508	Q9BUL8	known
P23508	Q9NRL3	known
P23508	Q9P289	novel
P23508	Q9ULQ0	novel
P23508	Q9Y6E0	known
P30153	P30154	known
P30153	P60510	known
P30153	P62714	known
P30153	P63151	known
P30153	P67775	known
P30153	Q00005	novel
P30153	Q13033	known
P30153	Q156A1	novel
P30153	Q5VSL9	known
P30153	Q66LE6	known
P30153	Q8WZ74	known
P30153	Q9BUL8	known
P30153	Q9NRL3	known
P30153	Q9P289	novel
P30153	Q9ULQ0	novel
P30153	Q9Y2T4	known
P30153	Q9Y3A3	known
P30153	Q9Y6E0	known
P30154	P60510	novel
P30154	P62714	known
P30154	P63151	known

P30154	P67775	known
P30154	Q00005	novel
P30154	Q13033	novel
P30154	Q156A1	novel
P30154	Q5VSL9	novel
P30154	Q66LE6	novel
P30154	Q8WZ74	novel
P30154	Q9BUL8	known
P30154	Q9NRL3	novel
P30154	Q9P289	novel
P30154	Q9ULQ0	novel
P30154	Q9Y2T4	known
P30154	Q9Y3A3	known
P30154	Q9Y6E0	known
P36873	P60510	novel
P36873	P62714	novel
P36873	P67775	novel
P36873	Q156A1	novel
P36873	Q9Y6E0	novel
P60510	P62714	known
P60510	P63151	novel
P60510	P67775	novel
P60510	Q00005	novel
P60510	Q13033	novel
P60510	Q156A1	novel
P60510	Q5VSL9	novel
P60510	Q66LE6	novel
P60510	Q8WZ74	novel
P60510	Q9BUL8	novel
P60510	Q9NRL3	novel
P60510	Q9P289	novel
P60510	Q9ULQ0	novel
P60510	Q9Y2T4	novel
P60510	Q9Y3A3	novel
P60510	Q9Y6E0	novel
P62714	P63151	known
P62714	P67775	novel
P62714	Q00005	novel
P62714	Q13033	known
P62714	Q156A1	novel
P62714	Q5VSL9	known
P62714	Q66LE6	novel
P62714	Q8WZ74	known
P62714	Q9BUL8	novel
P62714	Q9NRL3	known
P62714	Q9P289	novel
P62714	Q9ULQ0	novel
P62714	Q9Y2T4	novel
P62714	Q9Y3A3	known
P62714	Q9Y6E0	known
P63151	P67775	known
P63151	Q13033	known
P63151	Q156A1	novel
P67775	Q00005	novel
P67775	Q13033	known
P67775	Q156A1	novel
P67775	Q5VSL9	known
P67775	Q66LE6	known
P67775	Q8WZ74	known
P67775	Q9BUL8	known
P67775	Q9NRL3	known
P67775	Q9P289	novel
P67775	Q9ULQ0	novel
P67775	Q9Y2T4	known
P67775	Q9Y3A3	known
P67775	Q9Y6E0	known
Q00005	Q13033	novel
Q00005	Q156A1	novel
Q13033	Q14BN4	known
Q13033	Q5VSL9	known

Q13033 Q66LE6 novel
Q13033 Q8WZ74 known
Q13033 Q9BUL8 known
Q13033 Q9NRL3 known
Q13033 Q9NVK5 known
Q13033 Q9P289 novel
Q13033 Q9ULQ0 known
Q13033 Q9Y2T4 novel
Q13033 Q9Y3A3 known
Q13033 Q9Y6E0 known
Q14BN4 Q5VSL9 known
Q14BN4 Q9BUL8 known
Q14BN4 Q9P289 novel
Q14BN4 Q9ULQ0 novel
Q14BN4 Q9Y3A3 known
Q14BN4 Q9Y6E0 known
Q156A1 Q66LE6 novel
Q156A1 Q9NRL3 novel
Q156A1 Q9Y2T4 novel
Q5VSL9 Q8WZ74 known
Q5VSL9 Q9BUL8 known
Q5VSL9 Q9NRL3 known
Q5VSL9 Q9NVK5 known
Q5VSL9 Q9P289 novel
Q5VSL9 Q9ULQ0 known
Q5VSL9 Q9Y3A3 known
Q5VSL9 Q9Y6E0 known
Q8WZ74 Q9BUL8 known
Q8WZ74 Q9NRL3 known
Q8WZ74 Q9P289 novel
Q8WZ74 Q9ULQ0 known
Q8WZ74 Q9Y3A3 known
Q8WZ74 Q9Y6E0 known
Q9BUL8 Q9NRL3 known
Q9BUL8 Q9NVK5 known
Q9BUL8 Q9P289 novel
Q9BUL8 Q9ULQ0 novel
Q9BUL8 Q9Y3A3 known
Q9BUL8 Q9Y6E0 known
Q9NRL3 Q9P289 novel
Q9NRL3 Q9ULQ0 novel
Q9NRL3 Q9Y3A3 known
Q9NRL3 Q9Y6E0 known
Q9NVK5 Q9P289 novel
Q9NVK5 Q9ULQ0 novel
Q9NVK5 Q9Y3A3 known
Q9NVK5 Q9Y6E0 known
Q9P289 Q9ULQ0 novel
Q9P289 Q9Y3A3 novel
Q9P289 Q9Y6E0 novel
Q9ULQ0 Q9Y3A3 known
Q9ULQ0 Q9Y6E0 known
Q9Y3A3 Q9Y6E0 known
(BP)   protein serine/threonine phosphatase activity   4.98718557e-12 20.6897% (6/29) 0.1599% (36/22513)
(BP)   protein phosphatase type 2A regulator activity 2.83112314e-11 17.2414% (5/29) 0.0844% (19/22513)
(BP)   protein binding 1.50499303e-09 72.4138% (21/29)   19.9307% (4487/22513)
(MF)   protein dephosphorylation   3.45198727e-09 20.6897% (6/29) 0.4575% (103/22513)
(BP)   armadillo repeat domain binding 2.29205105e-07 10.3448% (3/29) 0.0444% (10/22513)
(BP)   protein phosphatase 2A binding 8.65312332e-07 10.3448% (3/29) 0.0666% (15/22513)

Paraclique 4 (29 members)
O43707
P00533
P02811
P07948
P12814
P12830
P15311
P17252

P28223		
P35609		
P42025		
P60709		
P61163		
P62736		
P63261		
P63267		
P68032		
P68133		
P78352		
P78559		
Q08043		
Q12959		
Q156A1		
Q15700		
Q562R1		
Q92796		
Q9BYD9		
Q9BYX7		
Q9C0D5		
O43707	P02811	novel
O43707	P12814	novel
O43707	P12830	novel
O43707	P35609	novel
O43707	P42025	novel
O43707	P60709	novel
O43707	P61163	novel
O43707	P62736	novel
O43707	P63261	novel
O43707	P63267	novel
O43707	P68032	known
O43707	P68133	novel
O43707	P78352	novel
O43707	Q08043	known
O43707	Q12959	novel
O43707	Q156A1	novel
O43707	Q15700	novel
O43707	Q562R1	novel
O43707	Q92796	novel
O43707	Q9BYD9	novel
O43707	Q9BYX7	novel
P00533	P02811	novel
P00533	P07948	known
P00533	P12830	known
P00533	P15311	known
P00533	P17252	known
P00533	P42025	novel
P00533	P60709	novel
P00533	P61163	novel
P00533	P62736	novel
P00533	P63261	novel
P00533	P63267	novel
P00533	P68032	novel
P00533	P68133	known
P00533	P78352	novel
P00533	Q12959	novel
P00533	Q156A1	novel
P00533	Q15700	novel
P00533	Q562R1	novel
P00533	Q92796	novel
P00533	Q9BYD9	novel
P00533	Q9BYX7	novel
P02811	P07948	novel
P02811	P12814	novel
P02811	P12830	novel
P02811	P17252	novel
P02811	P35609	novel
P02811	P42025	novel
P02811	P60709	novel

P02811	P61163	novel
P02811	P62736	novel
P02811	P63261	novel
P02811	P63267	novel
P02811	P68032	novel
P02811	P68133	novel
P02811	P78352	novel
P02811	Q08043	novel
P02811	Q12959	novel
P02811	Q156A1	novel
P02811	Q15700	novel
P02811	Q562R1	novel
P02811	Q92796	novel
P02811	Q9BYD9	novel
P02811	Q9BYX7	novel
P07948	P12830	novel
P07948	P15311	novel
P07948	P17252	novel
P07948	P42025	novel
P07948	P60709	known
P07948	P61163	novel
P07948	P62736	novel
P07948	P63261	novel
P07948	P63267	novel
P07948	P68032	novel
P07948	P68133	novel
P07948	P78352	known
P07948	Q12959	novel
P07948	Q156A1	novel
P07948	Q15700	novel
P07948	Q562R1	novel
P07948	Q92796	novel
P07948	Q9BYD9	novel
P07948	Q9BYX7	novel
P12814	P12830	known
P12814	P35609	novel
P12814	P42025	novel
P12814	P60709	novel
P12814	P61163	novel
P12814	P62736	novel
P12814	P63261	novel
P12814	P63267	novel
P12814	P68032	novel
P12814	P68133	novel
P12814	P78352	novel
P12814	Q08043	novel
P12814	Q12959	novel
P12814	Q156A1	novel
P12814	Q15700	novel
P12814	Q562R1	novel
P12814	Q92796	novel
P12814	Q9BYD9	novel
P12814	Q9BYX7	novel
P12830	P15311	known
P12830	P35609	novel
P12830	P42025	novel
P12830	P60709	novel
P12830	P61163	novel
P12830	P62736	novel
P12830	P63261	known
P12830	P63267	novel
P12830	P68032	novel
P12830	P68133	novel
P12830	Q08043	novel
P12830	Q156A1	novel
P12830	Q562R1	novel
P12830	Q9BYD9	novel
P12830	Q9BYX7	novel
P15311	P17252	known
P15311	P42025	novel

P15311	P60709	known
P15311	P61163	novel
P15311	P62736	novel
P15311	P63261	novel
P15311	P63267	novel
P15311	P68032	known
P15311	P68133	novel
P15311	P78352	novel
P15311	Q12959	known
P15311	Q156A1	novel
P15311	Q15700	novel
P15311	Q562R1	novel
P15311	Q92796	novel
P15311	Q9BYD9	novel
P15311	Q9BYX7	novel
P17252	P42025	novel
P17252	P60709	novel
P17252	P61163	novel
P17252	P62736	novel
P17252	P63261	novel
P17252	P63267	novel
P17252	P68032	novel
P17252	P68133	known
P17252	P78352	known
P17252	Q12959	novel
P17252	Q156A1	novel
P17252	Q15700	novel
P17252	Q562R1	novel
P17252	Q92796	novel
P17252	Q9BYD9	novel
P17252	Q9BYX7	novel
P28223	P78352	known
P28223	P78559	known
P28223	Q15700	novel
P28223	Q92796	novel
P35609	P42025	novel
P35609	P60709	novel
P35609	P61163	novel
P35609	P62736	novel
P35609	P63261	novel
P35609	P63267	novel
P35609	P68032	novel
P35609	P68133	novel
P35609	P78352	known
P35609	Q08043	known
P35609	Q12959	known
P35609	Q156A1	novel
P35609	Q15700	novel
P35609	Q562R1	novel
P35609	Q92796	novel
P35609	Q9BYD9	novel
P35609	Q9BYX7	novel
P42025	P60709	novel
P42025	P61163	novel
P42025	P62736	novel
P42025	P63261	novel
P42025	P63267	novel
P42025	P68032	novel
P42025	P68133	known
P42025	P78559	novel
P42025	Q08043	novel
P42025	Q12959	novel
P42025	Q156A1	novel
P42025	Q562R1	novel
P42025	Q9BYD9	novel
P42025	Q9BYX7	novel
P60709	P61163	novel
P60709	P62736	novel
P60709	P63261	known
P60709	P63267	novel

P60709	P68032	known
P60709	P68133	novel
P60709	P78352	novel
P60709	P78559	novel
P60709	Q08043	novel
P60709	Q12959	novel
P60709	Q156A1	novel
P60709	Q15700	novel
P60709	Q562R1	novel
P60709	Q92796	novel
P60709	Q9BYD9	novel
P60709	Q9BYX7	novel
P60709	Q9C0D5	known
P61163	P62736	novel
P61163	P63261	novel
P61163	P63267	novel
P61163	P68032	novel
P61163	P68133	novel
P61163	P78559	novel
P61163	Q08043	novel
P61163	Q12959	novel
P61163	Q156A1	novel
P61163	Q562R1	novel
P61163	Q9BYD9	novel
P61163	Q9BYX7	novel
P61163	Q9C0D5	novel
P62736	P63261	novel
P62736	P63267	novel
P62736	P68032	novel
P62736	P68133	novel
P62736	P78352	novel
P62736	P78559	novel
P62736	Q08043	novel
P62736	Q12959	novel
P62736	Q156A1	novel
P62736	Q15700	novel
P62736	Q562R1	novel
P62736	Q92796	novel
P62736	Q9BYD9	novel
P62736	Q9BYX7	novel
P62736	Q9C0D5	novel
P63261	P63267	novel
P63261	P68032	novel
P63261	P68133	novel
P63261	P78352	novel
P63261	P78559	known
P63261	Q08043	novel
P63261	Q12959	novel
P63261	Q156A1	novel
P63261	Q15700	novel
P63261	Q562R1	novel
P63261	Q92796	novel
P63261	Q9BYD9	novel
P63261	Q9BYX7	novel
P63261	Q9C0D5	novel
P63267	P68032	novel
P63267	P68133	novel
P63267	P78352	novel
P63267	P78559	novel
P63267	Q08043	novel
P63267	Q12959	novel
P63267	Q156A1	novel
P63267	Q15700	novel
P63267	Q562R1	novel
P63267	Q92796	novel
P63267	Q9BYD9	novel
P63267	Q9BYX7	novel
P63267	Q9C0D5	novel
P68032	P68133	novel
P68032	P78352	novel

P68032	P78559	novel
P68032	Q08043	novel
P68032	Q12959	novel
P68032	Q156A1	novel
P68032	Q15700	novel
P68032	Q562R1	novel
P68032	Q92796	novel
P68032	Q9BYD9	novel
P68032	Q9BYX7	novel
P68032	Q9C0D5	novel
P68133	P78352	novel
P68133	P78559	known
P68133	Q08043	novel
P68133	Q12959	known
P68133	Q156A1	novel
P68133	Q15700	novel
P68133	Q562R1	novel
P68133	Q92796	novel
P68133	Q9BYD9	novel
P68133	Q9BYX7	novel
P68133	Q9C0D5	novel
P78352	P78559	known
P78352	Q08043	novel
P78352	Q12959	known
P78352	Q156A1	novel
P78352	Q15700	known
P78352	Q562R1	novel
P78352	Q92796	known
P78352	Q9BYX7	novel
P78352	Q9C0D5	known
P78559	Q12959	known
P78559	Q156A1	novel
P78559	Q15700	known
P78559	Q562R1	novel
P78559	Q92796	novel
P78559	Q9BYD9	novel
P78559	Q9BYX7	novel
Q08043	Q12959	novel
Q08043	Q156A1	novel
Q08043	Q15700	novel
Q08043	Q562R1	novel
Q08043	Q92796	novel
Q08043	Q9BYD9	novel
Q08043	Q9BYX7	novel
Q12959	Q156A1	novel
Q12959	Q15700	known
Q12959	Q562R1	novel
Q12959	Q92796	known
Q12959	Q9BYD9	novel
Q12959	Q9BYX7	novel
Q12959	Q9C0D5	known
Q156A1	Q15700	novel
Q156A1	Q562R1	novel
Q156A1	Q92796	novel
Q156A1	Q9BYD9	novel
Q156A1	Q9BYX7	novel
Q156A1	Q9C0D5	novel
Q15700	Q562R1	novel
Q15700	Q92796	novel
Q15700	Q9BYX7	novel
Q15700	Q9C0D5	novel
Q562R1	Q92796	novel
Q562R1	Q9BYD9	novel
Q562R1	Q9BYX7	novel
Q562R1	Q9C0D5	novel
Q92796	Q9BYX7	novel
Q92796	Q9C0D5	novel
Q9BYD9	Q9BYX7	novel
Q9BYD9	Q9C0D5	novel
Q9BYX7	Q9C0D5	novel

(BP) ATP binding 1.12495587e-08 44.8276% (13/29) 6.6273% (1492/22513)
(BP) integrin binding 4.27579274e-08 17.2414% (5/29) 0.3376% (76/22513)
(MF) platelet degranulation 5.54402301e-08 17.2414% (5/29) 0.3554% (80/22513)
(MF) blood coagulation 8.00650746e-08 27.5862% (8/29) 2.0299% (457/22513)
(MF) axon guidance 9.66504979e-08 24.1379% (7/29) 1.3592% (306/22513)
(MF) muscle filament sliding 1.58962300e-07 13.7931% (4/29) 0.1688% (38/22513)
(MF) platelet activation 2.05330328e-07 20.6897% (6/29) 0.9061% (204/22513)
(BP) guanylate kinase activity 3.14884047e-07 10.3448% (3/29) 0.0489% (11/22513)
(MF) synaptic transmission 4.98447848e-07 24.1379% (7/29) 1.7323% (390/22513)
(BP) nucleotide binding 7.36072775e-07 44.8276% (13/29) 9.4523% (2128/22513)
(BP) protein binding 8.43379830e-07 62.0690% (18/29) 19.9307% (4487/22513)

Paraclique 5 (29 members)
P04637
P06493
P08107
P11021
P11142
P17066
P24941
P27348
P31946
P31947
P34931
P36873
P48741
P54652
P61981
P62136
P62140
P62258
P62877
P62988
P63104
Q00526
Q04917
Q13618
Q13619
Q13620
Q156A1
Q15843
Q99459
P04637 P06493 known
P04637 P08107 novel
P04637 P11021 novel
P04637 P11142 known
P04637 P17066 novel
P04637 P24941 known
P04637 P27348 novel
P04637 P31946 novel
P04637 P31947 known
P04637 P34931 novel
P04637 P36873 novel
P04637 P48741 novel
P04637 P54652 novel
P04637 P61981 known
P04637 P62136 known
P04637 P62140 novel
P04637 P62258 novel
P04637 P62988 novel
P04637 P63104 known
P04637 Q00526 novel
P04637 Q04917 novel
P04637 Q156A1 novel
P04637 Q15843 known
P06493 P08107 novel
P06493 P11021 novel
P06493 P11142 novel
P06493 P17066 novel

P06493	P24941	novel
P06493	P27348	novel
P06493	P31946	novel
P06493	P31947	known
P06493	P34931	novel
P06493	P36873	novel
P06493	P48741	novel
P06493	P54652	known
P06493	P61981	novel
P06493	P62136	novel
P06493	P62258	novel
P06493	P62988	novel
P06493	P63104	novel
P06493	Q00526	novel
P06493	Q04917	novel
P06493	Q156A1	novel
P06493	Q15843	novel
P06493	Q99459	novel
P08107	P11021	novel
P08107	P11142	novel
P08107	P17066	novel
P08107	P24941	novel
P08107	P27348	novel
P08107	P31946	known
P08107	P31947	novel
P08107	P34931	novel
P08107	P36873	novel
P08107	P48741	novel
P08107	P54652	novel
P08107	P61981	novel
P08107	P62136	novel
P08107	P62140	novel
P08107	P62258	novel
P08107	P62877	novel
P08107	P62988	novel
P08107	P63104	known
P08107	Q00526	novel
P08107	Q04917	novel
P08107	Q156A1	novel
P08107	Q15843	novel
P08107	Q99459	novel
P11021	P11142	novel
P11021	P17066	novel
P11021	P24941	novel
P11021	P27348	novel
P11021	P31946	known
P11021	P31947	novel
P11021	P34931	novel
P11021	P36873	novel
P11021	P48741	novel
P11021	P54652	novel
P11021	P61981	novel
P11021	P62136	novel
P11021	P62140	novel
P11021	P62258	novel
P11021	P62877	novel
P11021	P62988	novel
P11021	P63104	novel
P11021	Q00526	novel
P11021	Q04917	novel
P11021	Q156A1	novel
P11021	Q15843	novel
P11021	Q99459	novel
P11142	P17066	novel
P11142	P24941	novel
P11142	P27348	known
P11142	P31946	novel
P11142	P31947	novel
P11142	P34931	novel
P11142	P36873	novel

P11142	P48741	novel
P11142	P54652	novel
P11142	P61981	known
P11142	P62136	known
P11142	P62140	novel
P11142	P62258	novel
P11142	P62877	known
P11142	P62988	known
P11142	P63104	novel
P11142	Q00526	novel
P11142	Q04917	novel
P11142	Q156A1	novel
P11142	Q15843	novel
P11142	Q99459	known
P17066	P24941	novel
P17066	P27348	novel
P17066	P31946	novel
P17066	P31947	novel
P17066	P34931	novel
P17066	P36873	novel
P17066	P48741	novel
P17066	P54652	novel
P17066	P61981	novel
P17066	P62136	novel
P17066	P62140	novel
P17066	P62258	novel
P17066	P62877	novel
P17066	P62988	novel
P17066	P63104	novel
P17066	Q00526	novel
P17066	Q04917	novel
P17066	Q156A1	novel
P17066	Q15843	novel
P17066	Q99459	novel
P24941	P27348	novel
P24941	P31946	novel
P24941	P31947	novel
P24941	P34931	novel
P24941	P36873	novel
P24941	P48741	novel
P24941	P54652	novel
P24941	P61981	novel
P24941	P62136	novel
P24941	P62140	novel
P24941	P62258	novel
P24941	P62988	novel
P24941	P63104	novel
P24941	Q00526	novel
P24941	Q04917	novel
P24941	Q156A1	novel
P24941	Q15843	known
P24941	Q99459	known
P27348	P31946	novel
P27348	P31947	novel
P27348	P34931	novel
P27348	P36873	novel
P27348	P48741	novel
P27348	P54652	novel
P27348	P61981	known
P27348	P62136	novel
P27348	P62140	novel
P27348	P62258	novel
P27348	P62988	novel
P27348	P63104	novel
P27348	Q00526	novel
P27348	Q04917	novel
P27348	Q156A1	novel
P27348	Q15843	novel
P27348	Q99459	novel
P31946	P31947	novel

P31946	P34931	novel
P31946	P36873	novel
P31946	P48741	novel
P31946	P54652	novel
P31946	P61981	known
P31946	P62136	novel
P31946	P62140	novel
P31946	P62258	known
P31946	P62988	known
P31946	P63104	novel
P31946	Q00526	novel
P31946	Q04917	novel
P31946	Q156A1	novel
P31946	Q15843	novel
P31946	Q99459	novel
P31947	P34931	novel
P31947	P36873	novel
P31947	P48741	novel
P31947	P54652	novel
P31947	P61981	known
P31947	P62136	novel
P31947	P62140	novel
P31947	P62258	novel
P31947	P62988	novel
P31947	P63104	novel
P31947	Q00526	novel
P31947	Q04917	novel
P31947	Q156A1	novel
P31947	Q15843	novel
P31947	Q99459	novel
P34931	P36873	novel
P34931	P48741	novel
P34931	P54652	novel
P34931	P61981	novel
P34931	P62136	novel
P34931	P62140	novel
P34931	P62258	novel
P34931	P62877	novel
P34931	P62988	novel
P34931	P63104	novel
P34931	Q00526	novel
P34931	Q04917	novel
P34931	Q156A1	novel
P34931	Q15843	novel
P34931	Q99459	novel
P36873	P48741	novel
P36873	P54652	novel
P36873	P61981	novel
P36873	P62136	novel
P36873	P62140	novel
P36873	P62258	novel
P36873	P63104	known
P36873	Q00526	novel
P36873	Q04917	novel
P36873	Q156A1	novel
P36873	Q99459	novel
P48741	P54652	novel
P48741	P61981	novel
P48741	P62136	novel
P48741	P62140	novel
P48741	P62258	novel
P48741	P62877	novel
P48741	P62988	novel
P48741	P63104	novel
P48741	Q00526	novel
P48741	Q04917	novel
P48741	Q156A1	novel
P48741	Q15843	novel
P48741	Q99459	novel
P54652	P61981	novel

P54652	P62136	novel
P54652	P62140	novel
P54652	P62258	novel
P54652	P62877	novel
P54652	P62988	novel
P54652	P63104	novel
P54652	Q00526	novel
P54652	Q04917	novel
P54652	Q156A1	novel
P54652	Q15843	novel
P54652	Q99459	novel
P61981	P62136	novel
P61981	P62140	novel
P61981	P62258	known
P61981	P62988	known
P61981	P63104	novel
P61981	Q00526	novel
P61981	Q04917	known
P61981	Q156A1	novel
P61981	Q15843	novel
P61981	Q99459	known
P62136	P62140	novel
P62136	P62258	novel
P62136	P63104	novel
P62136	Q00526	novel
P62136	Q04917	novel
P62136	Q156A1	novel
P62136	Q99459	known
P62140	P62258	novel
P62140	P63104	novel
P62140	Q00526	novel
P62140	Q04917	novel
P62140	Q156A1	novel
P62140	Q99459	novel
P62258	P62988	novel
P62258	P63104	known
P62258	Q00526	novel
P62258	Q04917	known
P62258	Q156A1	novel
P62258	Q15843	novel
P62258	Q99459	novel
P62877	P62988	novel
P62877	Q13618	known
P62877	Q13619	known
P62877	Q13620	known
P62877	Q15843	known
P62988	P63104	known
P62988	Q00526	novel
P62988	Q04917	novel
P62988	Q13618	novel
P62988	Q13619	novel
P62988	Q13620	novel
P62988	Q156A1	novel
P62988	Q15843	novel
P63104	Q00526	novel
P63104	Q04917	novel
P63104	Q156A1	novel
P63104	Q15843	novel
P63104	Q99459	novel
Q00526	Q04917	novel
Q00526	Q156A1	novel
Q00526	Q15843	novel
Q00526	Q99459	novel
Q04917	Q156A1	novel
Q04917	Q15843	novel
Q04917	Q99459	novel
Q13618	Q15843	known
Q13619	Q15843	known
Q13620	Q15843	known
Q156A1	Q15843	novel

Q156A1 Q99459 novel

(BP)	protein domain specific binding	2.75142304e-13	31.0345%	(9/29)	0.6974%	(157/22513)
(MF)	response to unfolded protein	5.14002573e-11	20.6897%	(6/29)	0.2310%	(52/22513)
(BP)	ubiquitin protein ligase binding	7.82601760e-11	24.1379%	(7/29)	0.4886%	(110/22513)
(BP)	protein binding	1.33533847e-10	75.8621%	(22/29)	19.9307%	(4487/22513)
(MF)	protein targeting	5.94384882e-08	13.7931%	(4/29)	0.1333%	(30/22513)
(MF)	induction of apoptosis by intracellular signals	2.90787833e-07	13.7931%	(4/29)	0.1954%	(44/22513)
(BP)	cyclin binding	5.44853972e-07	10.3448%	(3/29)	0.0577%	(13/22513)
(MF)	G1/S transition of mitotic cell cycle	9.77846624e-07	17.2414%	(5/29)	0.6307%	(142/22513)

... (continued)
